# Anticoagulation options for continuous renal replacement therapy in critically ill patients: a systematic review and network meta-analysis of randomized controlled trials

**DOI:** 10.1186/s13054-023-04519-1

**Published:** 2023-06-07

**Authors:** Zhifeng Zhou, Chen Liu, Yingying Yang, Fang Wang, Ling Zhang, Ping Fu

**Affiliations:** Department of Nephrology, Kidney Research Institute, West China Hospital of Sichuan University, Chengdu, 610041 China

**Keywords:** Continuous renal replacement therapy, Anticoagulation, Acute kidney injury, Filter lifespan, Network meta-analysis

## Abstract

**Background:**

Continuous renal replacement therapy (CRRT) is a widely used standard therapy for critically ill patients with acute kidney injury (AKI). Despite its effectiveness, treatment is often interrupted due to clot formation in the extracorporeal circuits. Anticoagulation is a crucial strategy for preventing extracorporeal circuit clotting during CRRT. While various anticoagulation options are available, there were still no studies synthetically comparing the efficacy and safety of these anticoagulation options.

**Methods:**

Electronic databases (PubMed, Embase, Web of Science, and the Cochrane database) were searched from inception to October 31, 2022. All randomized controlled trials (RCTs) that examined the following outcomes were included: filter lifespan, all-cause mortality, length of stay, duration of CRRT, recovery of kidney function, adverse events and costs.

**Results:**

Thirty-seven RCTs from 38 articles, comprising 2648 participants with 14 comparisons, were included in this network meta-analysis (NMA). Unfractionated heparin (UFH) and regional citrate anticoagulation (RCA) are the most frequently used anticoagulants. Compared to UFH, RCA was found to be more effective in prolonging filter lifespan (MD 12.0, 95% CI 3.8 to 20.2) and reducing the risk of bleeding. Regional-UFH plus Prostaglandin I2 (Regional-UFH + PGI2) appeared to outperform RCA (MD 37.0, 95% CI 12.0 to 62.0), LMWH (MD 41.3, 95% CI 15.6 to 67.0), and other evaluated anticoagulation options in prolonging filter lifespan. However, only a single included RCT with 46 participants had evaluated Regional-UFH + PGI2. No statistically significant difference was observed in terms of length of ICU stay, all-cause mortality, duration of CRRT, recovery of kidney function, and adverse events among most evaluated anticoagulation options.

**Conclusions:**

Compared to UFH, RCA is the preferred anticoagulant for critically ill patients requiring CRRT. The SUCRA analysis and forest plot of Regional-UFH + PGI2 are limited, as only a single study was included. Additional high-quality studies are necessary before any recommendation of Regional-UFH + PGI2. Further larger high-quality RCTs are desirable to strengthen the evidence on the best choice of anticoagulation options to reduce all-cause mortality and adverse events and promote the recovery of kidney function.

*Trial registration* The protocol of this network meta-analysis was registered on PROSPERO (CRD42022360263). Registered 26 September 2022.

**Supplementary Information:**

The online version contains supplementary material available at 10.1186/s13054-023-04519-1.

## Background

Continuous renal replacement therapy (CRRT) is a standard therapy for critically ill patients with acute kidney injury (AKI) in the intensive care unit (ICU) [1]. In contrast to traditional forms of renal replacement therapy (RRT), such as intermittent hemodialysis and peritoneal dialysis, CRRT can deliver solute clearance and acid–base regulation for patients with unstable hemodynamic status [2]. Although CRRT is prescribed as a continuous dialysis therapy, unexpected downtime can occur due to clot formation in the extracorporeal circuits [3]. Frequent clotting in the extracorporeal circuits limits the benefits of CRRT. Extracorporeal circuit clotting can cause potential blood loss that may affect hemodynamic stability and require frequent circuit changes [4, 5], increasing the workload of medical staff and treatment costs [6]. Therefore, effective strategies to prevent clotting in extracorporeal circuits during CRRT are crucial. In addition to catheter choice, blood flow rate, and therapy choice, anticoagulation is also an important and commonly used strategy to prevent clot formation during CRRT [7].

Anticoagulation is essential to prevent extracorporeal circuits clotting during CRRT. Currently, various anticoagulation options have been used in clinical settings, and unfractionated heparin (UFH) is the most commonly used anticoagulant [8] due to its low cost, easy monitoring, and simple reversal [9, 10]. However, the increased risk of bleeding and heparin-induced thrombocytopenia type II (HIT-II) might lead to life-threatening complications [8]. Regional citrate anticoagulation (RCA) is another common anticoagulation option for CRRT. RCA has also been recommended as a suitable form of CRRT anticoagulation for its longer circuit life, and safety even in patients with liver dysfunction [11]. However, potential disturbances, such as citrate toxicity, metabolic alkalosis, and hypocalcemia, might be observed in critically ill patients [12]. Other anticoagulation options such as low-molecular-weight heparin (LMWH), nafamostat mesilate (NM), Prostaglandin I2 (PGI2), and Regional-UFH are also available during CRRT [13–16]. Various anticoagulation options have been used during CRRT, but the efficacy and safety of these anticoagulants remain controversial. To date, no studies have synthetically compared the effects of all these anticoagulation options. Therefore, we conducted a network meta-analysis (NMA) to provide the most recent available evidence on the best choice of anticoagulant during CRRT.

## Methods

We conducted a systematic review with NMA in accordance with the Preferred Reporting Items for Systematic Reviews and Meta-analyses (PRISMA) guidelines [17], and the protocol of this NMA was registered on PROSPERO (CRD42022360263).

### Search strategy

We systematically searched all relevant publications without language restriction in PubMed, Embase, Web of Science, and the Cochrane database from inception to October 31, 2022. The search terms were as follows: “renal replacement therapy,” “continuous venovenous hemofiltration,” “CVVH,” “continuous venovenous hemodialysis’’, “CVVHD’’, “continuous venovenous hemodiafiltration’’, “CVVHDF,” “slow continuous ultrafiltration,” or “SCUF”; “anticoagulation,” “citrate,” “heparin,” “UFH,” “LMWH,” “dalteparin,” “nadroparin,” “enoxaparin,” “bivalirudin,” “prostacyclin,” “nafamostat,” “hirudin,” “iloprost,” or “tirofiban.” In addition, proceedings from the references that were listed in all retrieved articles, and eligible studies from published meta-analyses were also searched to ensure a complete identification of all eligible studies. The detailed search strategy is presented in Additional File 1. The search process was performed and confirmed by two independent reviewers (ZFZ and CL).

### Selection criteria

Participants: Adult critically ill patients receiving CRRT in the ICU.

Interventions: Interventions were regarded as pharmacological interventions for preventing clotting of extracorporeal circuits during CRRT with any type of anticoagulant.

Types of outcome measures: filter lifespan or occurrence of filter clotting, all-cause mortality, length of stay, duration of CRRT, recovery of kidney function, adverse events, and costs.

Type of studies: all randomized controlled trials (RCTs or cluster RCTs) and quasi-RCTs concerning pharmacological anticoagulation during CRRT.

The inclusion criteria were as follows: (1) RCTs concerning pharmacological anticoagulation during CRRT in critically ill patients; (2) studies reporting the following outcomes: filter lifespan or occurrence of filter clotting, all-cause mortality, length of hospital or ICU stay, duration of CRRT, recovery of kidney function, adverse events, and costs; and (3) sufficient data available to calculate risk ratios (RRs) and mean differences (MDs) with 95% confidence intervals (CIs).

Exclusion criteria were: (1) no relevant data; (2) conference abstracts without full-text manuscripts; (3) nonhuman studies or studies that enrolled pediatric patients; (4) studies that enrolled patients receiving dialysis treatment before admission to the ICU; and (5) studies that compared different doses of the same pharmacological anticoagulation.

### Data extraction and quality assessment

Two investigators (ZFZ and YYY) independently screened the titles and abstracts of the articles, and extracted data from the included studies. Any disagreements were resolved by a third author (LZ). The following data from each study were extracted: (1) study characteristics: publication year, author names, study design, target population, number of participants, age, sex, and so on; (2) type of anticoagulation during CRRT, CRRT modalities, dilution, and blood flow rate; and (3) outcome characteristics: filter lifespan or occurrence of filter clotting, all-cause mortality, length of hospital or ICU stay, duration of CRRT, recovery of kidney function, adverse events, and costs. Quality assessment of the included RCTs was performed by two investigators (CL and FW) using the Cochrane Collaboration Risk of Bias Tool [18], and disagreements would be settled by discussion with a third investigator (PF). Selection bias (random sequence generation and allocation concealment), performance bias (blinding of participants and personnel), detection bias (blinding of outcome), attrition bias (incomplete outcome data), reporting bias (selective reporting), and other biases were categorized as “low,” “unclear,” or “high” risk of bias.

### Data synthesis and analysis

RR with 95% CIs was calculated for dichotomous variables, and MD with 95% CIs was calculated for continuous variables. Conventional pairwise meta-analyses were initially conducted using Stata SE, version 14 (StataCorp, College Station, TX, USA), for direct comparisons between different pharmacological anticoagulants during CRRT. *P* < 0.05 was considered statistically significant. Homogeneity, transitivity, and consistency were assumed to underlie the validity of conclusions from NMA analyses [19]. Homogeneity assumption was satisfied when the magnitude of heterogeneity within direct pairwise comparisons was acceptable. Heterogeneity was evaluated using the *I*^2^ statistic. Specifically, *I*^2^ values of 0–24.9%, 25–49.9%, 50–74.9%, and 75–100% represented no, low, moderate, and significant heterogeneity, respectively [20, 21]. And the transitivity assumption was satisfied when studies included were sufficiently similar regarding methodological and clinical characteristics. Inconsistency between direct and indirect estimates in the entire network of each outcome was assessed locally with a loop-specific approach and globally with design-by-treatment interaction model [22, 23]. And the consistency assumption was rejected when the p value of the inconsistency test was less than 0.05. In addition, Begg’s funnel plots and Egger’s test were also performed to analyze potential publication bias when ten or more trials were included in each pairwise meta-analysis. We also identified the relative rankings of different anticoagulants based on the surface under the cumulative ranking curve (SUCRA) for each outcome, ranging from 0 to 100% [24]. And the higher the value, the more effective of the anticoagulation during CRRT.

The NMA was also performed for multiple comparisons using Stata SE, version 14 (StataCorp, College Station, TX, USA). And given the risk of excessively high or low blood flow rates on the robustness of the NMA results, we performed a sensitivity analysis of primary outcome by exclusion of studies with a blood flow rate > 200 ml/min or < 130 ml/min, as well as studies lacking detailed information on blood flow rate. In addition, in order to eliminate the potential influence of other major non-pharmacological interventions (modes of CRRT, and pre- or post-dilution) [25], two subgroup analyses were performed for populations with CVVH, CVVHD, or CVVHDF and pre-dilution or post-dilution, respectively.

## Results

### Search results and characteristics of the included studies

**Fig. 1:**
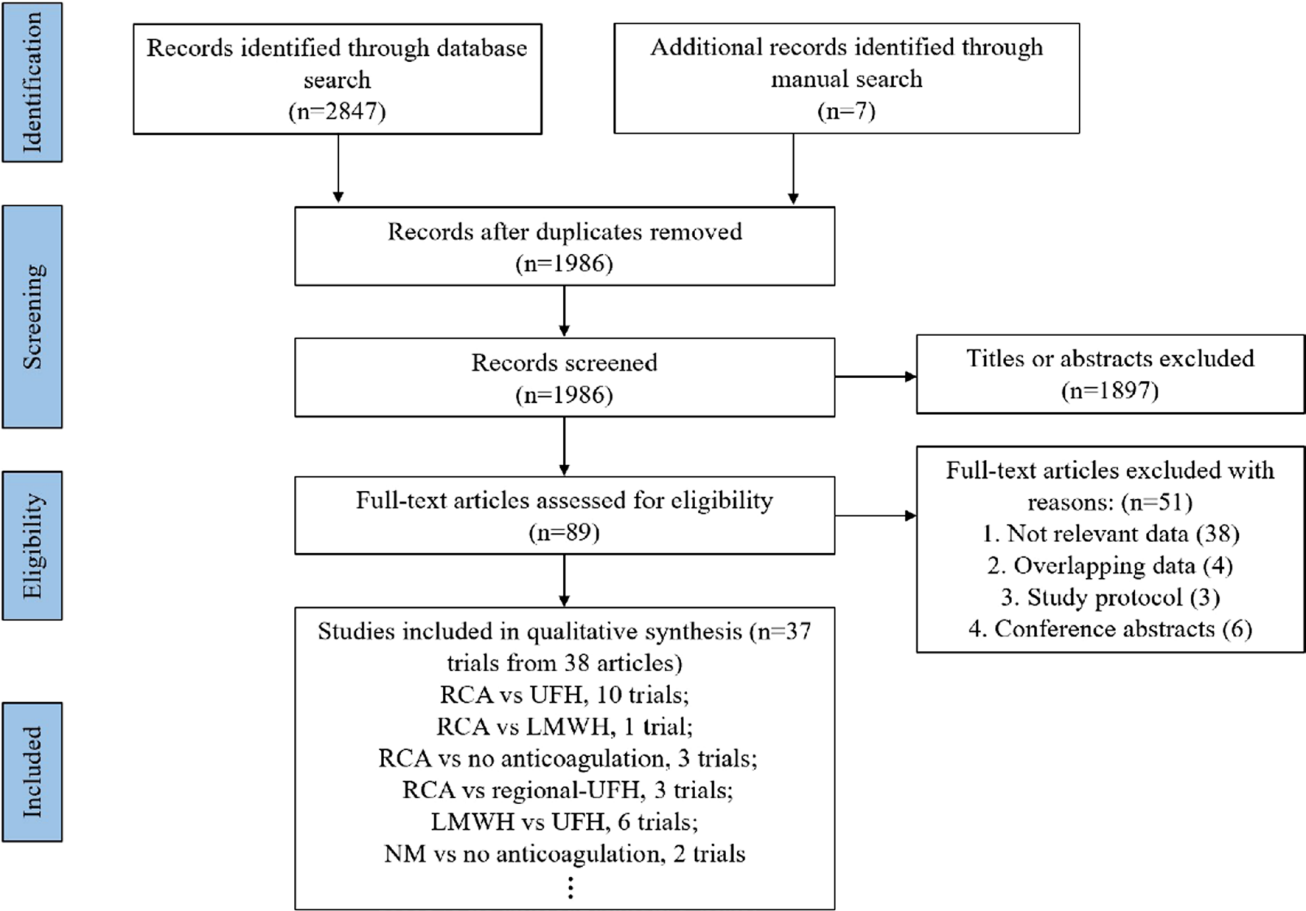
Flowchart of study selection

A flowchart depicting the selection process for this study is presented in Fig. 1. A total of 2854 articles were identified from our initial search, and after removing 868 duplicates, 1986 articles were assessed by screening the title and abstract of each article. Subsequently, 89 articles were read for full text for further evaluation, and 51 articles were excluded for the following reasons: irrelevant data (*n* = 38), overlapping data (*n* = 4), study protocol (*n* = 3), and conference abstracts (*n* = 6). Finally, 37 RCTs from 38 articles were included in our NMA. The eligible studies were conducted from 1993 to 2022 with a total number of 2648 adult patients. These trials were conducted in 14 different countries, with Australia contributing the most (8 trials, 21.6%). And among the included studies, 6 were conducted in multicenter ICUs, and the other 31 RCTs were conducted in a single-center ICU. The NMA evaluated 14 different anticoagulation options, including UFH, RCA, LMWH, NM, PGI2, and so on. Across the analyzed studies, the sample sizes ranged from 10 to 596, and the mean (or median) age of patients ranged from 45 to 78 years. Most studies reported two-arm comparisons, except for 5 studies [26–30] with three-arm comparisons. A range of outcomes were recorded in these studies, including filter lifespan or occurrence of filter clotting, all-cause mortality, length of hospital or ICU stay, duration of CRRT, recovery of kidney function, adverse events, and costs. The baseline characteristics of the included RCTs are presented in Table 1.

**Table 1:** Characteristics of included randomized controlled trials in the network meta-analysis

Study	Study design	Country	Setting	No. of patients	Males (%)	Mean age (y)	Anticoagulation protocol 1	Anticoagulation protocol 2	Anticoagulation protocol 3	Modality; dilution; blood flow (ml–min)
Arcangeli [31]	RCT	Italy	Single-center ICU	10 11	70 64	66 68	Prostacyclin analogue (PGI2)	UFH	–	CVVHDF; pre-dilution; 160–190
Bellomo [26]	RCT	Australia	Single-center ICU	10 13 10	–	–	UFH	Regional-UFH plus protamine	No anticoagulation	CVVHDF; pre-dilution; 150
Betjes [32]	RCT	Netherlands	ICU of a university hospital	21 27	71 70	58 55	RCA	UFH	–	CVVH; post-dilution; 150
Birnbaum [33]	RCT	Germany	ICU at a university hospital	10 10	60 70	74 71	UFH	UFH plus iloprost (PGI2)	–	CVVH; post-dilution; 150
Brain [72]	RCT	Australia	Single-center ICU	19 11	63 64	64 51	RCA	Regional-UFH plus protamine	–	CVVHDF; pre-dilution; mean 191 vs 217
Choi [51]	RCT	South Korea	Single-center ICU	31 24	68 63	64 59	Nafamostat mesilate	No anticoagulation	–	CVVHDF; NR; 150–200
Cui [73]	RCT	China	Single-center ICU	23 23	52 57	47 47	RCA	UFH	–	CVVH; NR; NR
Fabbri [34]	RCT	Italy	Single-center ICU	46 44	48 48	71 70	Regional-UFH plus protamine plus prostacyclin (PGI2)	UFH	–	CVVH; pre-dilution; 80–130
Fealy [52]	RCT (crossover study)	Australia	Single-center ICU	10 10	90	71	RCA	Regional-UFH plus protamine	–	CVVH; pre-dilution; 150
Garcés [13]	RCT	Brazil	Single-center ICU	19 21	37 38	61 54	LMWH (Enoxaparin)	UFH	–	CVVHD; NR; 150
Gattas [47]	RCT	Australia, New Zealand	Multicenter ICUs	105 107	71 67	66 67	RCA	Regional-UFH plus protamine	–	CVVH, CVVHDF; pre-dilution; 150–200
Gao [49]	RCT	China	Single-center ICU	28 28	68 61	60 65	RCA	No anticoagulation	–	CVVH; post-dilution), no (pre- and post-dilution); 150
Hein [35]	RCT	Germany	Single-center ICU	14 12	64 58	72 67	UFH	Hirudin	–	CVVH; post-dilution; 80–150
Hetzel [74]	RCT	Germany	Multicenter ICUs	87 83	66 71	62 65	RCA	UFH	–	CVVH; pre-dilution; 120–150
Joannidis [36]	RCT (crossover study)	France	Single-center ICU	40 40	65	57	LMWH (Enoxaparin)	UFH	–	CVVH; pre-dilution; 180
Kutsogiannis [37]	RCT	Canada	Multicenter ICUs	16 14	44 57	67 64	RCA	UFH	–	CVVHDF; pre-dilution; 125
Kiser [38]	RCT	USA	Single-center ICU	5 5	80 60	58 58	Bivalirudin	UFH	–	CVVH; pre-dilution; 180
Kozek-Langenecker [27]	RCT	Australia	Single-center ICU	17 15 18	–	64 60 62	UFH	UFH plus PGI2	UFH plus prostaglandin E1 (PGE1)	CVVH; pre-dilution; NR
Langenecker [28]	RCT	Australia	Single-center ICU	13 14 19	–	–	UFH	PGI2	UFH plus PGI2	CVVH; pre-dilution; NR
Link [75]	RCT	Germany	Single-center ICU	20 20	60 55	71 70	UFH	UFH plus tirofiban	–	CVVHD; NR; 100–120
Lee [50]	RCT	Korea	Single-center ICU	36 37	67 54	53 58	Nafamostat mesilate	No anticoagulation	–	CVVH; pre-dilution; 130–200
Margraf [39]	RCT	Germany	Single-center ICU	16 20	50 55	62 66	RCA	UFH	–	CVVH; NR; NR
Monchi [40]	RCT	France	Single-center ICU	26 23	46 48	67 64	RCA	UFH	–	CVVH; post-dilution; 150
Oudemans-van Straaten [76]	RCT	Netherlands	Single-center ICU	97 103	68 68	73 73	RCA	LMWH (nadroparin)	–	CVVH; post-dilution; 220
Reeves [53]	RCT	Australia	Single-center ICU	25 22	–	–	LMWH (Dalteparin)	UFH	–	CVVHDF; pre-dilution; 120
Reeves [77]	RCT	Australia	Single-center ICU	15 29	–	78 75	LMWH	UFH	–	CVVHDF; pre-dilution; 125
Schilder [41]	RCT	Netherlands	Multicenter ICUs	66 73	67 67	67 67	RCA	UFH	–	CVVH; pre-dilution; 180
Schilder [29, 78]	RCT	Netherlands	Multicenter ICUs	21 8 13	75 75 54	64 57 70	RCA	UFH	No anticoagulation	CVVH; pre-dilution; 180
Stucker [43]	RCT	Switzerland	Single-center ICU	54 49	59 64	60 65	RCA	UFH	–	CVVHDF; 2–3 pre-dilution and 1–3 post-dilution; 100–200
Tiranathanagul [46]	RCT	Thailand	Single-center ICU	10 10	50 70	70 76	RCA	UFH	–	CVVH; pre-dilution; 120
Trakarnvanich [44]	RCT	Thailand	Two ICUs	21 20	67 55	66 66	RCA	No anticoagulation	–	CVVHDF; post-dilution; 150–200
Vargas Hein [42]	RCT	Germany	Single-center ICU	9 8	89 88	61 67	UFH	Hirudin	–	CVVH; post-dilution; 80–150
van Doorn [79]	RCT	Belgium	Single-center ICU	15 14	60 57	70 63	LMWH (dalteparin)	UFH	–	CVVH; NR; NR
van der Voort [80]	RCT (crossover study)	Belgium	Single-center ICU	15 15	60 60	68 68	Regional-UFH plus protamine	LMWH (nadroparin)	–	CVVH; pre-dilution and post-dilution; 200
Victorino [81]	RCT	Brazil	Single-center ICU	19 21	–	–	LMWH	UFH	–	CVVHD; NR; NR
Wu [30]	RCT	China	Single-center ICU	15 19 19	67 63 74	48 45 46	RCA	LMWH (dalteparin)	RCA plus LMWH (dalteparin)	CVVH; pre-dilution; 180–200
Xun [45]	RCT	China	Single-center ICU	17 14	24 57	78 76	RCA	No anticoagulation	–	CVVH; NR; 150–200
Zarbock [48]	RCT	Germany	Multicenter ICUs	300 296	69 70	68 68	RCA	UFH	–	CVVH, CVVHD, CVVHDF; pre-dilution; > 100

### Risk of bias

For the evaluation of the quality of the included studies, we utilized the Kappa coefficient to test the consistency, and we showed a substantial coefficient with a Kappa value of 0.90. Discrepancies were resolved by a third author through consensus. The details for the risk of bias of individual RCTs are presented in Additional File 2. Overall, most RCTs had a high risk of bias in blinding of participants and personnel, and blinding of outcome due to not being blinded or being conducted as an open-label study. However, blinding of patients and clinicians was clinically impracticable in studies due to virtual practice issues. Specifically, the lowest risk was random sequence generation, with exceeding 70% of studies considered to be at low risk for bias. More than half of the trials were judged as having a low risk of bias in allocation concealment, attrition, and reporting. Additionally, 9 trials had a high risk of other bias due to the funding source or lack of conflict of interest.

### Assessment of heterogeneity, transitivity, and inconsistency

The results of heterogeneity in direct pairwise comparisons are presented in Additional File 3. Overall, no statistically significant heterogeneity was observed in most direct pairwise comparisons, except for some direct comparison pairs for the reduction of creatinine (Cr) and blood urea nitrogen (BUN) (Additional File 3). Publication bias was not evaluated due to the lack of enough trials included in each pair. We have evaluated the inclusion and exclusion criteria of the eligible studies, particularly with respect to adult critically ill patients with AKI (Additional File 4). Moreover, the target anticoagulation levels were comparable among studies. UFH and RCA are the two most commonly used anticoagulants. In the UFH group, the heparin infusion was adjusted to maintain the activated partial thromboplastin time (APTT) values below 70 s, or the post-filter activated clotting time (ACT) between 180 and 210 s in most studies [13, 26, 31–42]. In the RCA group, the post-filter ionized calcium concentration in most studies was kept at 0.25–0.35 mmol/L [32, 37, 43–45]. And the targeted systemic ionized calcium levels in most studies were kept at 0.9–1.2 mmol/L or 1.0–1.35 mmol/L [41, 44–47]. Thus, we consider the assumption of transitivity to be valid. In addition, there was no evidence of significant inconsistency in the network, either at the global or local levels (Additional File 5).

### Filter lifespan and filter clotting

**Fig. 2:**
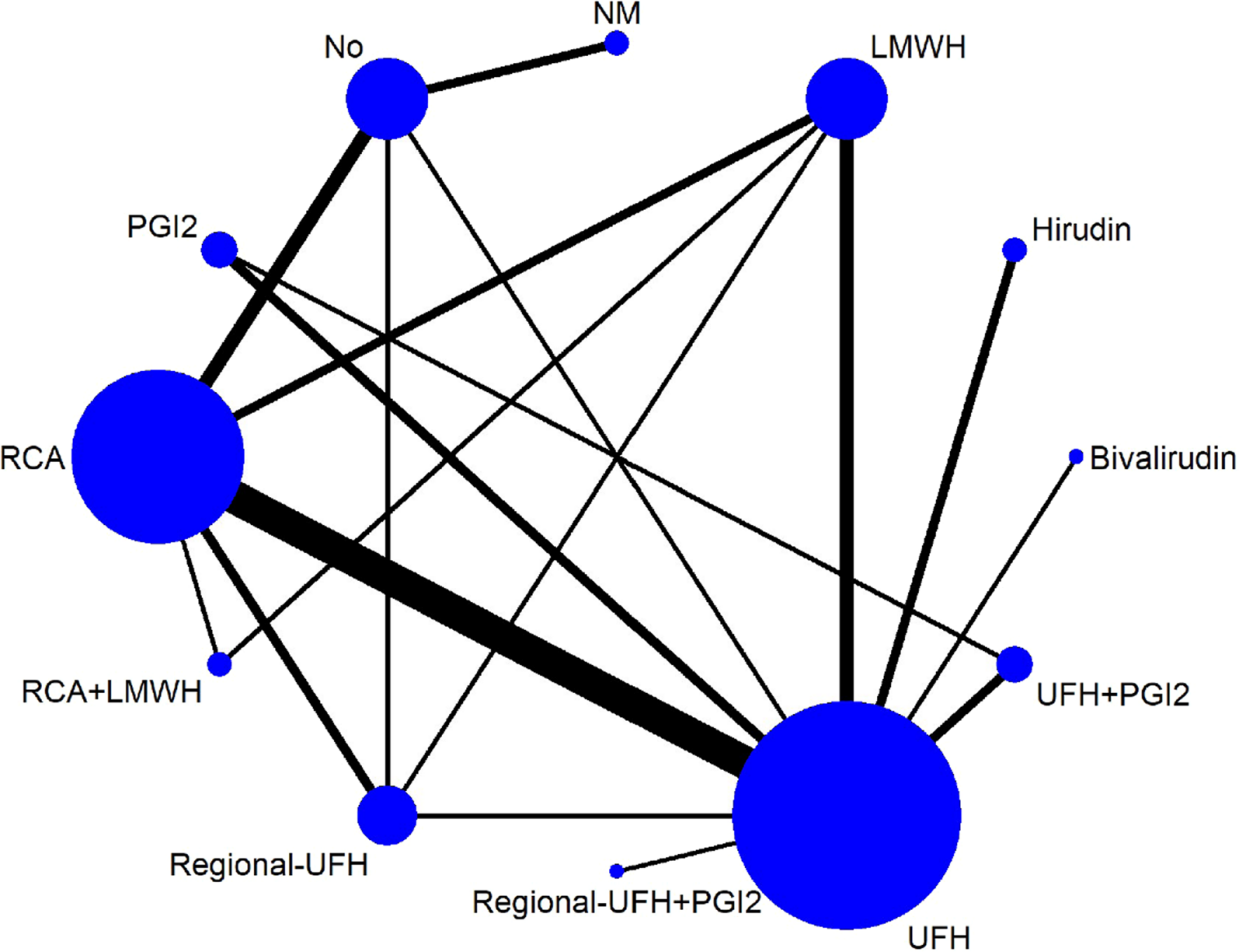
Network geometry of all the included anticoagulation options for evaluating filter lifespan. The size of the nodes and the thickness of the lines were proportional to the sample size and number of direct comparisons, respectively. Lines do not connect nodes when there were no head-to-head trials between two treatments. Abbreviations: UFH: unfractionated heparin; RCA: regional citrate anticoagulation; LMWH: low-molecular-weight heparin; PGI2: prostaglandin I2; NM: nafamostat mesilate; and No: no anticoagulation

**Fig. 3:**
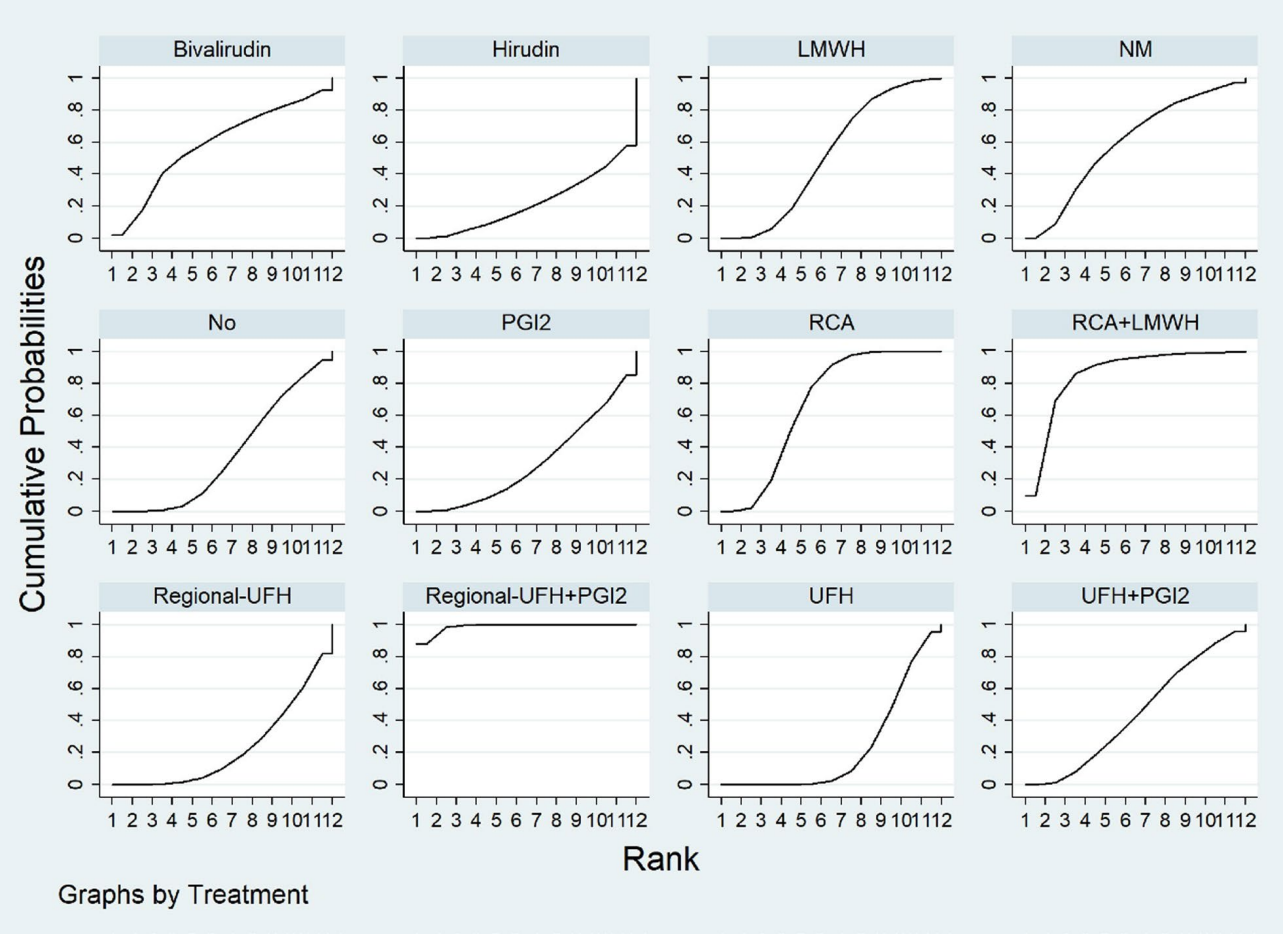
Filter lifespan ranking among different anticoagulation options

**Fig. 4:**
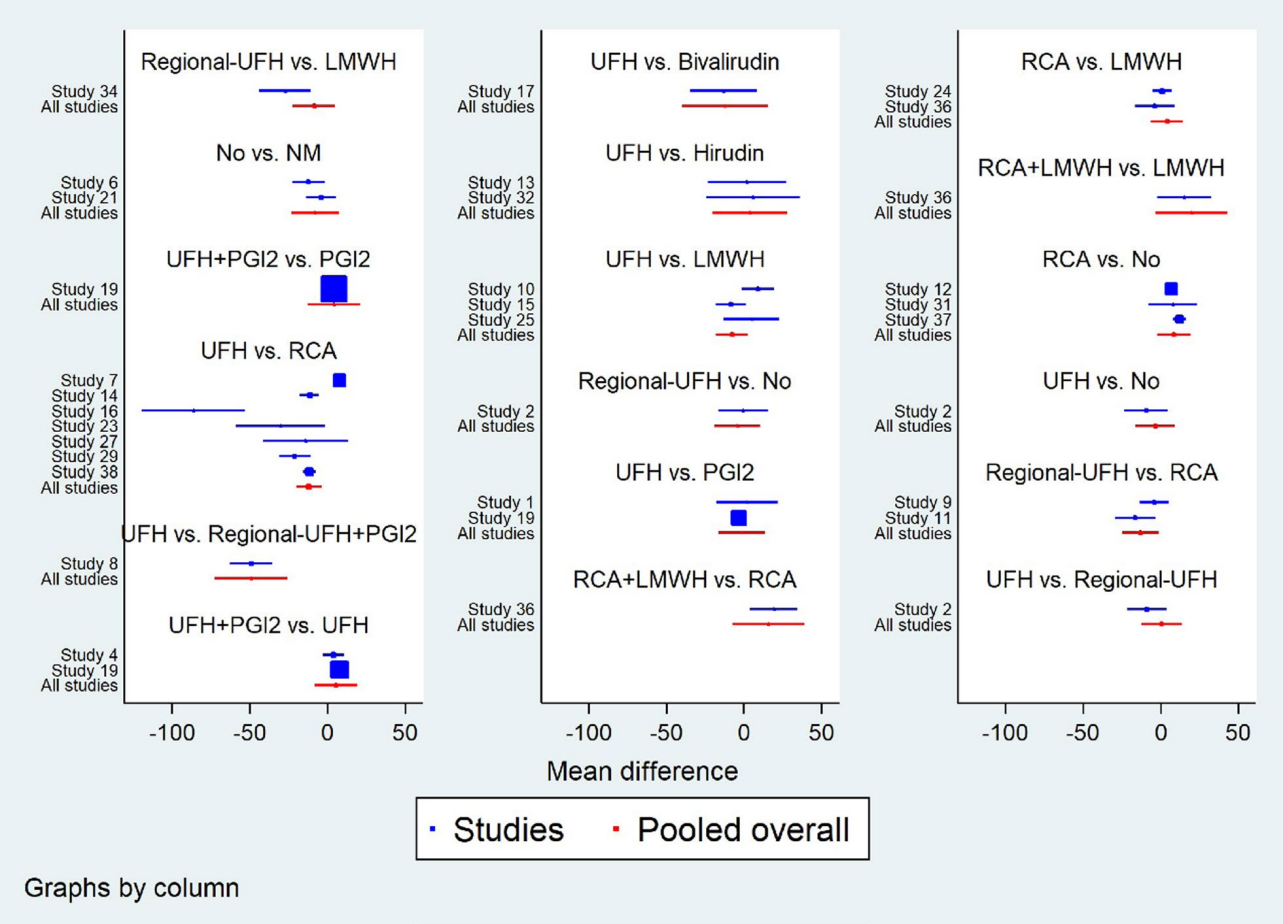
Forest plot in direct comparisons for evaluation of filter lifespan

Twenty-eight RCTs involving 2342 patients reported filter lifespan. A total of 12 anticoagulation options were included. The network geometry is shown in Fig. 2. No statistically significant heterogeneity was observed among the included RCTs within most direct pairwise comparisons except for three comparison pairs. Comparison pairs of LMWH versus UFH (*I*^2^ = 66.7%), RCA versus Regional-UFH plus protamine (*I*^2^ = 56.2%), and RCA versus no anticoagulation (*I*^2^ = 55.4%) showed moderate heterogeneity, but were not statistically significant (*p* > 0.10) (Additional File 3). The inconsistency test at the global and local levels also indicated no significant inconsistency (Additional File 5). Anticoagulation options ranking based on SUCRA values, which are shown in Fig. 3, from best to worst, were as follows: Regional-UFH + PGI2 98.7%, RCA + LMWH 85.5%, RCA 67.2%, NM 59.7%, Bivalirudin 58.8%, LMWH 51.9%, UFH + PGI2 44.4%, No 35.5%, PGI2 30.4%, UFH 23.1%, Regional-UFH 22.8%, and Hirudin 21.9%. The forest plot for direct comparison is shown in Fig. 4. Regional-UFH + PGI2 had the highest SCURA value, and Regional-UFH + PGI2 seemed to outperform RCA (MD 37.0, 95% CI 12.0 to 62.0), LMWH (MD 41.3, 95% CI 15.6 to 67.0), NM (MD 37.7, 95% CI 6.8 to 68.6), and other evaluated anticoagulation options in prolonging filter lifespan (Additional File 6: Fig. S1). However, only a single included RCT with 46 participants had evaluated Regional-UFH + PGI2. Therefore, additional high-quality studies are necessary before any recommendation. UFH and RCA are most frequently used to assess the filter lifespan, and there is strong evidence that RCA increases filter lifespan compared with UFH (MD 12.0, 95% CI 3.8 to 20.2) (Additional File 6: Fig. S1). Sensitivity analysis was also performed by excluding 6 trials with a blood flow rate > 200 ml/min or < 130 mL/min, or without details of blood flow rate. And compared to UFH (MD 18.0, 95% CI 0.0 to 36.0), RCA also showed superiority in prolonging filter lifespan (Additional File 6, Fig. S43).

Filter clotting is the most common cause of terminating hemofiltration, and 12 anticoagulation options were evaluated to decrease the rate of filter clotting (Additional File 6: Fig. S2). No statistically significant heterogeneity (*P* > 0.1) was observed, although comparison pairs of RCA versus UFH (*I*^2^ = 64.1%) showed moderate heterogeneity (Additional File 3). The SUCRA analysis and the forest plot are also limited in this analysis of Regional-UFH + PGI2 as it was carried out on data from a single trial with a small sample size. RCA (64.3%) ranked higher than LMWH (60.5%), UFH (34.3%), Regional-UFH (62.9%), NM (58.4%), and PGI2 (47.6%) (Additional File 6: Fig. S3). However, the forest plot for all comparisons indicated that RCA only had a lower incidence of filter clotting than UFH (Additional File 6: Fig. S5).

### All-cause mortality

A total of 24 RCTs with 1947 patients, which compared to 11 anticoagulation options for CRRT, were included in this NMA (Additional File 6: Fig. S6). There was no significant heterogeneity, or inconsistency among the included trials reporting all-cause mortality (Additional File 3; Additional File 5). RCA and UFH were the most frequently used anticoagulants to assess all-cause mortality; however, no significant difference was observed between the two anticoagulants in reducing all-cause mortality (RR 0.99, 95% CI 0.84 to 1.16) (Additional File 6: Fig. S9). Although based on SUCRA values, Bivalirudin as anticoagulation for CRRT had the highest SCURA value (76.0%) (Additional File 6: Fig. S7), its 95% CI was wide and contained the null effect when compared with the other 10 anticoagulation options (Additional File 6: Fig. S9). No significant difference was observed among the 11 anticoagulation options based on the forest plot for all comparisons. Therefore, the anticoagulation options ranking for all-cause mortality should be interpreted with caution.

### Length of hospital or ICU stay

Only a small subset of trials provided information on the length of hospital stay, with 4 RCTs [43, 44, 48, 49] involving 3 anticoagulation options. Therefore, NMA for this outcome was not performed. A total of 10 trials with 1123 patients reported data on length of ICU stay, and 7 anticoagulation options were assessed (Additional File 6: Fig. S10). Bivalirudin ranked the best according to SUCRA statistic (95.5%), followed by UFH (58.6%), Hirudin (47.6%), Regional-UFH (40.3%), RCA (38.0%), UFH + PGI2 (35.0%), and no anticoagulation (35.0%) (Additional File 6: Fig. S11). However, only a single study with a small sample size had evaluated Bivalirudin, and potential overinterpretation of the statistical testing and SUCRA ranking probably exist. Further high-quality RCTs are desirable to strengthen the effectiveness of Bivalirudin during CRRT. Moreover, no significant difference was observed among anticoagulation with UFH, Regional-UFH, RCA, UFH + PGI2, and Hirudin (Additional File 6: Fig. S13).

### Duration of CRRT

A total of 9 RCTs comparing 7 anticoagulation options reported data on duration of CRRT (Additional File 6: Fig. S14). The direct comparison between RCA and UFH was most frequently used to assess duration of CRRT. The anticoagulation options for CRRT ranking, based on SUCRA values, from largest to smallest, were as follows: Regional-UFH 73.0%, RCA 61.7%, LMWH 59.4%, Bivalirudin 48.8%, no anticoagulation 47.1%, UFH 37.9%, and NM 22.0% (Additional File 6: Fig. S15). However, according to the forest plot for all comparisons, Regional-UFH did not show an advantage in reducing the duration of CRRT compared to the other 6 anticoagulation options (Additional File 6: Fig. S17). Thus, the anticoagulation option ranking should be interpreted cautiously.

### Recovery of kidney function

A total of 10 trials with 1248 patients, which were compared to 8 anticoagulation options, reported recovery of kidney function (Additional File 6: Fig. S18). No heterogeneity was found among the included studies, and the consistent assumption was deemed acceptable. There is weak evidence suggesting that NM is beneficial to the recovery of kidney function compared to the other 7 anticoagulation options during CRRT. Although NM had the highest SCURA value (86.9%), its 95% CI was wide and contained the null effect when compared to other anticoagulation options (Additional File 6: Fig. S21). The reduction of Cr and BUN was also analyzed to reflect the recovery status of kidney function. With no difference in baseline characteristics between the control and intervention groups, we collected data on the levels of Cr and BUN after treatment to reflect the changes. Ten RCTs involving 7 anticoagulation options reported data on the reduction of Cr (Additional File 6: Fig. S22). Substantial heterogeneity was observed across studies within the comparison of PGI2 and UFH (*I*^2^ = 88.2%, P < 0.01), or RCA and no anticoagulation (*I*^2^ = 94.7%, P < 0.01). And according to the forest plot for all comparisons, no significant difference was observed among these 7 anticoagulation options (Additional File 6: Fig. S25). As for the analysis of the reduction of BUN, a significant heterogeneity among the included trials was also observed (Additional File 3), and none of the anticoagulation option showed an advantage over each other (Additional File 6: Fig. S29). Therefore, further high-quality RCTs are necessary to strengthen the evidence.

### Adverse events

A total of 13 RCTs comparing to 8 anticoagulation options contributed to the analysis of adverse events. However, two RCTs [50, 51] comparing NM and no anticoagulation were excluded from the analysis due to a lack of direct comparison with the other 6 anticoagulation options. Thus, only 11 RCTs comparing to 6 anticoagulation options were included in the NMA of adverse events (Additional File 6: Fig. S30). As for the direct comparison of NM and no anticoagulation, no statistically significant difference was observed (RR 1.07, 95% CI 0.75 to 1.51, P = 0.72). Unfractionated heparin plus Prostaglandin E1 (UFH + PGE1) ranked the best among the 6 anticoagulation options (Additional File 6: Fig. S31); however, there is weak evidence that it reduces the occurrence of adverse events (Additional File 6: Fig. S33). Compared to UFH, RCA had a higher SCURA value, and a lower occurrence of adverse events was observed according to the forest plot for all comparisons (Additional File 6: Fig. S33). We also analyzed bleeding events reported from 30 RCTs compared to 14 anticoagulation options (Additional File 6: Fig. S34). Although RCA + LMWH ranked highest for lower occurrence of bleeding events, its 95% CI was wide and contained the null effect when compared with other anticoagulation options. However, there is strong evidence that UFH (RR 3.09, 95% CI 2.08 to 4.60) and LMWH increase the risk of bleeding events compared with RCA (Additional File 6: Fig. S37). Metabolic disturbance was also analyzed in this NMA, with only 8 trials comparing 5 anticoagulation options (Additional File 6: Fig. S38). No statistically significant difference was observed between the 5 anticoagulation options (Additional File 6: Fig. S41). In summary, the most effective anticoagulation option for reducing the occurrence of adverse events should be interpreted cautiously. Anticoagulation with RCA during CRRT is superior to UFH in reducing adverse events, including bleeding events.

#### Costs

Cost-effectiveness is a critical consideration when selecting anticoagulants for CRRT in critically ill patients with AKI. However, only four RCTs [30, 41, 49, 52] have reported the data on the costs of RCA. Gao et al. [49] noted that the cost of CRRT per person was slightly lower in the RCA group than that in the no anticoagulation group. And Schilder et al. [41] indicated that the costs of the first 72 h of CRRT were lower in the RCA group, compared to the UFH group. Despite sodium citrate costing more per day than heparin, RCA was found to prolong filter lifespan, which resulted in an economic benefit. However, Fealy et al. [52] suggested that the magnitude of the gain in circuit life did not offset the additional cost of citrate sufficiently.

In addition, two studies have compared the total daily costs of LMWH and UFH. Joannidis et al. [36] observed a lower total daily cost of CRRT during anticoagulation with LMWH, which contradicts the finding in the study conducted by Reeves et al. [53].

#### Subgroup analyses

Two subgroup analyses were performed for populations with CVVH, CVVHD, or CVVHDF and pre-dilution or post-dilution, respectively. Overall, in the CVVH subgroup, no significant difference was observed between the evaluated anticoagulants, except for Regional-UFH + PGI2. However, only a single included RCT with 46 participants had evaluated Regional-UFH + PGI2, which should be interpreted with caution (Additional File 6: Fig. S44). In the CVVHDF subgroup, there was evidence that RCA is superior in prolonging filter lifespan compared with UFH. However, no significant difference was observed among other evaluated anticoagulants (Additional File 6: Fig. S45). Due to the lack of sufficient data, the network meta-model of CVVHD is not available. In the pre-dilution and post-dilution subgroups, compared to UFH, RCA and LMWH both showed superiority in prolonging filter lifespan (Additional File 6: Figs. S46, S47).

## Discussion

### Key findings

We performed an NMA of 37 RCTs comprising 2648 participants to evaluate the efficacy and safety of 14 different anticoagulation options during CRRT in adult critically ill patients. UFH and RCA are most frequently used to assess the outcomes. Our findings suggested that RCA has advantages not only in prolonging filter lifespan, but also in reducing bleeding events when compared to UFH. The SUCRA analysis and forest plot of Regional-UFH + PGI2 are limited as only a single study was included. Additional high-quality studies are necessary before any recommendation of Regional-UFH + PGI2. Moreover, there were similar effects in terms of length of ICU stay, all-cause mortality, duration of CRRT, recovery of kidney function, and adverse events among most evaluated anticoagulation options.

### Comparison with previous studies

Several systematic reviews and meta-analyses have compared the effects of RCA and other anticoagulants such as UFH during CRRT among critically ill patients. Six pairwise meta-analyses have compared the efficacy and safety of RCA and UFH in CRRT for critically ill patients [54–59]. A systematic review including 3 RCTs and 17 observational studies assessed the efficacy and safety of RCA, UFH, and NM compared to anticoagulation-free, respectively [60]. Another systematic review evaluated the effects of RCA versus UFH, Regional-UFH, and LMWH among critically ill patients treated with CRRT [61]. Our study comprehensively evaluated the effects of various anticoagulation options altogether, considering filter lifespan, all-cause mortality, length of stay, duration of CRRT, recovery of kidney function, and adverse events as outcomes. Unlike previous meta-analysis focused on comparisons of two anticoagulation options, our study was able to construct various anticoagulation options into a network structure, comprehensively evaluating the effectiveness of anticoagulants from both direct and indirect comparison. Comparison of our study with previous relevant studies is presented in Table 2. Such a comprehensive NMA would be more likely to accurately evaluate the efficacy and safety of different anticoagulation options during CRRT.

**Table 2:** Comparison of our study with previous relevant studies

Author	Type	Years of searching	Number of studies	RCTs	Observational	Anticoagulation options
This study	NMA	NA-2022	37 trials from 38 articles	37	–	14 different anticoagulation options
Zhang [42]	MA	1993–2017	20	3	17	RCA, UFH, NM, and no anticoagulation
Zhang [43]	MA	NA-2011	6	6	–	RCA, UFH, LMWH, and Regional-UFH
Chang [36]	MA	NA-2020	10	10	–	RCA and UFH
Wu [37]	MA	NA-2011	6	6	–	RCA and UFH
Bai [38]	MA	NA-2015	11	11	–	RCA and UFH
Tsujimoto [6]	MA	NA-2019	34	34	–	Several different anticoagulation options
Liu [39]	MA	NA-2015	14	14	–	RCA and UFH
Feng [40]	MA	NA-2019	16	16	–	RCA and UFH
Li [41]	MA	NA-2021	13	13	–	RCA and UFH

### Clinical implications and future studies

Although filter lifespan is influenced by various factors, such as the position and patency of the vascular access, catheter choice, and blood flow rate, the choice of anticoagulant is also an essential strategy to prolong filter lifespan during CRRT [62]. This NMA suggested that Regional-UFH + PGI2 seemed to outperform other evaluated anticoagulants in prolonging filter lifespan and decreasing the rate of filter clotting. However, only one RCT with 46 participants evaluated Regional-UFH + PGI2, and there was potential for attrition and reporting bias in this trial. Therefore, the SUCRA analysis and the forest plot of Regional-UFH + PGI2 have limitations. And additional high-quality studies are necessary before making any recommendation.

Our NMA revealed that RCA is more effective in prolonging filter lifespan than UFH during CRRT. Since the requirement for intravascular access and artificial circuits during CRRT may increase the risk of infection, the prolonged filter lifespan could be associated with an increased rate of infection. However, only a post hoc analysis of the RICH trial [63] had analyzed the relationship between prolonged filter lifespan and the risk of infection. Further investigation into the potential paradoxical and negative impact of prolonged filter lifespan and the risk of infection is needed to provide evidence for clinical practice. Another concern is the cost-effectiveness of RCA. Currently, there are still no firm conclusions about whether RCA has superior cost-effectiveness. Only four RCTs evaluated cost-effectiveness differences between RCA and other anticoagulation options. Future studies should therefore pay more attention to cost-effectiveness.

It should be noted that variations in RCA protocols do have an effect on filter outcomes. But unfortunately, none of the RCTs included in this NMA provided sufficient data to analyze the effects of variations in RCA protocols. Currently, a variety of RCA protocols involving mode of citrate delivery and different citrate-containing solutions have been described [64]. Our previous study indicated a simplified RCA-based CVVH protocol using calcium-containing replacement solution had a similar circuit lifespan compared to calcium-free replacement solution. However, we can continuously supplement calcium without the need for a separate intravenous catheter and the preparation of a large dose of intravenous calcium solution, making it more convenient to apply in RCA-CRRT practice [65]. Poh et al. [66] found that a low-dose RCA protocol (an initial citrate dose of 2.5 mmol/L instead of 3 mmol/L) had fewer citrate-related complications without loss of efficacy. However, there remain several reasons limiting the widespread use of RCA, including the scarcity of CRRT-specific citrate solutions and intravenous calcium, complexity of RCA protocols, and the increasing workload [64, 67]. Therefore, the development of better and simplified RCA protocols is crucial.

In our NMA, all-cause mortality was not significantly different among different anticoagulation options. Several meta-analyses had also indicated that anticoagulation with RCA or UFH during CRRT did not impact all-cause mortality [6, 57]. However, minimizing mortality is the ultimate goal of develop better adjuvant therapy. Therefore, further high-quality prospective controlled studies are urgently needed to investigate new anticoagulation options that may decrease mortality.

Our NMA did not find evidence supporting the superiority of any anticoagulant over another on recovery of kidney function. The reduction of Cr and BUN is a reflection of the recovery status of kidney function, and the recovery of kidney function ultimately benefits patient survival [68]. Thus, future studies should focus on the influence of recovery of kidney function. Adverse events including bleeding events and metabolic disturbance were reported in our study. Our NMA showed that the occurrence of adverse events was not significantly different among most anticoagulation options during CRRT. The most effective anticoagulation option for reducing the occurrence of adverse events remains unclear. Instead, our finding suggested the superiority of RCA in critically ill patients in reducing adverse events compared with UFH. Although critically ill patients with acute or chronic liver failure were excluded in this NMA, a meta-analysis [69] and several observational studies [70, 71] had indicated that RCA is also safe in patients with liver failure or at high risk of bleeding. Thus, further high-quality RCTs are desirable to investigate the most effective anticoagulation option for reducing adverse events when performing CRRT.

### Strengths and limitations

To our knowledge, our study comprehensively evaluated the efficacy and safety of various anticoagulation options altogether for critically ill patients with AKI. Our NMA allows the comparison of multiple anticoagulation options simultaneously in a single analysis and improves the precision by combining direct and indirect estimates. The findings of our NMA would add evidence to the future choice of anticoagulant during CRRT for critically ill patients. Our search strategy was extensive and included all the relevant studies with no publication year restrictions. It included data from more than 2500 patients, 37 RCTs from 38 articles, and multiple countries, including Australia, Italy, Germany, France, China, and so on. To ensure homogeneity and improve transitivity, we set strict inclusion criteria that only adult critically ill patients with AKI could be included. And the target anticoagulation levels were comparable among included studies. A variety of outcomes such as filter lifespan, all-cause mortality, length of stay, during of CRRT, recovery of kidney function, and adverse events were analyzed in our NMA. In addition, we have performed sensitivity analysis to eliminate the potential influence of blood flow rate on the results. And in order to eliminate the potential influence of other major non-pharmacological interventions on filter lifespan, we conducted two subgroup analyses for populations with CVVH, CVVHD, or CVVHDF and pre-dilution or post-dilution, respectively. Furthermore, two independent investigators have thoroughly evaluated the methodological quality of this study.

Despite these strengths, this NMA still has some limitations. Firstly, only a single included RCT with a small sample size had evaluated Regional-UFH + PGI2, and there was potential for attrition and reporting bias in this trial. The SUCRA analysis and the forest plot of Regional-UFH + PGI2 will be limited. Secondly, due to the nature of the intervention and clinical problem, most included RCTs were not double-blinded or conducted as an open-label study. Although it might not influence the outcomes, there is still potential for bias. Thirdly, insufficient data were available to evaluate the efficacy and safety of different anticoagulation options on length of hospital stay, which is also an important outcome to strengthen the evidence on the best choice of anticoagulation options during CRRT. Fourthly, moderate or significant heterogeneity existed in some outcomes such as the reduction of Cr and BUN. This was a potential factor that might influenced the robustness of these results. The results of these outcomes should therefore be interpreted cautiously. Last, but not least, only published trials with selective databases were included in this NMA, and reporting bias could not be ruled out because not all trials reported filter lifespan or filter clotting, especially when filter lifespan was not the primary outcome. Regardless of these limitations, we minimized bias throughout the analysis by strict method identification, data selection, statistical analysis, sensitivity analysis, and subgroup analyses. These steps would strengthen the stability and accuracy of this NMA.

## Conclusions

Between the RCA and UFH groups, RCA is the priority anticoagulant in prolonging filter lifespan and reducing the risk of bleeding. Regional-UFH + PGI2 and Bivalirudin were evaluated by a single study each. Thus, additional high-quality studies are necessary before any recommendation of Regional-UFH + PGI2 and Bivalirudin. No statistically significant difference was observed in all-cause mortality, duration of CRRT, recovery of kidney function, and adverse events among most evaluated anticoagulation options.

## Supplementary Information

**Additional file 1**. File 1: Search strategy.

**Additional file 2**. Details of the risk of bias of included studies.

**Additional file 3**. Direct pairwise comparisons and heterogeneity of major outcomes.

**Additional file 4**. Inclusion criteria and exclusion criteria of participants in the includedrandomized controlled trials.

**Additional file 5**. Inconsistency assessment globally with design by treatmentinteraction model or locally with loop specific approach.

**Additional file 6**. Additional Figure S1 to Figure S47.

## Data Availability

All data generated or analyzed during this study are included in this article and its additional information files.

## Abbreviations

CRRT: Continuous renal replacement therapy
AKI: Acute kidney injury
RCTs: Randomized controlled trials
NMA: Network meta-analysis
NM: Nafamostat mesilate
ICU: Intensive care unit
UFH: Unfractionated heparin
RCA: Regional citrate anticoagulation
LMWH: Low-molecular-weight heparin
PGI2: Prostaglandin I2
No: No anticoagulation
RR: Risk ratio
MD: Mean difference
SUCRA: Surface under the cumulative ranking curve
Cr: Creatinine
BUN: Blood urea nitrogen
