# Supplementary material for: Anticoagulation options for continuous renal replacement therapy in critically ill patients: a systematic review and network meta-analysis of randomized controlled trials
Source: Crit Care. 2023 Jun 7;27:222. doi: 10.1186/s13054-023-04519-1 (PMC10249230; doi:10.1186/s13054-023-04519-1)
Supplement: Supplementary file 1 — Additional file 1. File 1: Search strategy. [file 13054_2023_4519_MOESM1_ESM.docx]

Search strategy terms

| PubMed, searched 31/10/2022 | |
| --- | --- |
| 1 | Renal replacement therapy [All Fields] OR Continuous venovenous hemofiltration [All Fields] OR CVVH [All Fields] OR Continuous venovenous hemodialysis [All Fields] OR CVVHD [All Fields] OR Continuous venovenous hemodiafiltration [All Fields] OR CVVHDF [All Fields] OR Slow continuous ultrafiltration [All Fields] OR SCUF [All Fields] |
| 2 | Anticoagulation [All Fields] OR Citrate [All Fields] OR Heparin [All Fields] OR UFH [All Fields] OR LMWH [All Fields] OR Dalteparin [All Fields] OR Nadroparin [All Fields] OR Enoxaparin [All Fields] OR Bivalirudin [All Fields] OR Prostacyclin [All Fields] OR Nafamostat [All Fields] OR Hirudin [All Fields] OR Iloprost [All Fields] OR Tirofiban [All Fields] |
| 3 | Combine #1 AND #2 |

| Embase, searched 31/10/2022 | |
| --- | --- |
| 1 | Renal replacement therapy [ti/ab] OR Continuous venovenous hemofiltration [ti/ab] OR CVVH [ti/ab] OR Continuous venovenous hemodialysis [ti/ab] OR CVVHD [ti/ab] OR Continuous venovenous hemodiafiltration [ti/ab] OR CVVHDF [ti/ab] OR Slow continuous ultrafiltration [ti/ab] OR SCUF [ti/ab] |
| 2 | Anticoagulation [ti/ab] OR Citrate [ti/ab] OR Heparin [ti/ab] OR UFH [ti/ab] OR LMWH [ti/ab] OR Dalteparin [ti/ab] OR Nadroparin [ti/ab] OR Enoxaparin [ti/ab] OR Bivalirudin [ti/ab] OR Prostacyclin [ti/ab] OR Nafamostat [ti/ab] OR Hirudin [ti/ab] OR Iloprost [ti/ab] OR Tirofiban [ti/ab] |
| 3 | Combine #1 AND #2 |

| Web of Science, searched 31/10/2022 | |
| --- | --- |
| 1 | TS = (Renal replacement therapy) OR TS = (Continuous venovenous hemofiltration) OR TS = (CVVH) OR TS = (Continuous venovenous hemodialysis) OR TS = (CVVHD) OR TS = (Continuous venovenous hemodiafiltration) OR TS = (CVVHDF) OR TS = (Slow continuous ultrafiltration) OR TS = (SCUF) |
| 2 | TS = (Anticoagulation) OR TS = (Citrate) OR TS = (Heparin) OR TS = (UFH) OR TS = (LMWH) OR TS = (Dalteparin) OR TS = (Nadroparin) OR TS = (Enoxaparin) OR TS = (Bivalirudin) OR TS = (Prostacyclin) OR TS = (Nafamostat) OR TS = (Hirudin) OR TS = (Iloprost) OR TS = (Tirofiban) |
| 3 | Combine #1 AND #2 |

| the Cochrane Library, searched 31/10/2022 | |
| --- | --- |
| 1 | Renal replacement therapy [ti/ab/kw] OR Continuous venovenous hemofiltration [ti/ab/kw] OR CVVH [ti/ab/kw] OR Continuous venovenous hemodialysis [ti/ab/kw] OR CVVHD [ti/ab/kw] OR Continuous venovenous hemodiafiltration [ti/ab/kw] OR CVVHDF [ti/ab/kw] OR Slow continuous ultrafiltration [ti/ab/kw] OR SCUF [ti/ab/kw] |
| 2 | Anticoagulation [ti/ab/kw] OR Citrate [ti/ab/kw] OR Heparin [ti/ab/kw] OR UFH [ti/ab/kw] OR LMWH [ti/ab/kw] OR Dalteparin [ti/ab/kw] OR Nadroparin [ti/ab/kw] OR Enoxaparin [ti/ab/kw] OR Bivalirudin [ti/ab/kw] OR Prostacyclin [ti/ab/kw] OR Nafamostat [ti/ab/kw] OR Hirudin [ti/ab/kw] OR Iloprost [ti/ab/kw] OR Tirofiban [ti/ab/kw] |
| 3 | Combine #1 AND #2 |
