# Supplementary material for: Anticoagulation options for continuous renal replacement therapy in critically ill patients: a systematic review and network meta-analysis of randomized controlled trials
Source: Crit Care. 2023 Jun 7;27:222. doi: 10.1186/s13054-023-04519-1 (PMC10249230; doi:10.1186/s13054-023-04519-1)
Supplement: Supplementary file 2 — Additional file 2. Details of the risk of bias of included studies. [file 13054_2023_4519_MOESM2_ESM.docx]

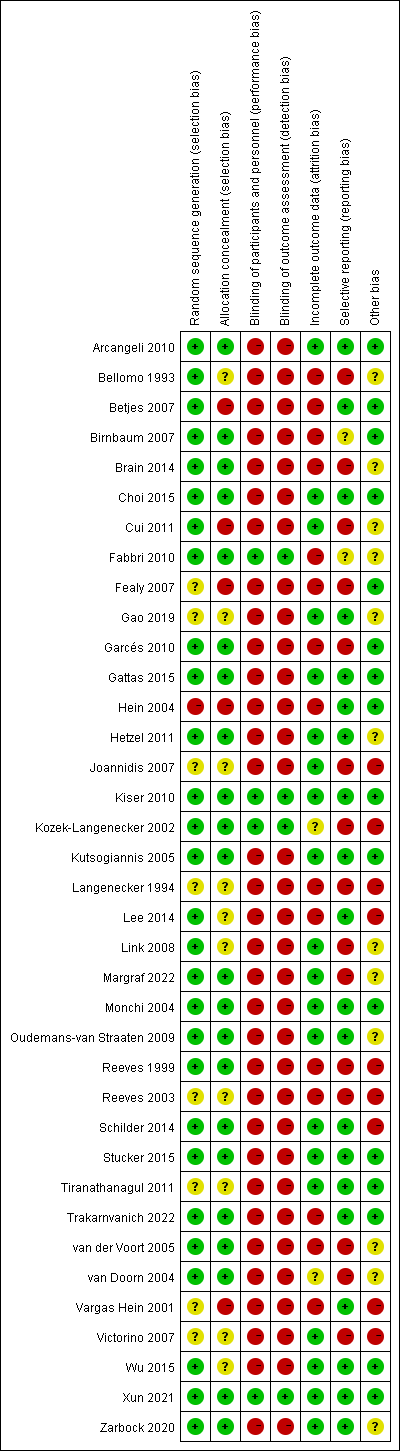


Figure 1. Potential risk of bias of each included randomized controlled trials.


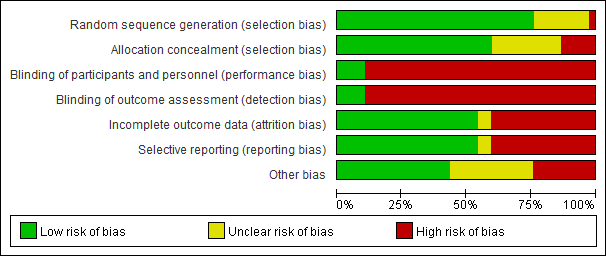


Figure 2. Summarized risk of the included randomized controlled trials.
