# Supplementary material for: Anticoagulation options for continuous renal replacement therapy in critically ill patients: a systematic review and network meta-analysis of randomized controlled trials
Source: Crit Care. 2023 Jun 7;27:222. doi: 10.1186/s13054-023-04519-1 (PMC10249230; doi:10.1186/s13054-023-04519-1)
Supplement: Supplementary file 3 — Additional file 3. Direct pairwise comparisons and heterogeneity of major outcomes. [file 13054_2023_4519_MOESM3_ESM.docx]

Table 1. Direct pairwise comparisons and heterogeneity of major outcomes.

| **Comparisons** | **Number of studies** | **MD/ RR (95% CI)** | **P** | **I^2^ /p value** |
| --- | --- | --- | --- | --- |
| 1. Filter lifespan | | | | |
| 1.1 UFH vs. | | | | |
| RCA | 7 | 17.75 (4.91, 30.59) | 0.01 | 45.4%, P=0.001 |
| LMWH | 3 | -1.38 (-13.42, 10.67) | 0.82 | 66.7%, P=0.145 |
| PGI2 | 2 | 3.45 (1.55, 5.35) | <0.01 | 0.0%, P=0.584 |
| UFH+PGI2 | 2 | 7.50 (5.86, 9.14) | <0.01 | 1.2%, P=0.314 |
| Hirudin | 2 | -3.66 (-23.04, 15.71) | 0.71 | 0.0%, P=0.842 |
| 1.2 Regional-UFH + protamine vs. | | | | |
| RCA | 2 | 8.32 (0.61, 15.86) | 0.03 | 56.2%, P=0.131 |
| 1.3 No anticoagulation vs. | | | | |
| RCA | 3 | 8.20 (6.15, 10.24) | <0.01 | 55.4%, P=0.106 |
| Nafamostat mesylate | 2 | 7.74 (0.70, 14.79) | 0.03 | 24.0%, P=0.251 |
| 1. Filter clotting | | | | |
| 2.1 UFH vs. |  |  |  |  |
| RCA | 6 | 0.69 (0.54, 0.89) | 0.01 | 64.1%, P=0.116 |
| 2.2 Regional-UFH + protamine vs. | | | | |
| RCA | 3 | 0.94 (0.83, 1.06) | 0.30 | 0.0%, P=0.801 |
| 1. All-cause mortality | | | | |
| 3.1 UFH vs. | | | | |
| RCA | 9 | 0.99 (0.96, 1.14) | 0.85 | 0.0%, P=0.935 |
| Hirudin | 2 | 1.02 (0.53, 1.99) | 0.95 | 0.0%, P=0.650 |
| 3.2 Regional-UFH + protamine vs. | | | | |
| RCA | 2 | 1.09 (0.73, 1.63) | 0.69 | 0.0%, P=0.797 |
| 3.3 LMWH vs. | | | | |
| RCA | 2 | 0.91 (0.68, 1.21) | 0.52 | 0.0%, P=0.409 |
| 3.4 No anticoagulation vs. | | | | |
| RCA | 4 | 0.75 (0.52, 1.09) | 0.13 | 0.0%, P=0.547 |
| Nafamostat mesylate | 2 | 0.96 (0.67, 1.34) | 0.80 | 14.1%, P=0.281 |
| 1. Length of ICU stay | | | | |
| 4.1 UFH vs. | | | | |
| RCA | 3 | 1.45 (-0.32, 3.22) | 0.11 | 0.0%, P=0.393 |
| Hirudin | 2 | 0.10 (-51.83, 52.83) | 0.99 | 0.0%, P=0.896 |
| 4.2 No anticoagulation vs. | | | | |
| RCA | 3 | -0.79 (-2.08, 0.51) | 0.23 | 0.0%, P=0.388 |
| 1. During of CRRT | | | | |
| 5.1 UFH vs. | | | | |
| RCA | 3 | 0.06 (-0.92, 1.05) | 0.90 | 0.0%, P=0.825 |
| 1. Recovery of renal function | | | | |
| 6.1 RCA vs. |  |  |  |  |
| UFH | 3 | 0.97 (0.79, 1.19) | 0.78 | 0.0%, P=0.950 |
| LMWH | 2 | 0.87 (0.65, 1.15) | 0.31 | 0.0%, P=0.392 |
| No anticoagulation | 2 | 0.74 (0.27, 2.03) | 0.56 | 64.0%, P=0.115 |
| 1. Reduction of Cr | | | | |
| 7.1 UFH vs. | | | | |
| RCA | 3 | -0.43 (-0.61, -0.26) | <0.01 | 0.0%, P=0.996 |
| PGI2 | 2 | -1.01 (-2.76, 0.75) | 0.26 | 88.2%, P=0.004 |
| UFH+PGI2 | 2 | -0.45 (-0.72, -0.18) | <0.01 | 0.0%, P=0.330 |
| 7.2 No anticoagulation vs. | | | | |
| RCA | 3 | -0.32 (-0.99, 0.36) | 0.36 | 94.7%, P<0.001 |
| 1. Reduction of BUN | | | | |
| 8.1 UFH vs. | | | | |
| RCA | 4 | 4.70 (-11.46, 20.86) | 0.57 | 84.1%, P<0.001 |
| PGI2 | 2 | -3.35 (-7.82, 1.12) | 0.14 | 44.3%, P=0.180 |
| UFH+PGI2 | 2 | 1.68 (-1.09, 4.46) | 0.24 | 0.0%, P=0.857 |
| 8.2 No anticoagulation vs. | | | | |
| RCA | 3 | -1.98 (-7.71, 3.74) | 0.50 | 90.9%, P<0.001 |
| 1. Adverse events | | | | |
| 9.1 RCA vs. | | | | |
| UFH | 6 | 1.79 (1.03, 3.13) | 0.04 | 53.9%, P=0.055 |
| Regional-UFH | 3 | 1.93 (0.72, 5.17) | 0.19 | 46.9%, P=0.152 |
| 9.2 No anticoagulation vs. | | | | |
| Nafamostat mesylate | 2 | 1.07 (0.75, 1.51) | 0.72 | 0.0%, P=0.391 |
| 1. Bleeding | | | | |
| 10.1 UFH vs. | | | | |
| RCA | 8 | 0.28 (0.18, 0.44) | <0.01 | 0.0%, P=0.786 |
| LMWH | 5 | 2.56 (0.65, 10.03) | 0.18 | 50.5%, P=0.109 |
| UFH+PGI2 | 3 | 0.21 (0.02, 1.78) | 0.15 | 0.0%, P=0.894 |
| Hirudin | 2 | 1.38 (0.29, 6.57) | 0.68 | 54.8%, P=0.137 |
| 10.2 Regional-UFH + protamine vs. | | | | |
| RCA | 3 | 0.34 (0.01, 8.32) | 0.51 | 0.0%, P<0.001 |
| 10.3 No anticoagulation vs. | | | | |
| RCA | 2 | 0.30 (0.07, 1.29) | 0.11 | 0.0%, P=0.825 |
| Nafamostat mesylate | 2 | 1.37 (0.45, 4.16) | 0.58 | 0.0%, P=0.418 |
| 1. Metabolic disturbance | | | | |
| 11.1 UFH vs. | | | | |
| RCA | 5 | 2.02 (1.08, 3.75) | 0.03 | 0.0%, P=0.499 |

UFH: unfractionated heparin; RCA: regional citrate anticoagulation; LMWH: low-molecular-weight heparin; PGI2: prostaglandin I2; CRRT: continuous renal replacement therapy; MD: mean difference; RR: risk ratio
