# Supplementary material for: Anticoagulation options for continuous renal replacement therapy in critically ill patients: a systematic review and network meta-analysis of randomized controlled trials
Source: Crit Care. 2023 Jun 7;27:222. doi: 10.1186/s13054-023-04519-1 (PMC10249230; doi:10.1186/s13054-023-04519-1)
Supplement: Supplementary file 4 — Additional file 4. Inclusion criteria and exclusion criteria of participants in the includedrandomized controlled trials. [file 13054_2023_4519_MOESM4_ESM.docx]

Table 2. Inclusion criteria and exclusion criteria of participants in the included randomized controlled trials.

| **Study** | **Inclusion criteria** | **Exclusion criteria** |
| --- | --- | --- |
| Arcangeli 2010 | Critically ill patients with AKI requiring CVVHDF, AKI was classified according to the RIFLE criteria | Therapy with aspirin or other NSAID in the previous 7 days; concomitant treatment with other extracorporeal organ-assist devices and any other drug affecting coagulation or platelets |
| Bellomo 1993 | Critically ill patients with AKI | Not reported |
| Betjes 2007 | Age >18 years and had been admitted to a combined medical and surgical ICU of the university hospital; the need for CRRT was based on clinical grounds by the consulting nephrologist | For UFH: heparin-induced thrombocytopenia, a platelet count < 20,000/mL, severe coagulopathy, a recent history (< month) of GI bleeding or intracranial hemorrhage or any other condition which constituted a high risk of bleeding as judged on clinical grounds by the treating physician  For citrate: severe circulatory shock, acute liver failure and an ionised plasma calcium concentration <0.8 mmol/L |
| Birnbaum 2007 | Critically ill patients with AKI. AKI was defined as urine output <500 mL/24h despite adequate fluid resuscitation or an increase in creatinine (normal <1.1 mg/dL) and urea (normal 14 to 46 mg/dL) to 3 times the normal values | Age <18 years; pregnancy; acute bleeding; hereditary coagulopathy; HIT II; platelet count < 30/nL; seizure disorder; and pre-existing chronic kidney failure. Break off criteria were a drop in platelet count (<30/nl), a clinically relevant drop in arterial oxygen tension, or bleeding |
| Brain 2014 | Adult patients > 18 years; diagnosis of acute renal failure with an indication for renal replacement therapy as assessed by one or more of the following criteria: 1) oliguria (urine output < 100 mL in a 6h period) unresponsive to fluid resuscitation, 2) volume overload, not correctable by diuretics in spite of adequate blood pressure and creatinine > 100 µmol/L, 3) increase of serum creatinine > 300 µmol/L or BUN >25 mmol/L, 4) increase of serum potassium > 6.5 mmol/L due to AKI; patients who at the time of inclusion had not yet started RRT | Patient weight <30 kg (machine specification for citrate dosing and ST100 membrane); inability to enter randomization due to a contraindication to one of the treatment arms: 1) indication for systemic anticoagulation with heparin (therapeutic range APTT) or an equivalent therapeutic dose of low-molecular weight heparin (this does not include routine thromboprophylaxis), 2) prior development of heparin-induced thrombocytopenia, 3) history of anaphylaxis to heparin, protamine or citrate; pregnancy or lactation; patients on CRRT before ICU presentation; indication for therapeutic hypothermia; previous participation in the same study; indication for a filter set other than the AN69 ST100 1m^2^ set or a specific dialysis prescription differing from the study protocol |
| Choi 2015 | Age >18 years and were admitted to ICU and required CRRT; active bleeding such as gastrointestinal bleeding and intracranial hemorrhage, activated partial thromboplastin time >60s, prothrombin time–international normalized ratio >2.0, thrombocytopenia (<100,000/μL), and surgery within 48h before CRRT | Pregnant or possibly pregnant women; allergic to nafamostat mesilate; hypercoagulable |
| Cui 2011 | Treated with continuous blood purification | Not reported |
| Fabbri 2010 | Critically ill patients between 18 and 85 years old with AKI requiring CVVH. AKI was defined as urine output <200 ml/12h associated with serum creatinine increase ≥133 mmol/L in 24h, despite adequate fluid resuscitation evaluated by echocardiography, and/or CVP values >10 mmHg | Acute or chronic liver failure; suspected ischemic hepatitis; cirrhosis; prolonged prothrombin and APTT; low platelet count, post-cardiac surgery; known sensitivity to heparin; known history of allergy |
| Fealy 2007 | AKI patients; RIFLE criteria "F", requiring CRRT and prescribed a regional anticoagulation method by the treating physician | Acute or chronic liver failure; suspected ischemic hepatitis; contraindication to heparin or protamine |
| Gao 2019 | Stage 3 adult AKI patients according to the KDIGO guideline who had high risk factors for bleeding including post-major surgeries involving the heart, chest, abdomen, spine or brain, coagulopathy (PT or APTT>1.5 times the normal control, or PT>18 s, APTT>60 s) and/or thrombocytopenia (<50×10^9^/L) induced by trauma, sepsis or active bleeding, and combined therapy with anticoagulant, antiplatelet or thrombolytic agents | Severe liver failure (serum total bilirubin >171 μmol/L) |
| Gattas 2015 | Critically ill adults fulfilling criteria: 1) AKI requiring CRRT,  2) suitability for regional anticoagulation of the CRRT circuit,  3) clinical equipoise regarding the method of circuit anticoagulation, and 4) informed consent was given or sought soon after enrolment | 1) expected stay in ICU less than 24h, 2) age <18 years, 3) pregnant or breastfeeding, 4) suspected ischemic hepatitis or liver failure, 5) known allergy to heparin or protamine, 6) suspected or confirmed HIT, and 7) chronic kidney disease requiring dialysis prior to ICU admission |
| Garcés 2010 | Adult medical or surgical patients admitted to a general ICU who presented AKI and were treated with CRRT | Age <18 years; chronic renal insufficiency prior to hospitalization in ICU; had already undergone dialysis; pregnancy; on chronic use of anticoagulants or had received any anticoagulant in the last 48h; immediate need for invasive interventions or surgery in the 24h following the prescription of CRRT; family refusal to sign the informed consent form |
| Hein 2004 | Critically ill patients with AKI and indication for CRRT. AKI was defined as a urine output <500 mL/24h despite adequate fluid resuscitation and/or an increase in creatinine (normal: < 115 mmol/L) and/or urea (normal: 2.3 to 7.6 mmol/L) of 2 times the normal values | Age <18 years, pregnancy, acute head injury, acute bleeding and HIT II |
| Hetzel 2011 | Written informed consent; >18 years; diagnosis of AKI and indication for KRT as assessed by one of the following criteria: (i) volume overload, not correctable by diuretics despite adequate blood pressure and SCr >1.2 mg/dL; (ii) increase of SCr >2.5 mg/ dL or BUN >50 mg/dL; and (iii) increase of serum potassium >5.5 mmol/L due to oligoanuria; patients who at the time of inclusion had not yet started with KRT; arterial line as vascular access; mechanical ventilation | HIT; need to continue effective systemic heparin anticoagulation with an APTT >20% above the upper limit of the normal range; metabolic alkalosis as defined by a pH >7.50 and base excess of >+4 mmol/L; pregnancy, lactation period; on chronic KRT; participation in another study during the preceding 3 months; previous participation in the same study |
| Joannidis 2007 | Adult surgical and medical ICU patients with an indication for CVVH. Indications for CVVH were AKI, persistent oliguria, severe acidosis, and septic shock with renal impairment | Intravenous use of heparin or enoxaparin within 12h before start of study; manifest bleeding or manifest clotting disorder as defined by PT (quick) <50%, PT-INR > 1.8, APTT > 45s, platelet count <50 × 10^9^/L; known hypersensitivity for heparins or expected/scheduled surgery or intervention requiring interruption of CVVH within the next 72h |
| Kiser 2010 | Age >18 years, admitted to an adult ICU, and diagnosed with AKI using the RIFLE criteria; undergone CRRT without anticoagulation during their hospitalization and experienced early (hemofilter survival time of ≤24h) hemofilter failure; receiving or were scheduled to receive additional CRRT for longer than 24h, and the renal medicine service had determined that anticoagulation was necessary for successful CRRT | Any contraindication to heparin or bivalirudin; ESKD requiring HD, pregnant, receiving activated protein C or prostacyclin therapy, or had another medical condition that required anticoagulation at therapeutic; active internal bleeding within 2 days or intracranial hemorrhage or stroke within 3 months of study enrolment; severe trauma with high risk of bleeding; platelet count < 30 × 10^3^/mm^3^, or coagulopathy (INR >2.5 or APTT >60s while not receiving anticoagulant therapy) as a result of liver failure or disseminated intravascular coagulation; prophylaxis of deep vein thrombosis with subcutaneous heparin or sequential compression devices was allowed at the discretion of the primary medical team |
| Kozek-Langenecker 2002 | Adults requiring CVVH for treatment of AKI secondary to sepsis or major surgery; patients were sedated, mechanically ventilated and additional therapy was administered according to the ICU's standard protocol; all patients received UFH as anticoagulant infused into the extracorporeal circuit before the hemofilter | pre-existing CKD; therapy with aspirin or other cyclooxygenase inhibitors; concomitant treatment with other extracorporeal organ assist devices |
| Kutsogiannis 2005 | Critically ill adults (>18 years) with AKI. AKI was defined as the standard RIFLE criteria | Contraindication to the use of systemic heparin or trisodium citrate or if they are anticipated to require systemic heparin for medical reasons; pregnant females |
| Langenecker 1994 | Critically ill patients with AKI | Not reported |
| Lee 2014 | Required CRRT and had at least one of the following hemorrhagic tendencies: (1) platelet count 100,000/mL, (2) APPT > 60s, (3) PT-INR > 2.0, (4) active hemorrhage, (5) surgery within the past 48 hours, (6) cerebral hemorrhage within the past 3 months or history of a major cerebral bleeding, and (7) septic shock or disseminated intravascular coagulation | Pregnant (or possibly pregnant) or breast feeding; allergic to nafamostat mesilate, or had any other conditions that made the candidate unfit according to the attending physician |
| Link 2008 | Clinical criteria: hypotension (SBP < 90 mmHg for at least 30 minutes or the need for supportive vasoactive medications to maintain SBP >90 mmHg) and evidence of end-organ hypoperfusion (cool, diaphoretic extremities). Hemodynamic criteria: a reduced cardiac index (<2.2 L/min/m2) and the presence of elevated pulmonary capillary occlusion pressure (>15 mm Hg); AKI with necessity for CRRT was defined as a urine output <0.5 mL/kg/hour for 6 hours and/or an increase in SCr ≥1.5 mg/dL within 24 hours according to the RIFLE criteria | Cardiopulmonary resuscitation; suspected concomitant sepsis defined by hemodynamic criteria (reduced systemic vascular resistance); platelet count <100 × 10^9^/L; major bleeding signs, APTT |
| Margraf 2022 | KDIGO stage 3 AKI classification, at least one additional condition (severe sepsis or septic shock, use of vasopressor, refractory fluid overload), age between 18 and 90 years, intention to provide full intensive care therapy for at least 3 days and written informed consent | Increased risk of bleeding, diseases with hemorrhagic diathesis, requirement of therapeutic anticoagulation, previous allergic reactions to one of the anticoagulants, known history of heparin-induced thrombocytopenia, severe lactic acidosis in the context of acute liver failure and/or shock, dialysis-dependent chronic kidney disease, permanent occlusion or surgical lesion of both kidney arteries as cause for AKI, (glomerulo-)nephritis as AKI cause, interstitial nephritis, vasculitis, urinary tract obstruction, kidney transplant within the last 12 months, hemolytic–uremic syndrome/thrombotic thrombocytopenic purpura, unavailability of machine for CVVH at inclusion, participation in another clinical intervention trial in last 3 months, any persons with dependency on the investigator, pregnancy or impending miscarriage |
| Monchi 2004 | AKI under CRRT required mechanical ventilation and had circulatory dysfunction with vasopressor dependency | Cirrhosis; severe coagulopathy; high risk of bleeding; known sensitivity to heparin |
| Oudemans-van Straaten 2009 | Adult critically ill patients with AKI requiring CRRT | Liver cirrhosis Child-Pugh C, (suspicion) of bleeding necessitating transfusion or fall in HB >0.5 mmol/L within 24h, surgery within 24h before CVVH, need of therapeutic anticoagulation, (suspected) HIT, chronic dialysis, and do-not-resuscitate orders |
| Reeves 1999 | Patients in the ICU requiring CVVH with filtration for AKI, or as adjunctive therapy in systemic inflammatory response syndrome | Pre-existing severe coagulopathy; disseminated intravascular coagulation; known sensitivity to heparin or dalteparin |
| Reeves 2003 | Requiring CRRT or AKI who had no contraindication to anticoagulation | Not reported |
| Schilder 2014 | Adults admitted to the ICUs of the participating centers, and who required CVVH. CVVH was started for AKI and uncontrolled uremia, diuretic-resistant volume overload, respiratory distress, multiorgan failure, or any combination of these features, at the discretion of the treating physician and consulting nephrologist | The presence of an increased bleeding risk (defined as a platelet count below 40 × 109/L, an activated partial thromboplastin time (aPTT) longer than 60 seconds, a prothrombin time-international normalised ratio (PT-INR) greater than 2.0 or recent major bleeding), age below 18 or over 80 years, the need for therapeutic systemic anticoagulation (heparin or coumarins) or a known HIT, the administration of activated protein C or plasma exchange therapy |
| Stucker 2015 | ICU patients were eligible if they were ≥18 years of age and had an AKI requiring CRRT according to the kidney-failure criteria of the RIFLE definition | Patients had active hemorrhagic disorders or severe thrombocytopenia (<50 × 109/L), a history of heparin-induced thrombocytopenia, severe liver failure defined as a factor V<20%, or were on the waiting list for liver transplantation |
| Tiranathanagul 2011 | Critically ill patients with AKI who underwent CVVH, AKI was defined by the RIFLE criteria | Contraindications for heparin use; receiving anticoagulant with other indications; previous dialysis in 24h; hypercalcemia (>3 mmol/L), severe hepatitis (AST or ALT >1000 IU/L) and cirrhosis |
| Trakarnvanich 2022 | Adult critically ill patients with stage 3 AKI (defned by  KDIGO 2012 criteria); requirement for CRRT, age older than  18 years, and no contraindication to CRRT | baseline serum creatinine >2mg/dL (male) or >1.5mg/dL (female), a history of renal transplantation, known pregnancy, a previous dialysis within 30 days, severe liver disease, end-stage heart disease or untreatable malignancy, a moribund status with expected survival less than 30 days, previous use of heparin or other anticoagulant, antiplatelet therapy within 7 days except for deep vein thrombosis, active bleeding at the time of enrollment and/or severe coagulopathy, receiving blood or blood components before enrollment, a hemoglobin level less than 7.5 g/dL and/or platelet count less than 100,000/mm^3^, previous underlying clotting disorders such as a hypercoagulable state, severe malnutrition (body mass index less than 18), and CRRT for reasons other than acute AKI |
| Vargas Hein 2001 | Critically ill patients with AKI and indication for CRRT. AKI was defined as a urine output <500 mL/24h despite adequate fluid resuscitation and/or an increase in creatinine (normal: < 115 mmol/L) and/or urea (normal: 2.3 to 7.6 mmol/L) of 2 times the normal values | Age <18 years, pregnancy, acute head injury, acute bleeding and HIT II |
| van Doorn 2004 | Critically ill patients with AKI | Not reported |
| van der Voort 2005 | Critically ill patients with AKI | Not reported |
| Victorino 2007 | AKI and respiratory failure | Not reported |
| Wu 2015 | Requirement of CVVH for an expected duration of >48h; no contraindication for LMWH systemic anticoagulation; written consent obtained from the patient or next of kin | Age ≤18 years; preexisting coagulopathy (defined as international normalization ratio >1.8 or prothrombin time >50% above the upper limit of normal values or platelet count <50 × 10^9^/l) or liver failure (defined as aspartate aminotransferase >500U/l or total bilirubinan >200μmol/l); any anticoagulation/hemostatic agent within 24h prior to enrollment; requiring any anticoagulation/hemostatic agent for reasons other than CRRT after enrollment |
| Xun 2021 | ≥ 65 years old; high-risk of spontaneous bleeding: heparin-related bleeding, PT> 18 s, APTT> 45 s, INR> 1.5, thrombocytopenia caused by anticoagulants, anti-platelets or thrombolytic agents combination therapy (Platelets < 50 × 10 ^9^/L); within the time frame of 48 h post-surgery; receiving CRRT | Presence of contraindications to CRRT; severe hypotension and hypoxemia cannot be corrected; abnormal coagulation function caused by diseases of the blood system; severe abnormal liver function; malignant tumour complication; sodium citrate allergies; pregnant or lactating women |
| Zarbock 2020 | (1) KDIGO stage 3 AKI classification (urine output <0.3 mL/kg/h for ≥24h, and/or >3-fold increase in serum creatinine level compared with baseline, and/or serum creatinine level of ≥4 mg/dL [353.6 μmol/L] with an acute increase of at least 0.5 mg/dL [44.2 μmol/L] within 48h) or an absolute indication for continuous kidney replacement therapy (serum urea levels >150 mg/dL, serum potassium levels >6 mmol/L, serum magnesium levels >9.7 mg/dL [4 mmol/L], blood pH <7.15, urine production <200 mL/12h or anuria, or fluid overload with edema in the presence of acute kidney injury resistant to diuretic treatment); (2) at least 1 additional condition (severe sepsis or septic shock, use of vasopressor, refractory fluid overload); (3) age between 18 and 90 years; (4) intention to provide full intensive care treatment for at least 3 days; and (5) provision of written informed consent | An increased bleeding risk, had diseases with hemorrhagic diathesis, needed therapeutic anticoagulation, had previous allergic reactions to one of the anticoagulants, had known HIT, or had persistent and severe lactic acidosis (pH <7.2 in 2 consecutive measurements for >2h and lactate level >72.1 mg/dL [8 mmol/L]) in the context of acute liver failure, shock, or both. Patients with severe lactic acidosis in the context of liver failure or shock. Additional exclusion criteria included dialysis-dependent chronic kidney disease; AKI caused by permanent occlusion or surgical lesion of both kidney arteries; AKI caused by glomerulonephritis, interstitial nephritis, vasculitis, or urinary tract obstruction; kidney transplant within the last 12 months; hemolytic-uremic syndrome/thrombotic thrombocytopenic purpura; no machine for continuous kidney replacement therapy available at the moment of inclusion; participation in another clinical intervention trial in the last 3 months; any kind of dependency on the investigator or employed by the sponsor or investigator; pregnancy and nursing period; and impending miscarriage |
