# Supplementary material for: Anticoagulation options for continuous renal replacement therapy in critically ill patients: a systematic review and network meta-analysis of randomized controlled trials
Source: Crit Care. 2023 Jun 7;27:222. doi: 10.1186/s13054-023-04519-1 (PMC10249230; doi:10.1186/s13054-023-04519-1)
Supplement: Supplementary file 5 — Additional file 5. Inconsistency assessment globally with design by treatmentinteraction model or locally with loop specific approach. [file 13054_2023_4519_MOESM5_ESM.docx]

Table 1. Inconsistency assessment globally with design-by-treatment interaction model.

|  |  | Filter lifespan | Filter clotting | All-cause mortality | Length of ICU stay | RRT during |
| --- | --- | --- | --- | --- | --- | --- |
| Entire network (design by treatment) Inconsistency | χ2 | 4.55 | 1.68 | 4.96 | 0.81 | 1.32 |
|  | p-value* | 0.71 | 0.43 | 0.29 | 0.67 | 0.25 |

|  |  | Reduction of Cr | Reduction of BUN | Renal function recovery | Bleeding | Metabolic disorder |
| --- | --- | --- | --- | --- | --- | --- |
| Entire network (design by treatment) Inconsistency | χ2 | 8.34 | 1.57 | 1.05 | 2.12 | 2.98 |
|  | p-value* | 0.08 | 0.81 | 0.30 | 0.71 | 0.08 |

*If the p-value is less than 0.05, the null hypothesis (no inconsistency) is rejected.

Table 2. Inconsistency assessment locally with loop-specific approach.

| **Loop** | **IF** | **P** | **95%CI** | **Loop_Heterog_tau2** |
| --- | --- | --- | --- | --- |
| 1. Filter lifespan | | | | |
| RCA-UFH-LMWH | 20.184 | 0.296 | (0.00, 58.06) | 200.799 |
| RCA-UFH-No | 2.680 | 0.911 | (0.00, 49.58) | 143.252 |
| RCA-UFH-Regional-UFH | 1.444 | 0.960 | (0.00, 57.46) | 218.857 |
| RCA -Regional-UFH-No | 0.925 | 0.928 | (0.00, 20.89) | 10.204 |
| RCA-Regional-UFH-LMWH | 18.073 | 0.100 | (0.00, 39.63) | 10.928 |
| RCA-LMWH-RCA+LMWH | 4.900 | 0.693 | (0.00, 29.20) | 0.000 |
| UFH-PGI2-UFH+PGI2 | 3.648 | 0.330 | (0.00, 10.99) | 0.000 |
| 1. Filter clotting | | | | |
| RCA-UFH-LMWH | 0.998 | 0.355 | (0.00, 3.11) | 0.372 |
| UFH-UFH+PGE1-UFH+PGI2 | 0.666 | 0.312 | (0.00, 1.96) | 0.000 |
| 1. All-cause mortality | | | | |
| RCA-UFH-No | 1.405 | 0.147 | (0.00, 3.30) | 0.000 |
| RCA-UFH-LMWH | 0.055 | 0.830 | (0.00, 0.55) | 0.000 |
| RCA-LMWH-RCA+LMWH | 0.631 | 0.830 | (0.00, 1.94) | 0.000 |
| 1. Length of ICU stay | | | | |
| RCA-UFH-No | 16.396 | 0.416 | (0.00, 55.95) | 1.123 |
| 1. Duration of CRRT |  |  |  |  |
| RCA-UFH-LMWH | 0.895 | 0.251 | (0.00, 2.42) | 0.000 |
| 1. Recovery of renal function |  |  |  |  |
| RCA-LMWH-RCA+LMWH | 1.138 | 0.346 | (0.00, 3.50) | 0.000 |
| 1. Reduction of Cr | | | | |
| RCA-UFH-No | 0.850 | 0.476 | (0.00, 3.19) | 0.185 |
| 1. Reduction of BUN | | | | |
| RCA-UFH-No | 3.565 | 0.959 | (0.00, 138.58) | 15.328 |
| UFH-PGI2-UFH+PGI2 | 13.218 | 0.182 | (0.00, 32.63) | 0.000 |
| 1. Bleeding | | | | |
| RCA-UFH-LMWH | 0.993 | 0.154 | (0.00, 2.36) | 0.086 |
| UFH-UFH+PGE1-UFH+PGI2 | 0.955 | 0.667 | (0.00, 5.30) | 0.000 |
| RCA-LWMH-RCA+LMWH | 0.464 | 0.779 | (0.00, 3.71) | 0.000 |
| UFH-PGI2-UFH+PGI2 | 2.143 | 0.893 | (0.00, 4.49) | 0.000 |
