# Supplementary material for: Anticoagulation options for continuous renal replacement therapy in critically ill patients: a systematic review and network meta-analysis of randomized controlled trials
Source: Crit Care. 2023 Jun 7;27:222. doi: 10.1186/s13054-023-04519-1 (PMC10249230; doi:10.1186/s13054-023-04519-1)
Supplement: Supplementary file 6 — Additional file 6. Additional Figure S1 to Figure S47. [file 13054_2023_4519_MOESM6_ESM.docx]

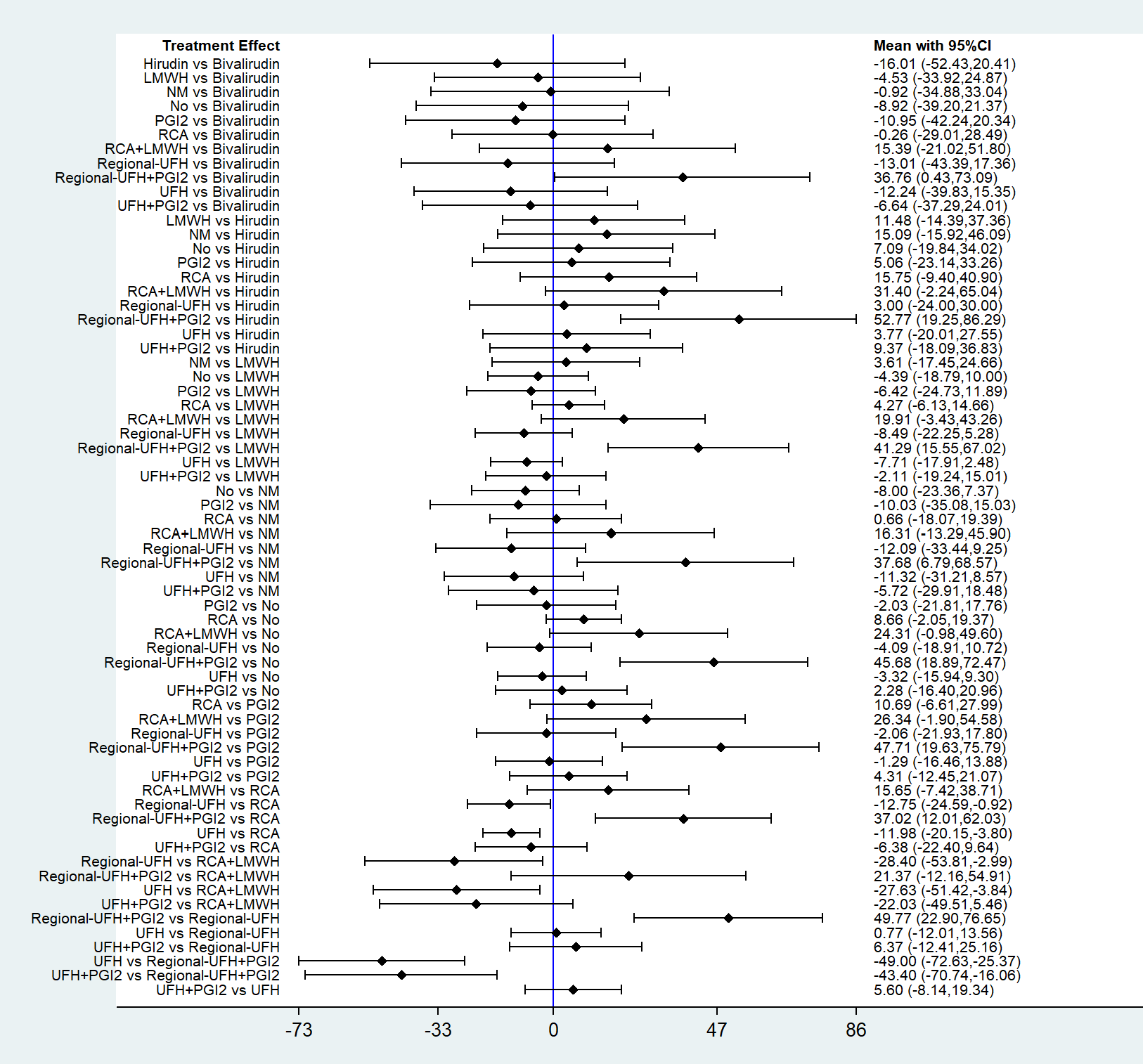


Figure S1. Forest plot of network meta-analysis for filter lifespan.


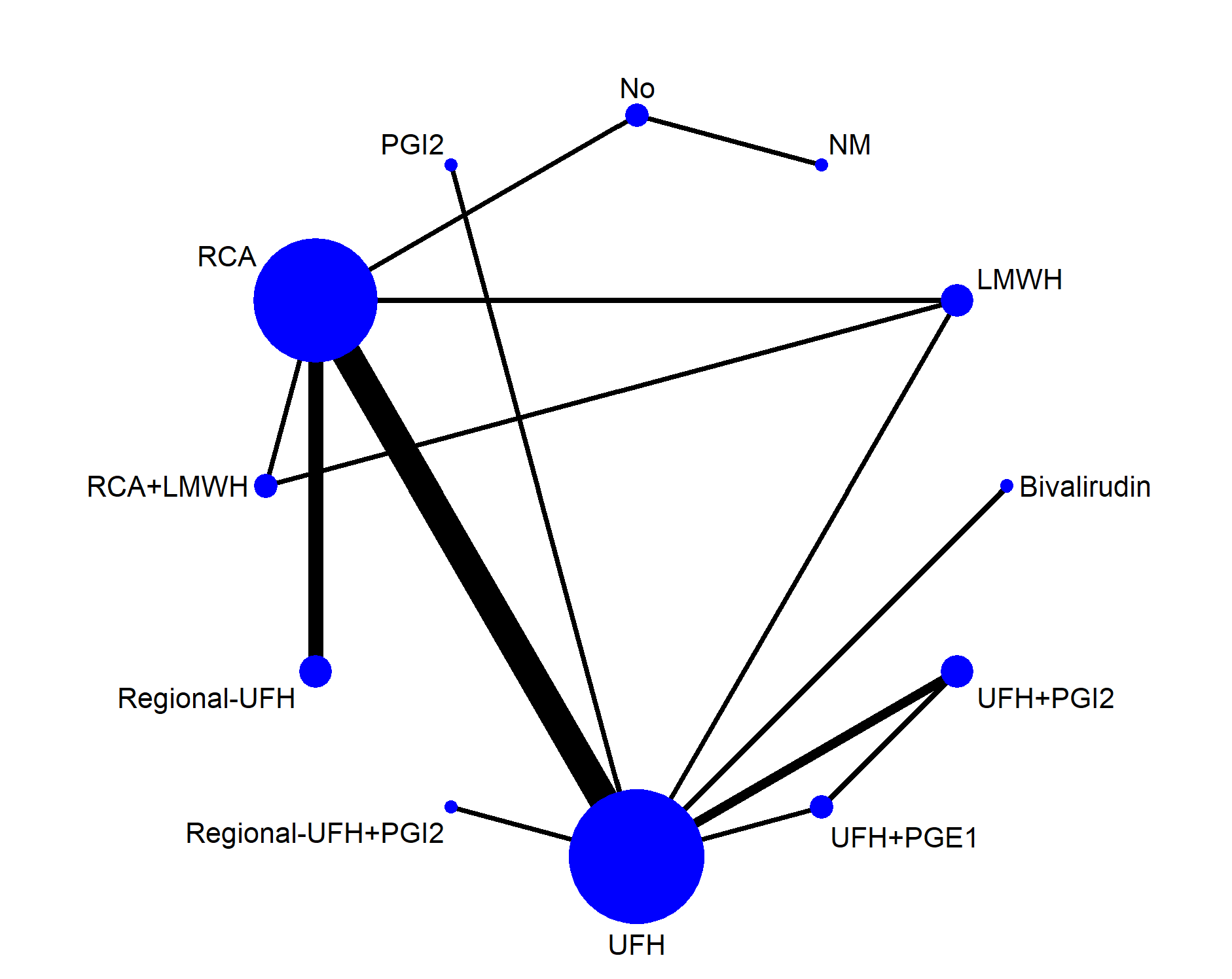


Figure S2. Network geometry of all included anticoagulation options for evaluating filter clotting.


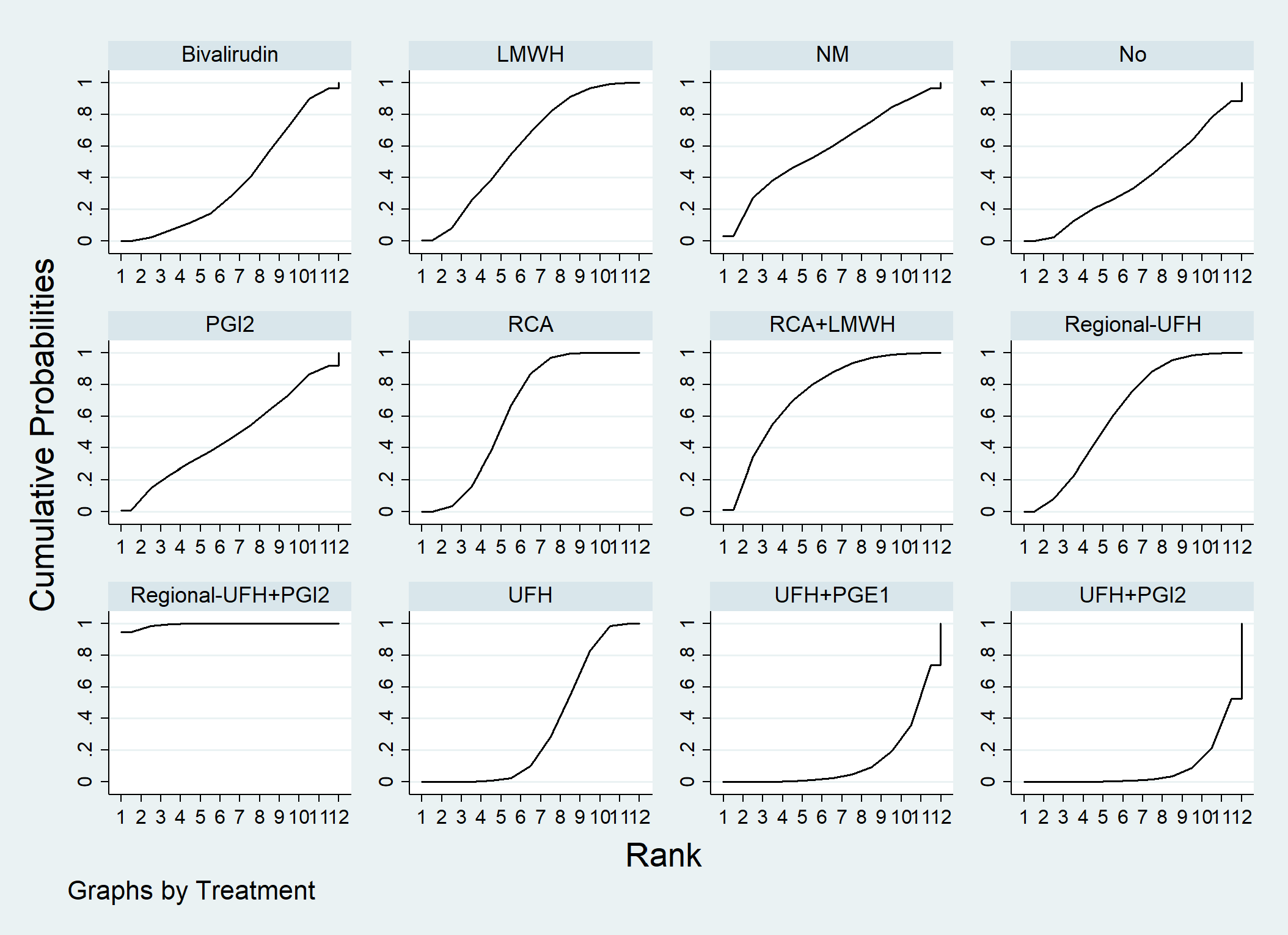


Figure S3. Filter clotting ranking among different anticoagulation options.


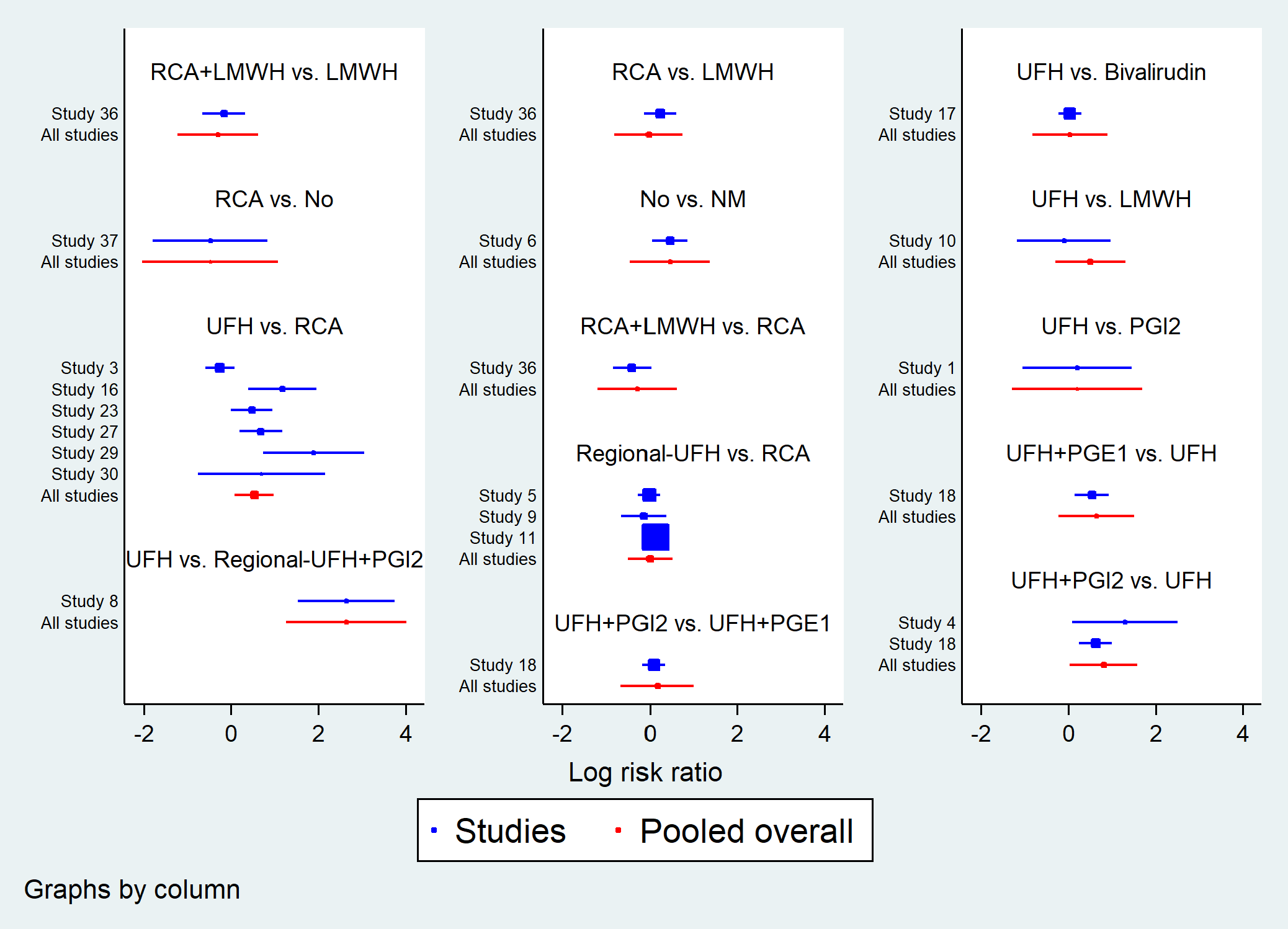


Figure S4. Forest plot in direct comparisons for evaluation of filter clotting. Study 1: Arcangeli 2010, study 2: Bellomo 1993, study 3: Betjes 2007, study 4: Birnbaum 2007, study 5: Brain 2014, study 6: Choi 2015, study 7: Cui 2011, study 8: Fabbri 2010, study 9: Fealy 2007, study 10: Garcés 2010, study 11: Gattas 2015, study 12: Gao 2019, study 13: Hein 2004, study 14: Hetzel 2011, study 15: Joannidis 2007, study 16: Kutsogiannis 2005, study 17: Kiser 2010, study 18: Kozek-Langenecker 2002, study 19: Langenecker 1994, study 20: Link 2008, study 21: Lee 2014, study 22: Margraf 2022, study 23: Monchi 2004, study 24: Oudemans-van Straaten 2009, study 25: Reeves 1999, study 26: Reeves 2003, study 27: Schilder 2014a, study 28: Schilder 2014b, study 29: Stucker 2015, study 30: Tiranathanagul 2011, study 31: Trakarnvanich 2022, study 32: Vargas Hein 2001, study 33: van Doorn 2004, study 34: van der Voort 2005, study 35: Victorino 2007, study 36: Wu 2015, study 37: Xun 2021, study 38: Zarbock 2020.


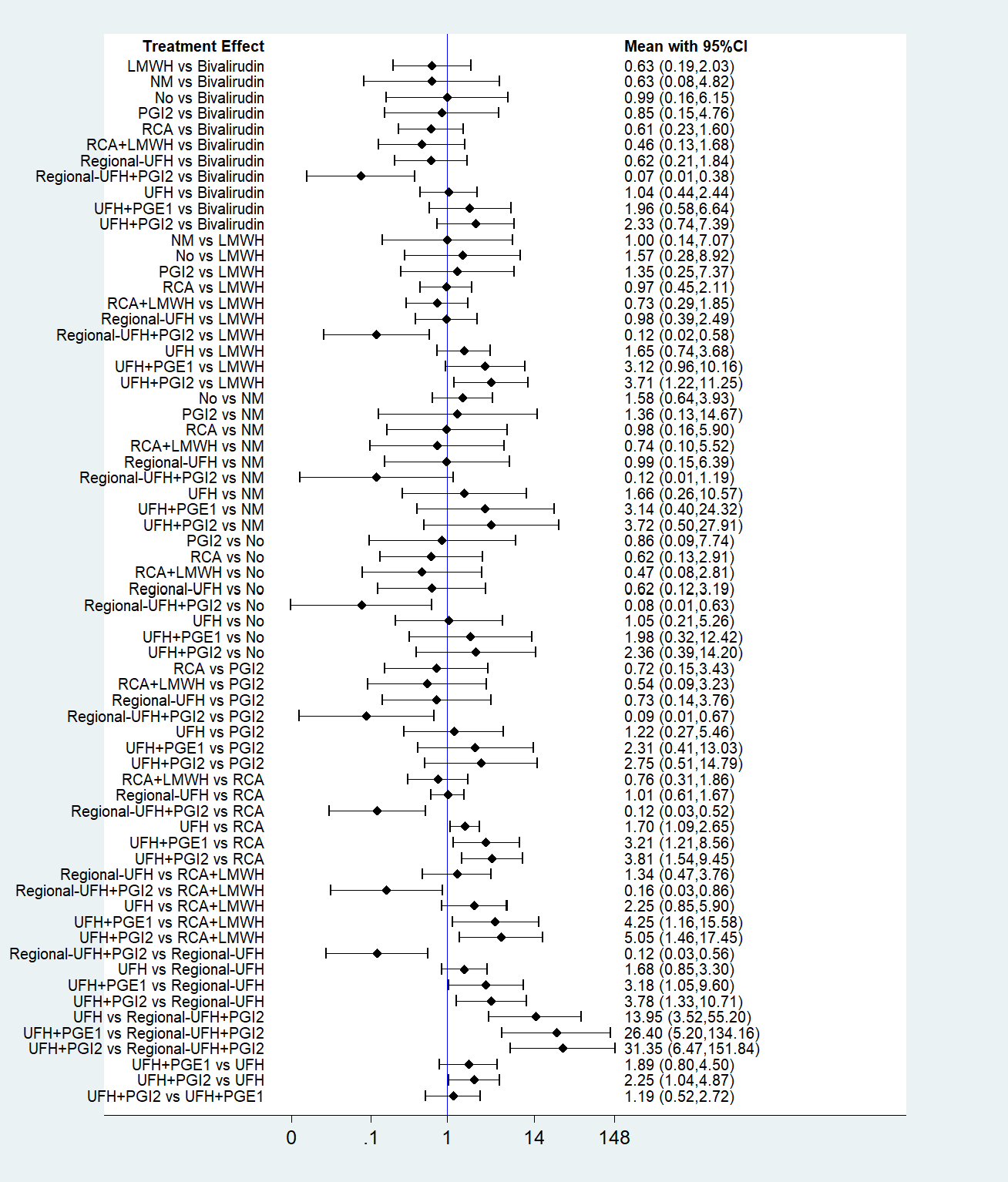


Figure S5. Forest plot of network meta-analysis for filter clotting.


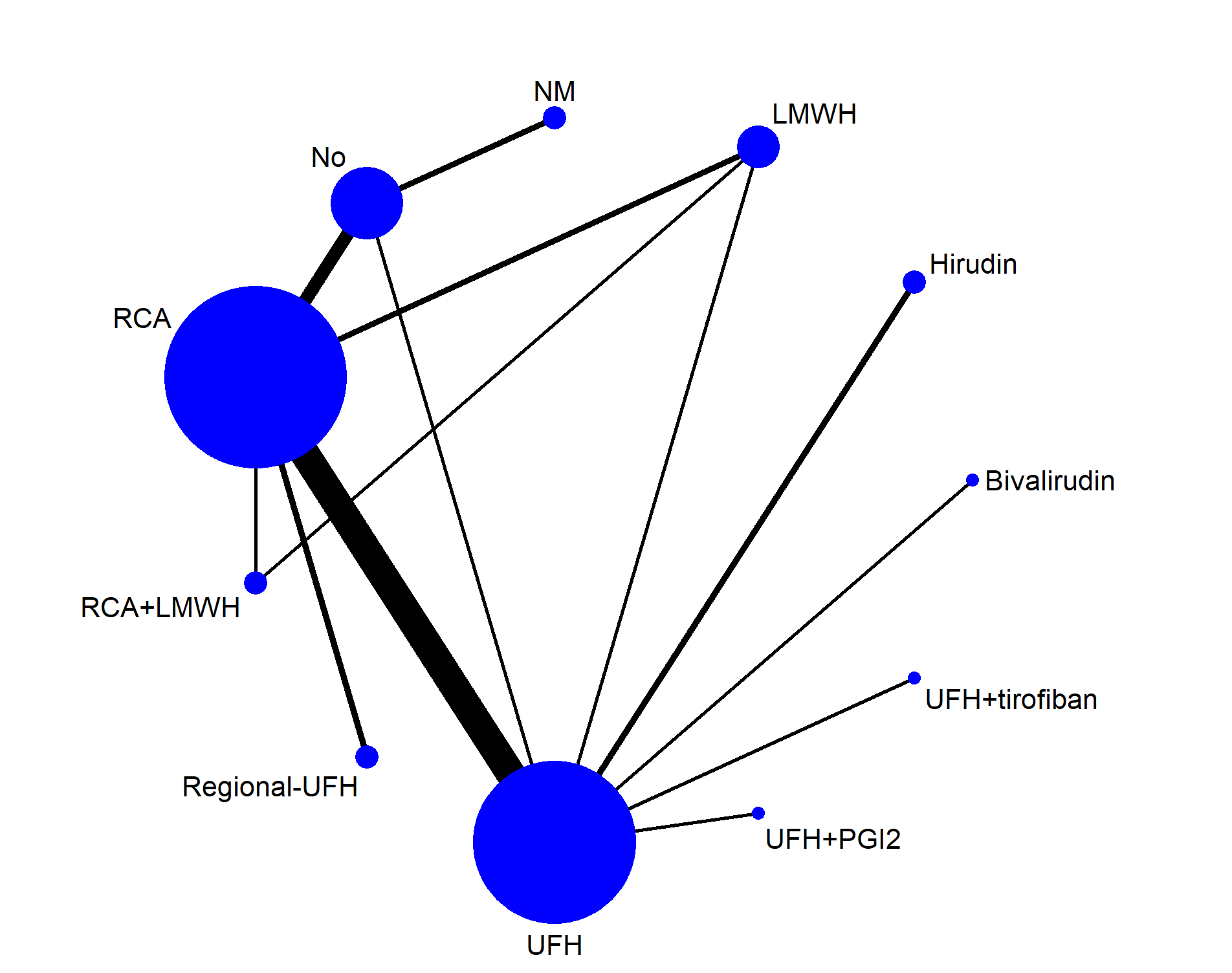


Figure S6. Network geometry of all the included anticoagulation options for evaluating all-cause mortality.


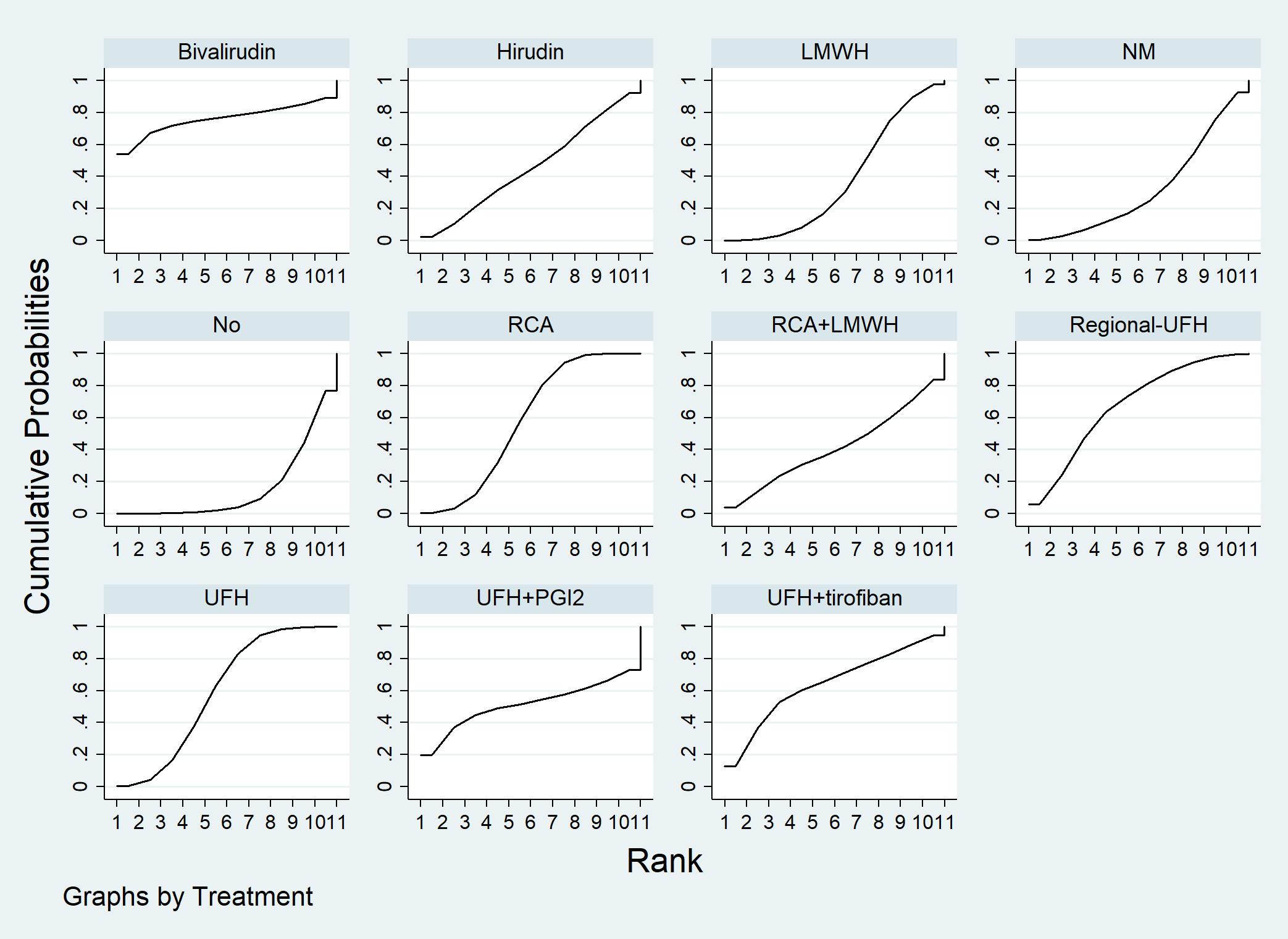


Figure S7. All-cause mortality ranking among different anticoagulation options.


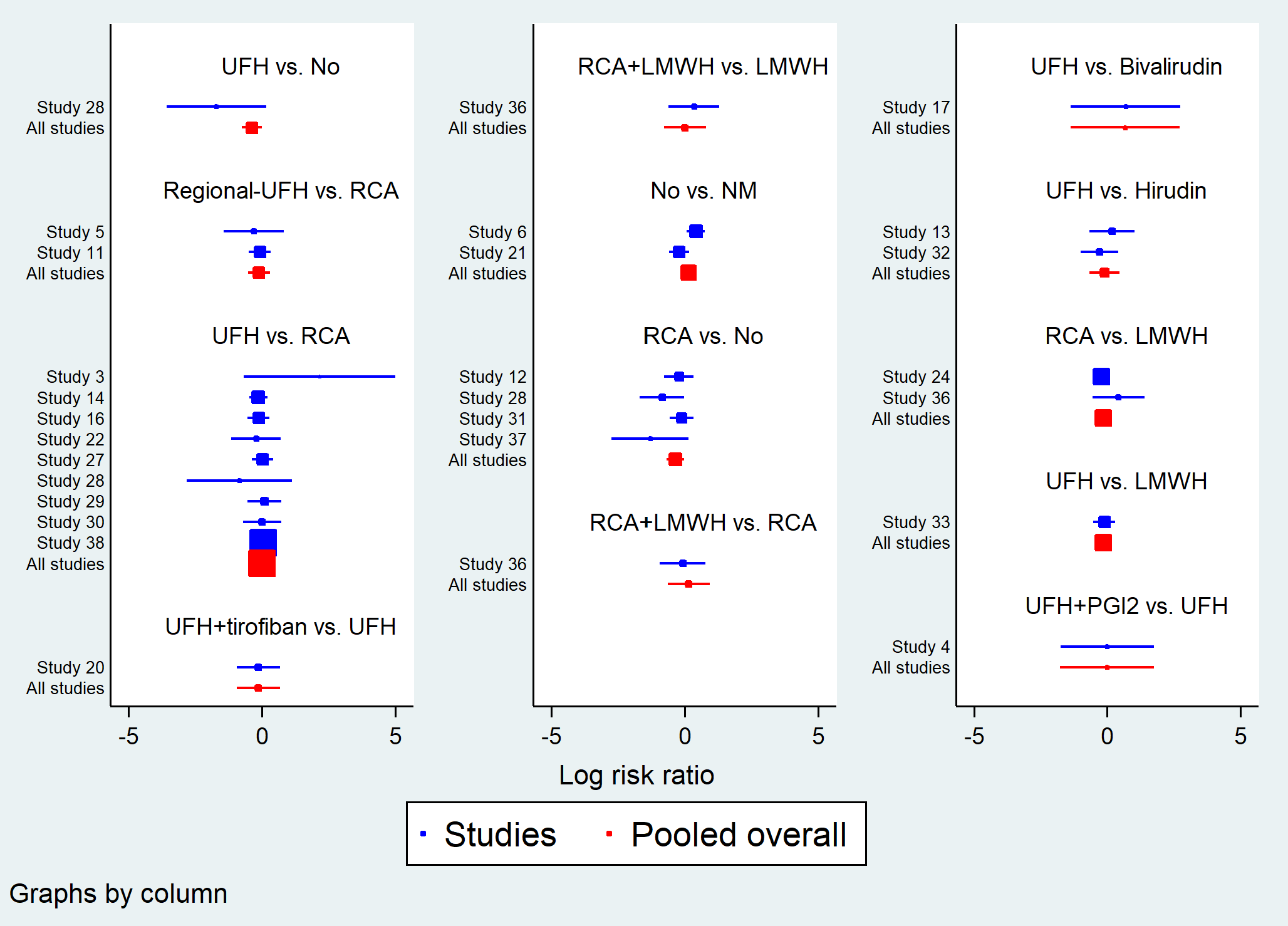


Figure S8. Forest plot in direct comparisons for evaluation of all-cause mortality.


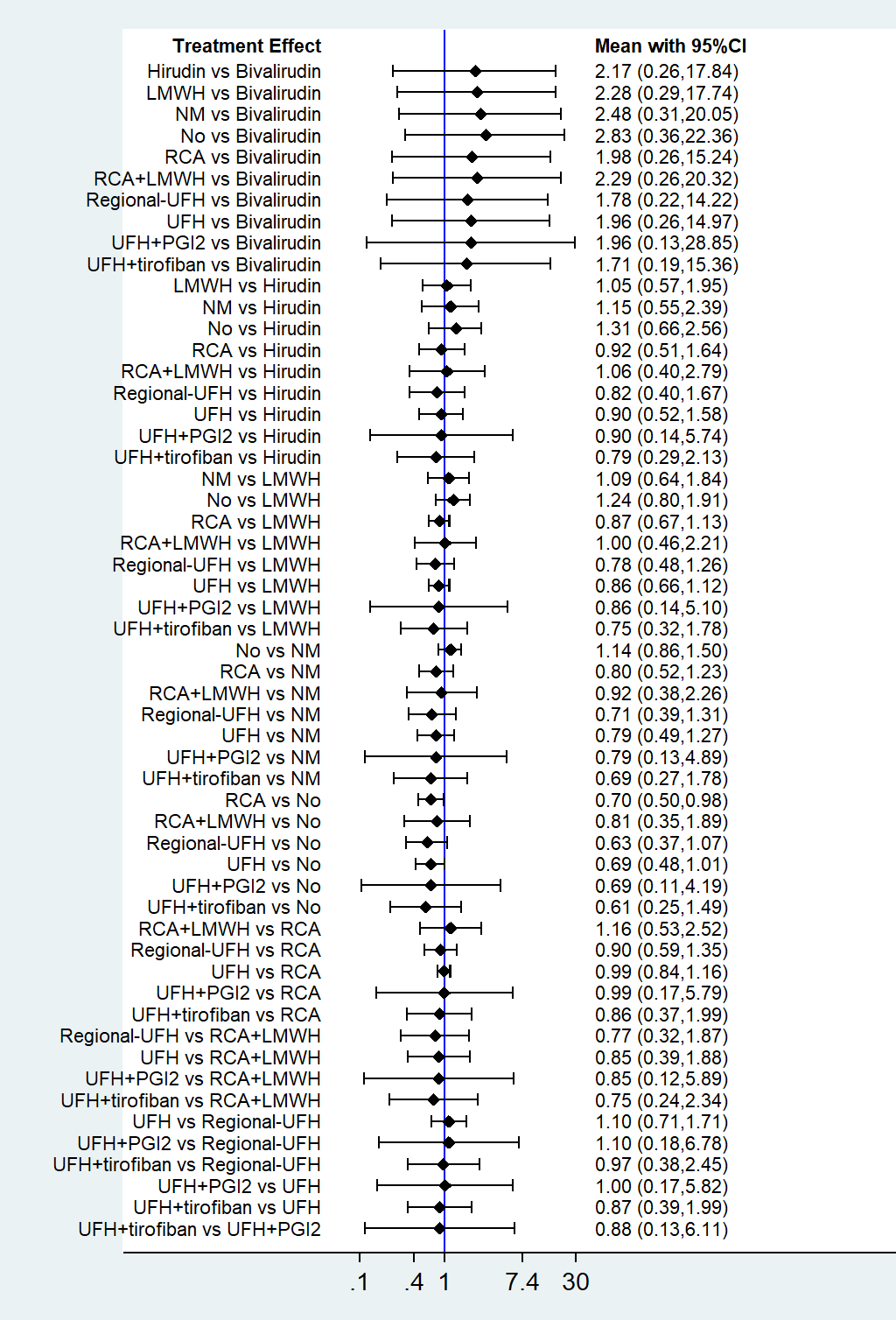


Figure S9. Forest plot of network meta-analysis for all-cause mortality.


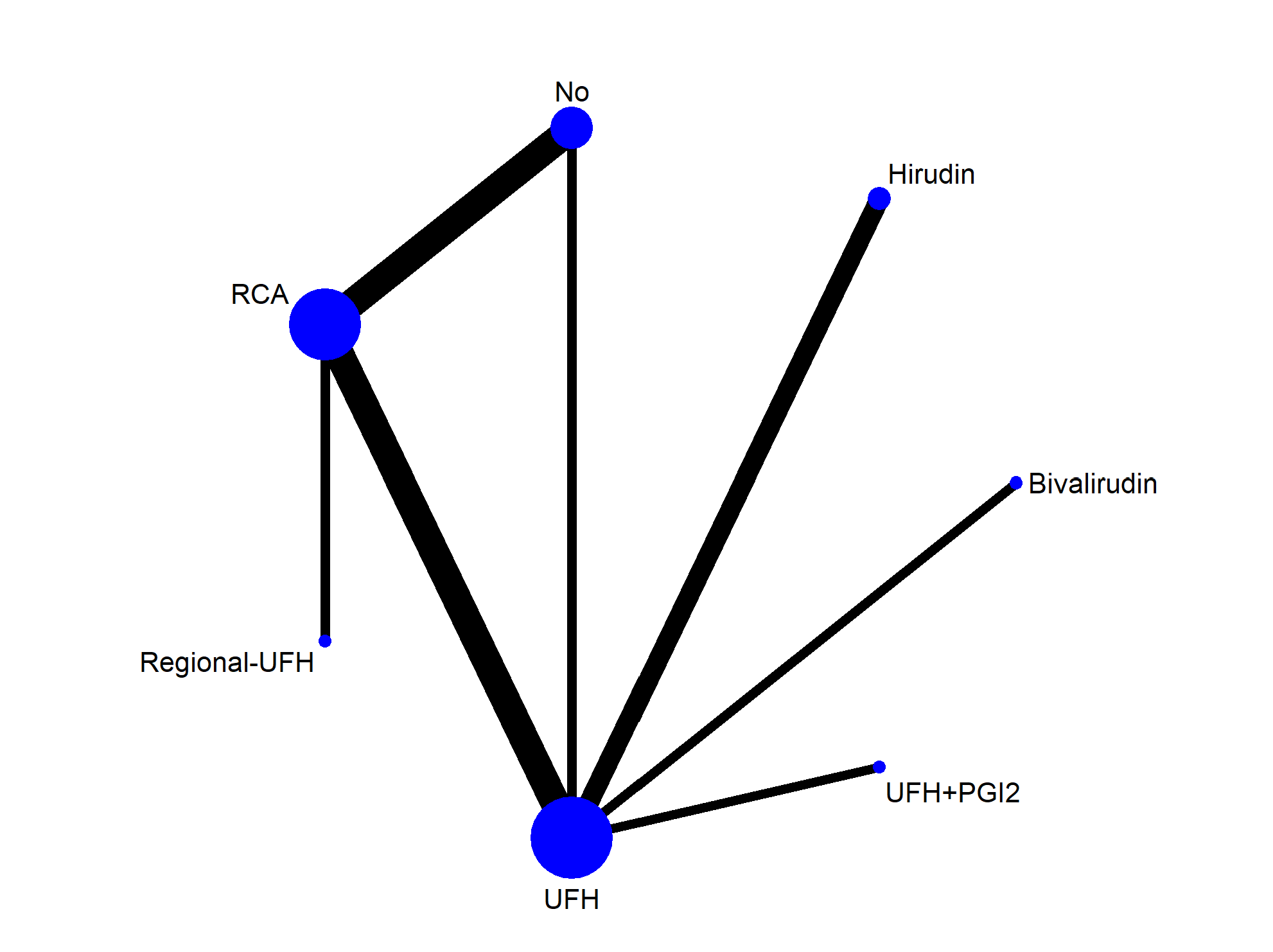


Figure S10. Network geometry of all the included anticoagulation options for evaluating length of ICU stay.


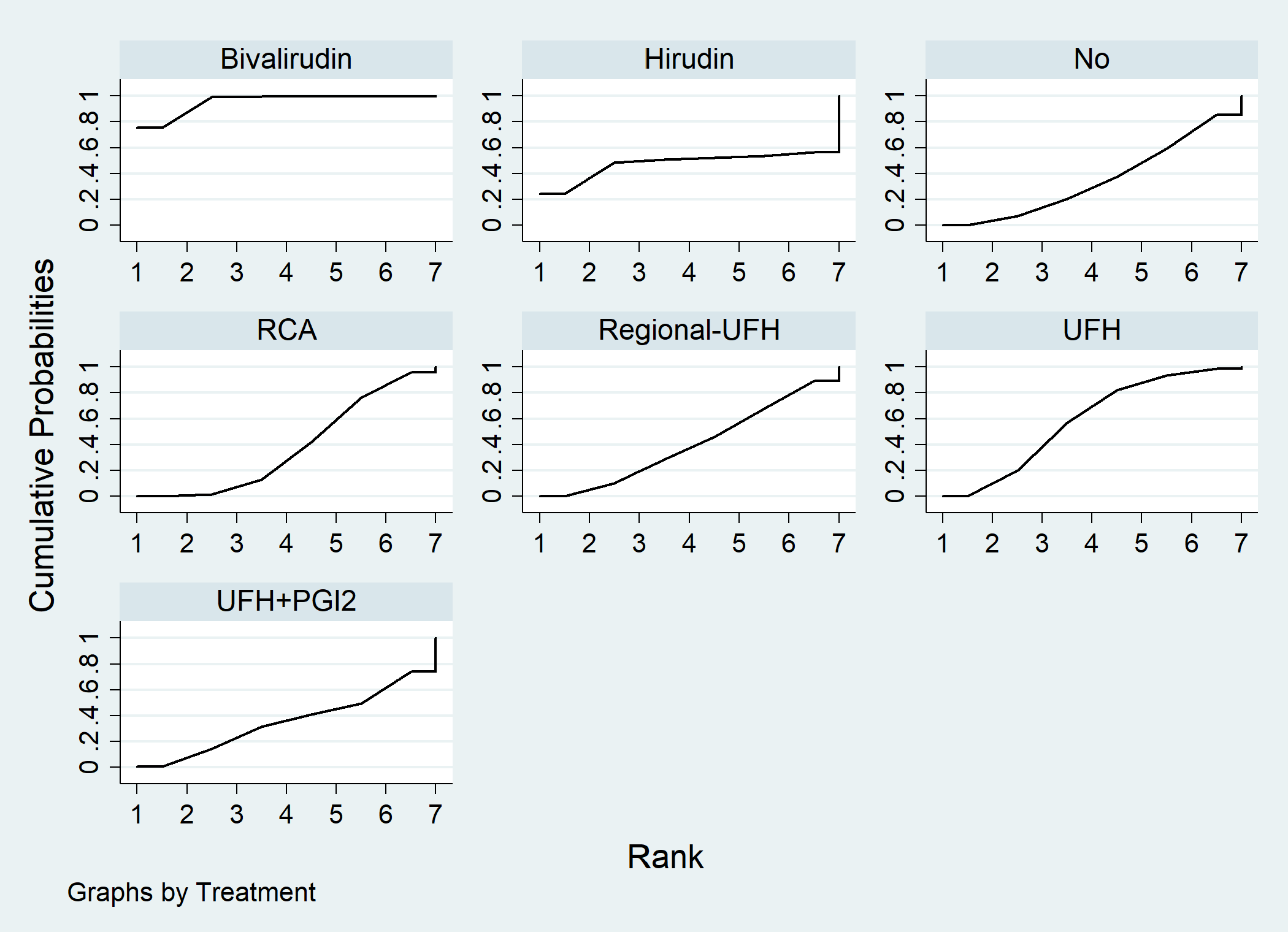


Figure S11. Length of ICU stay ranking among different anticoagulation options.


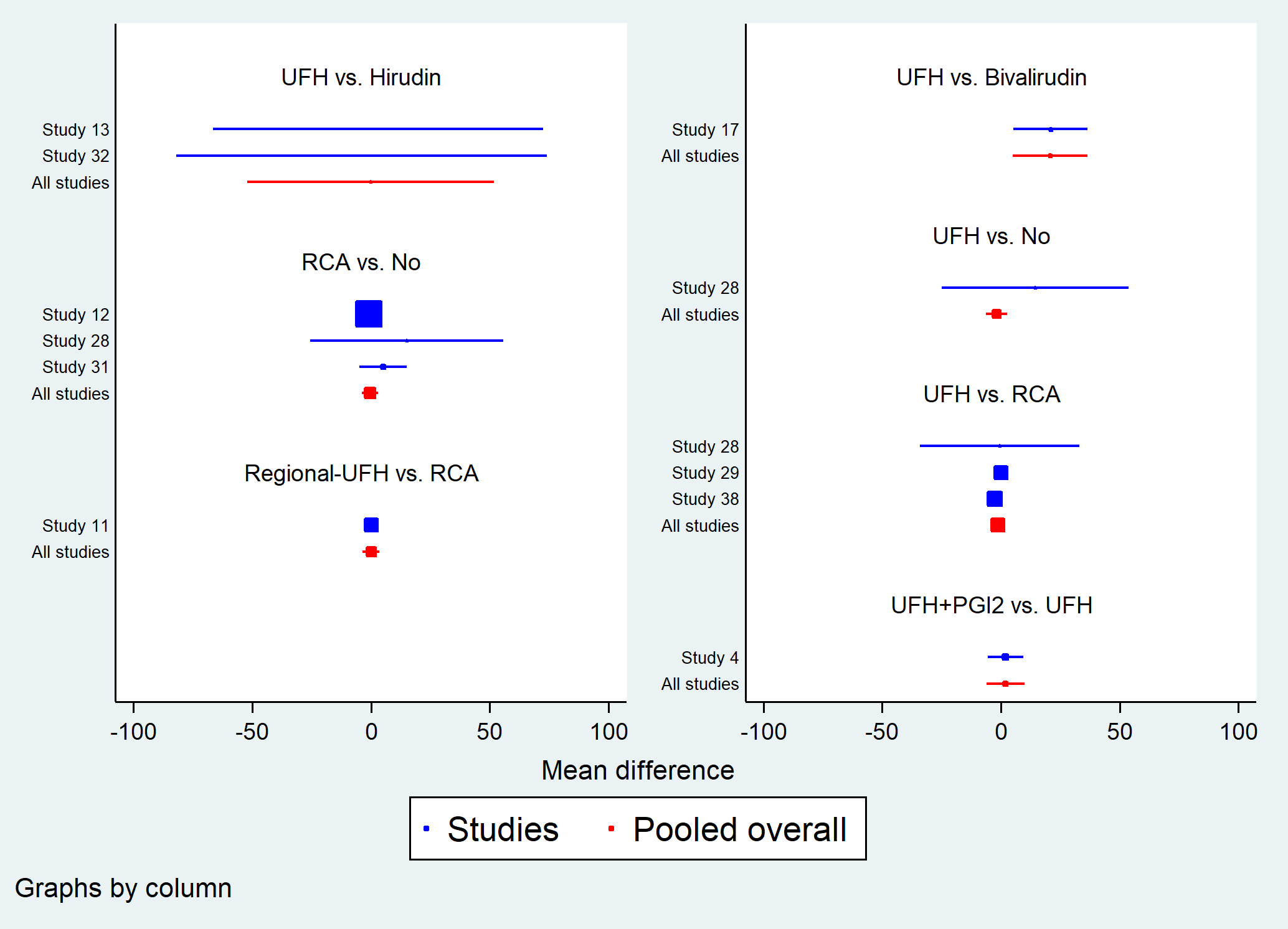


Figure S12. Forest plot in direct comparisons for evaluation of length of ICU stay.


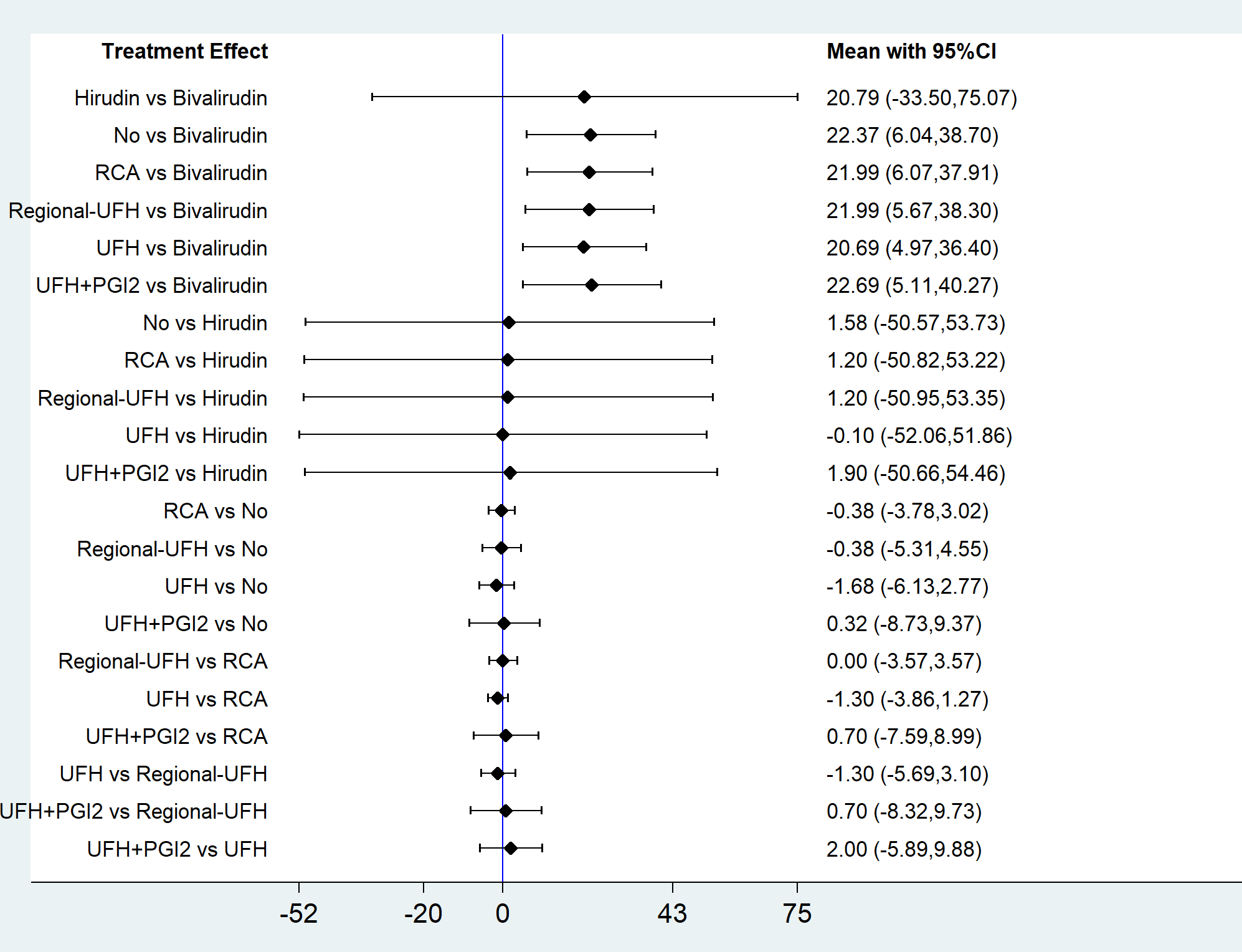


Figure S13. Forest plot of network meta-analysis for length of ICU stay.


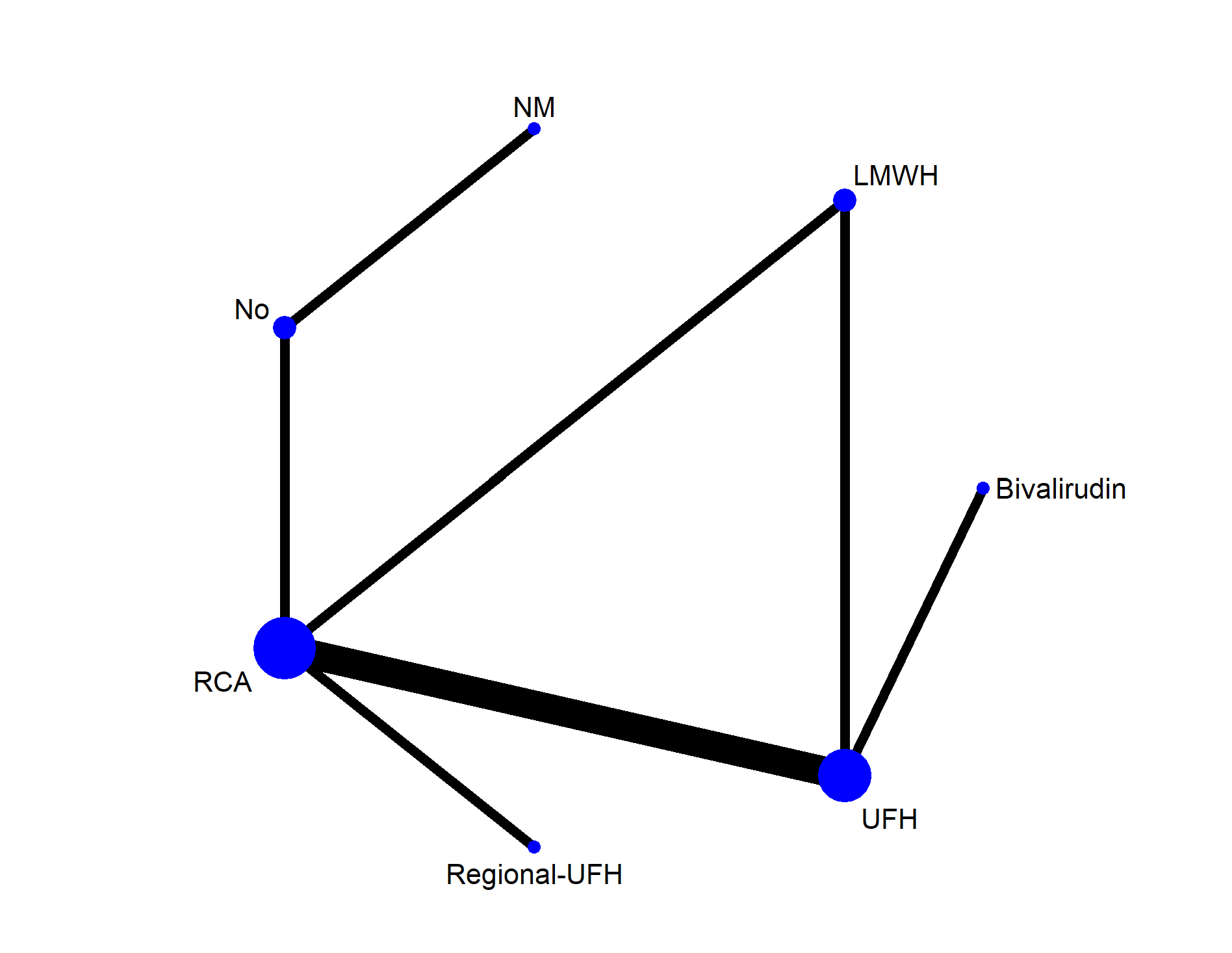


Figure S14. Network geometry of all the included anticoagulation options for evaluating duration of CRRT.


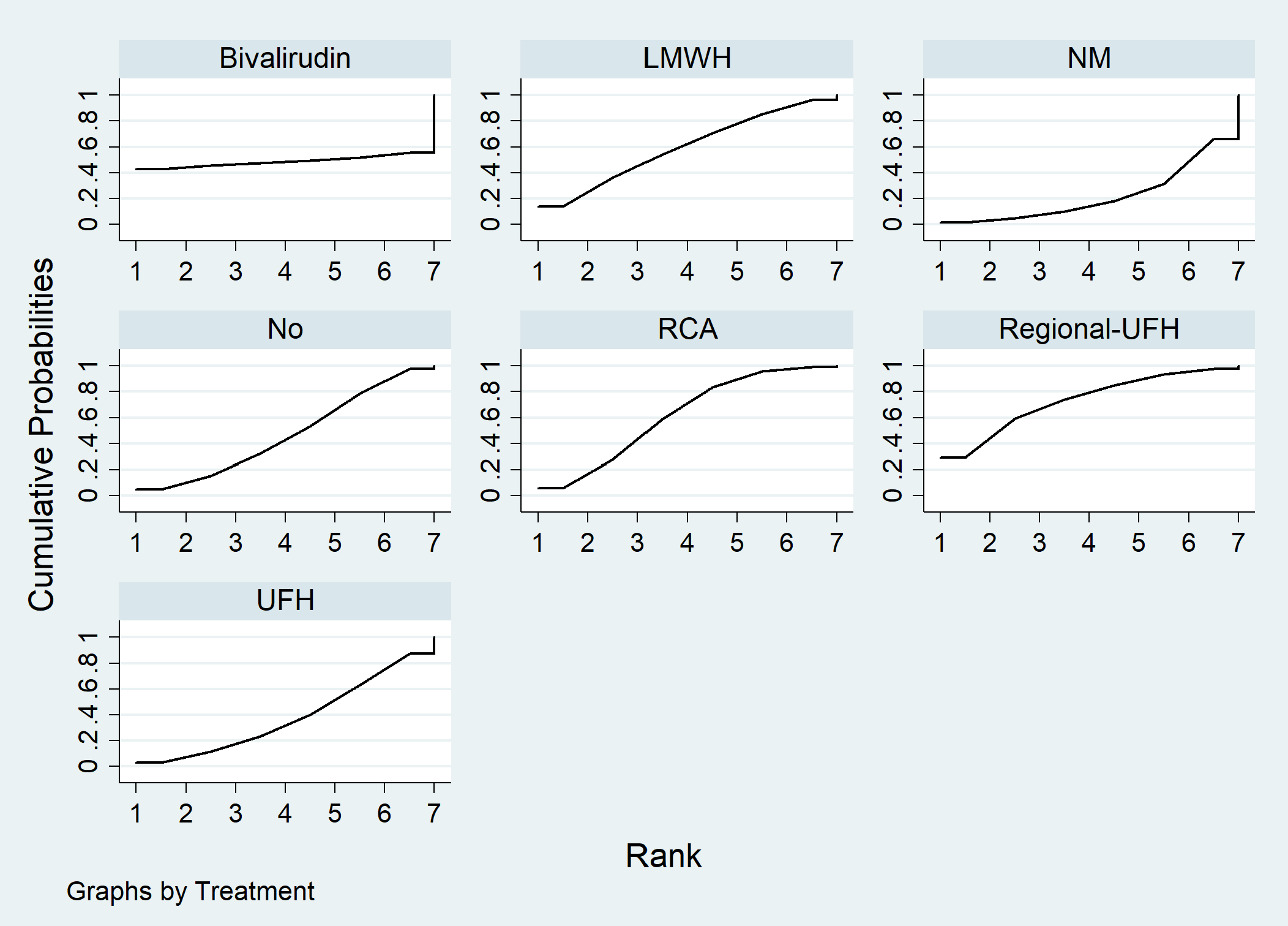


Figure S15. Duration of CRRT ranking among different anticoagulation options.


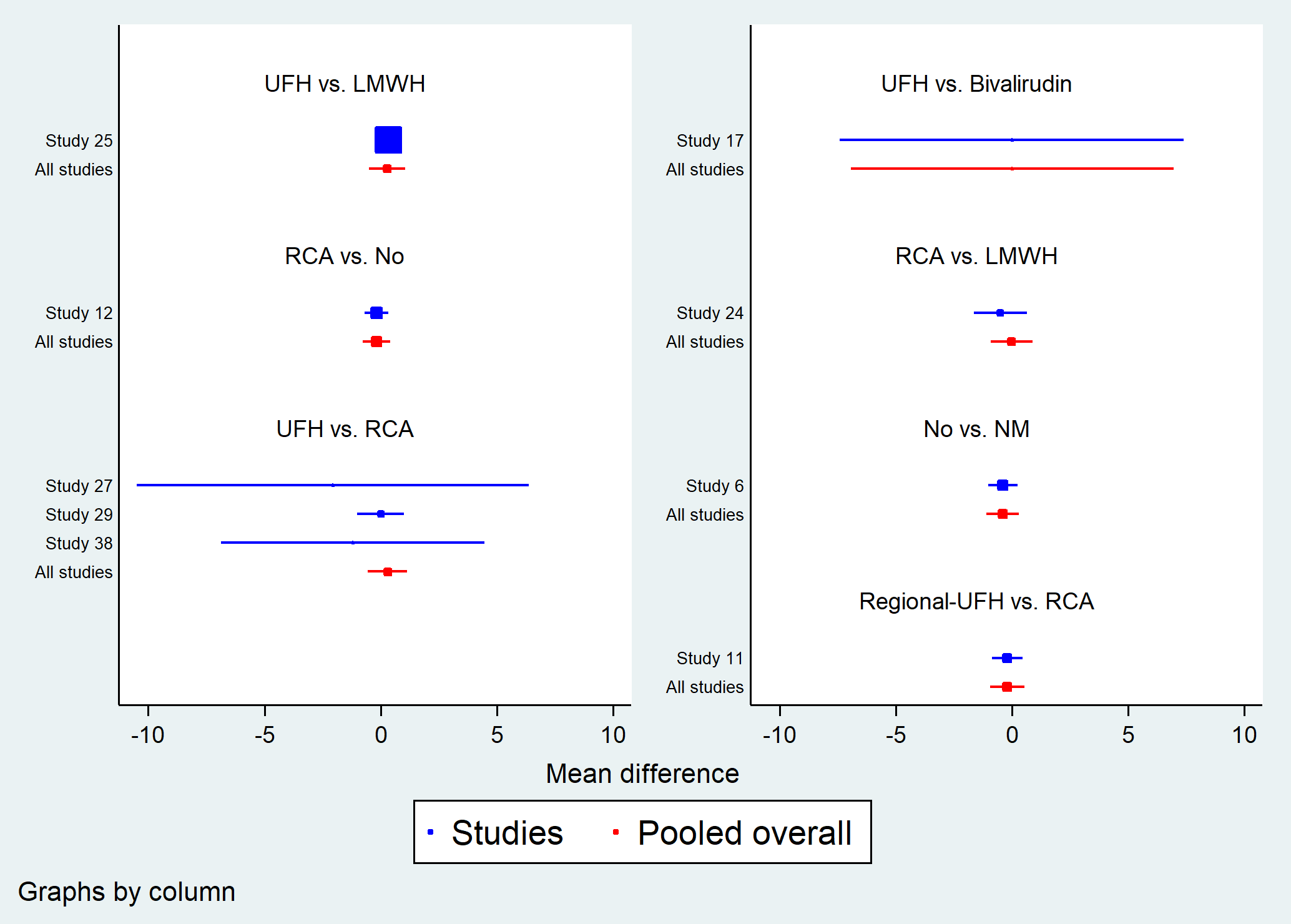


Figure S16. Forest plot in direct comparisons for evaluation of duration of CRRT.


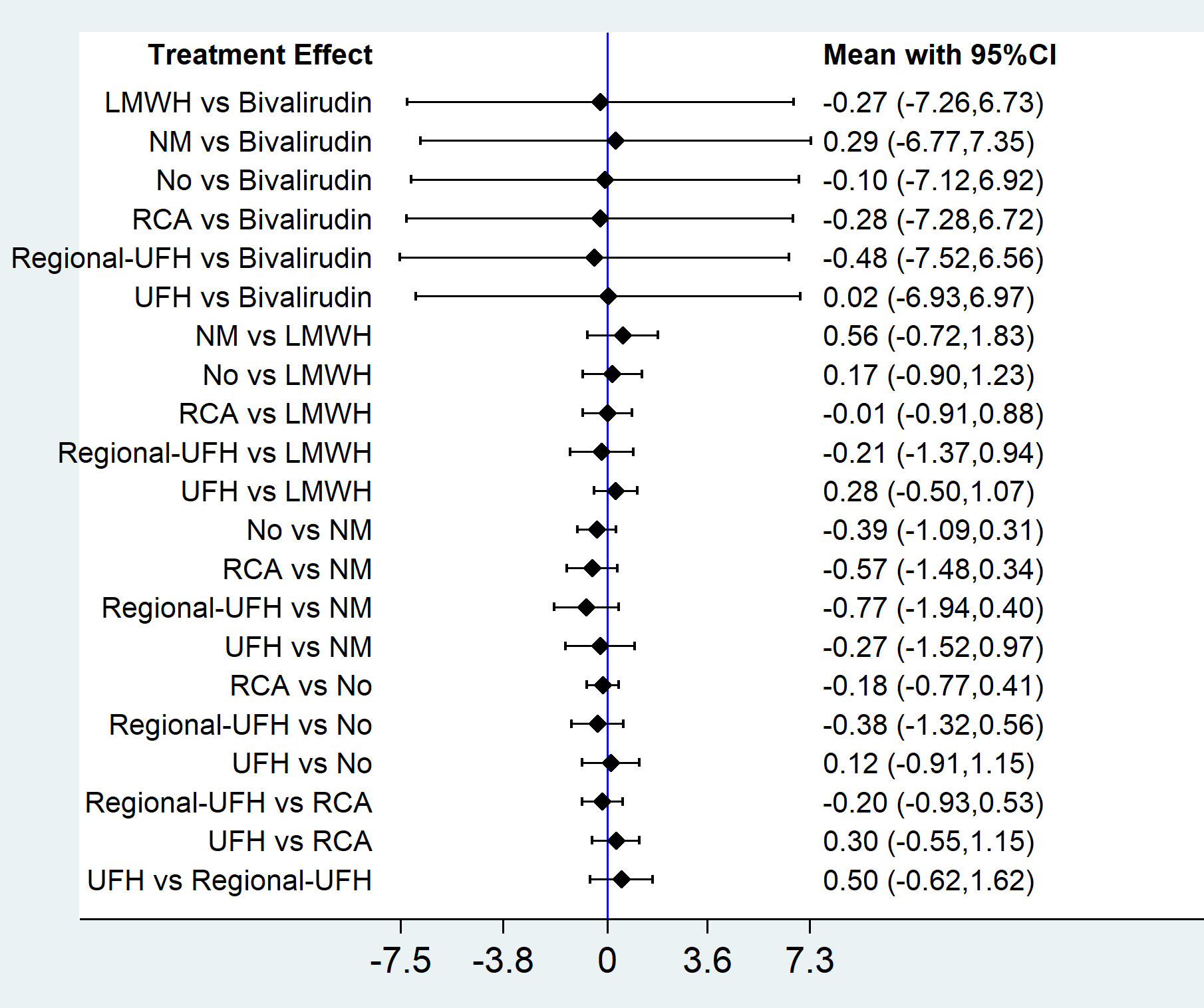


Figure S17. Forest plot of network meta-analysis for duration of CRRT.


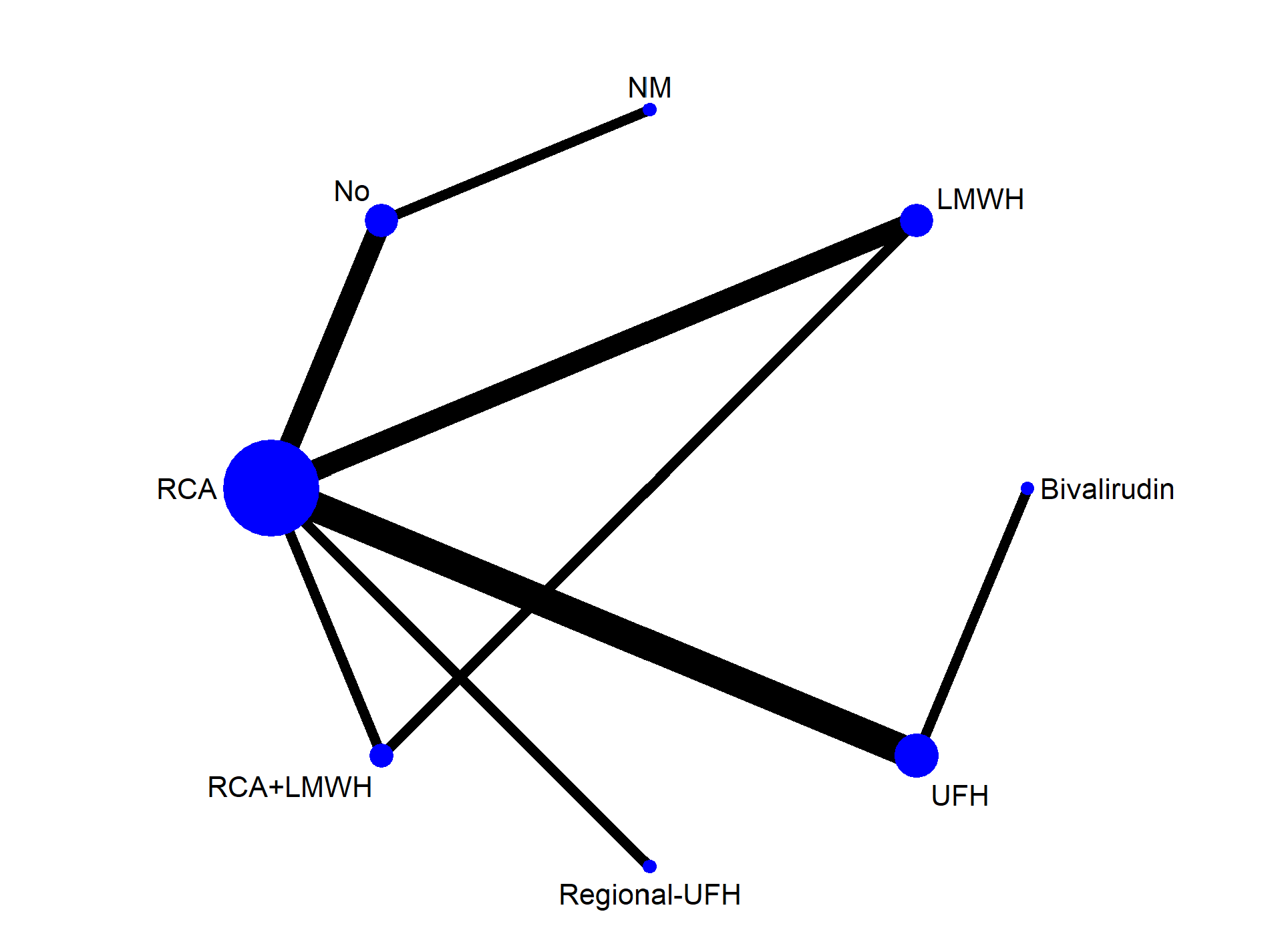


Figure S18. Network geometry of all the included anticoagulation options for evaluating recovery of renal function.


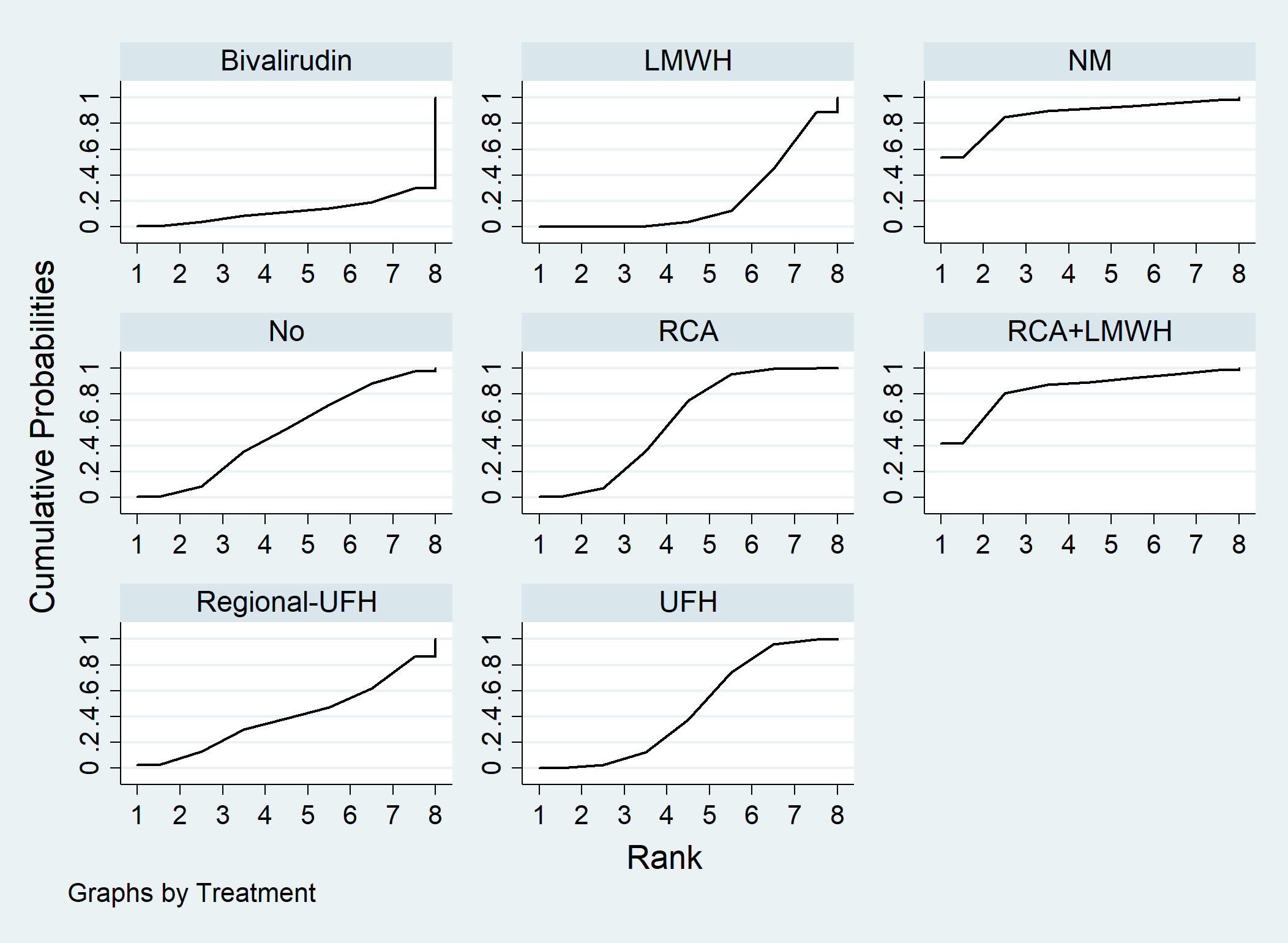


Figure S19. Recovery of renal function ranking among different anticoagulation options.


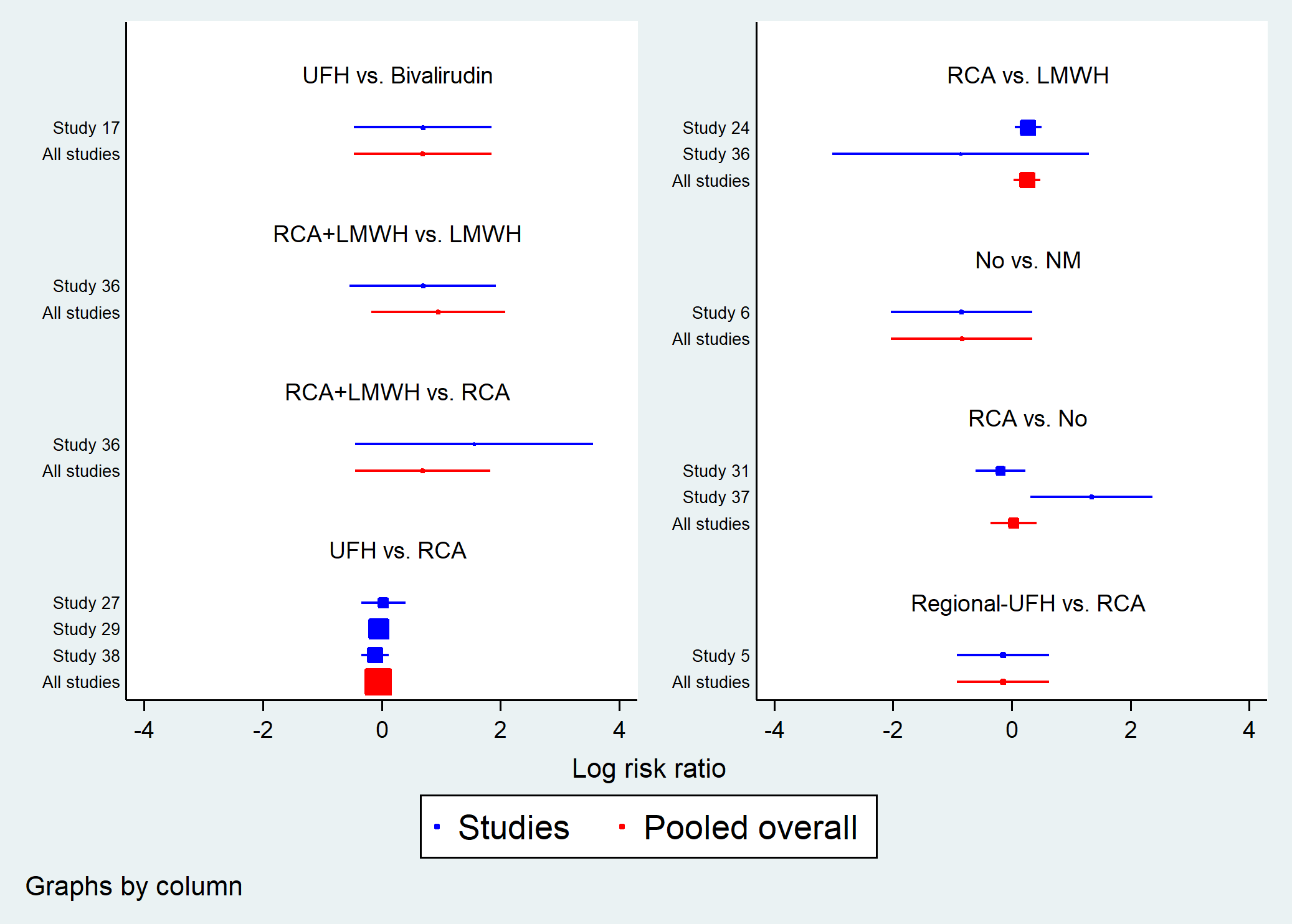


Figure S20. Forest plot in direct comparisons for evaluation of recovery of renal function.


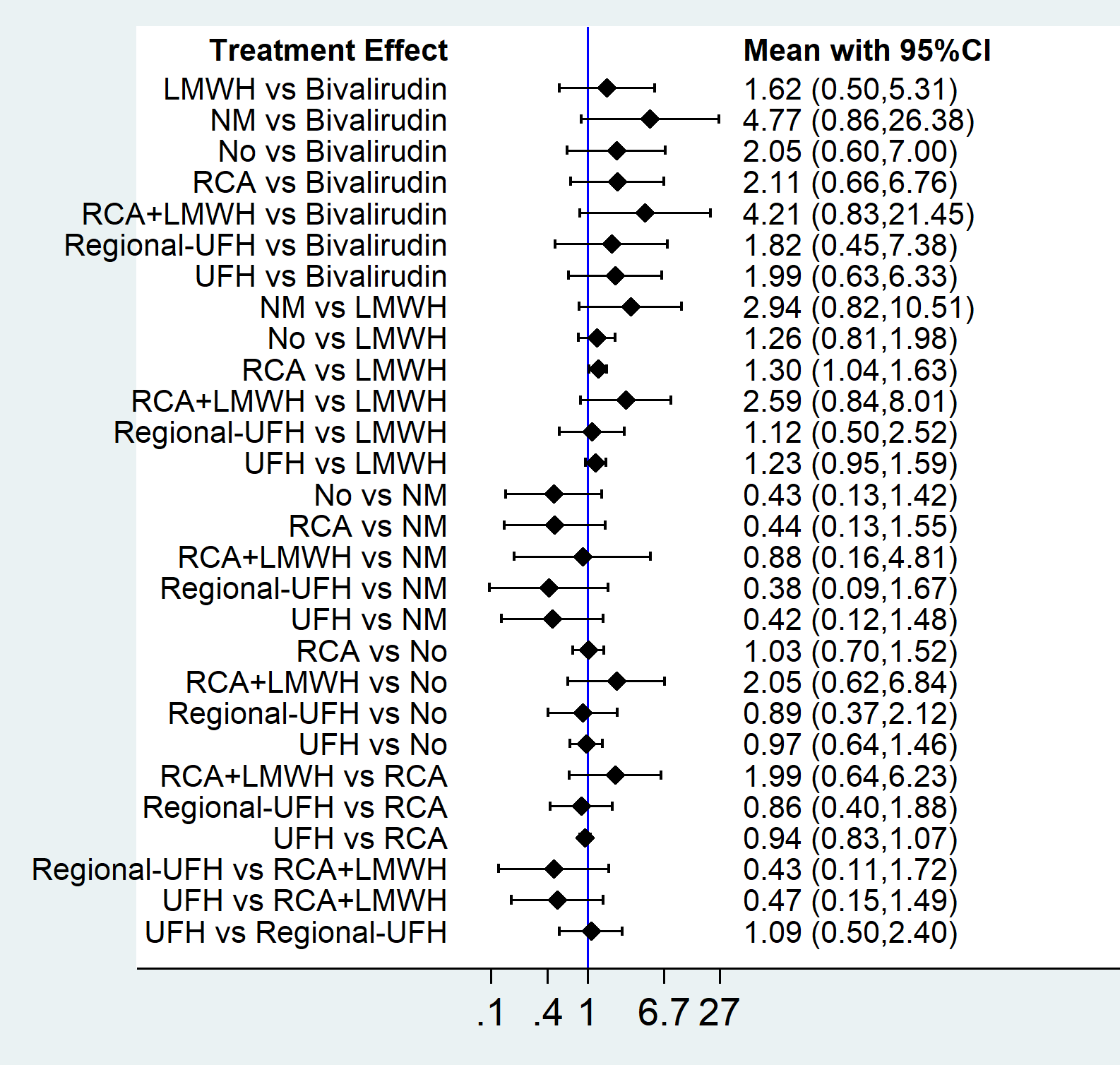


Figure S21. Forest plot of network meta-analysis for recovery of renal function.


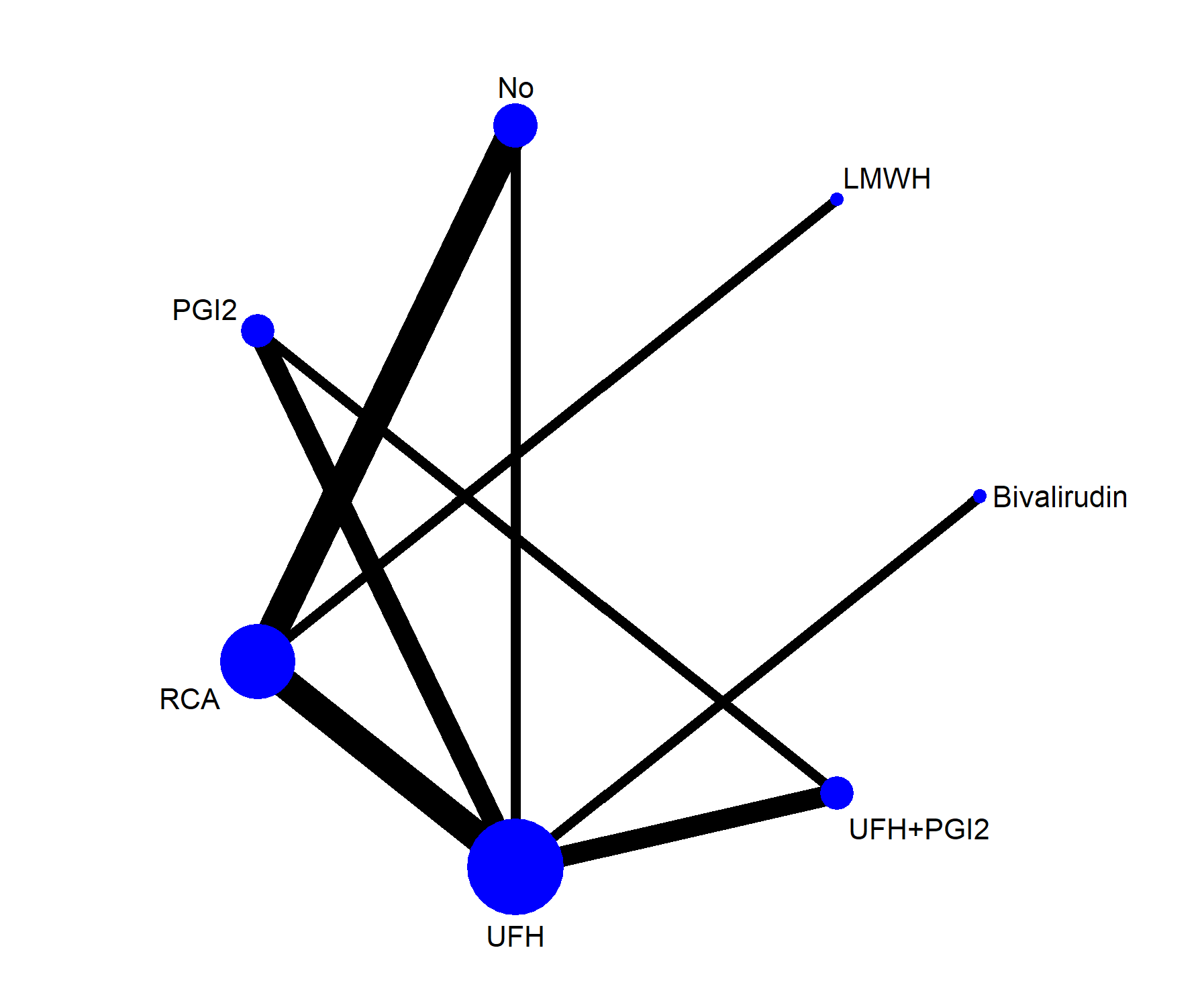


Figure S22. Network geometry of all the included anticoagulation options for evaluating reduction of Cr.


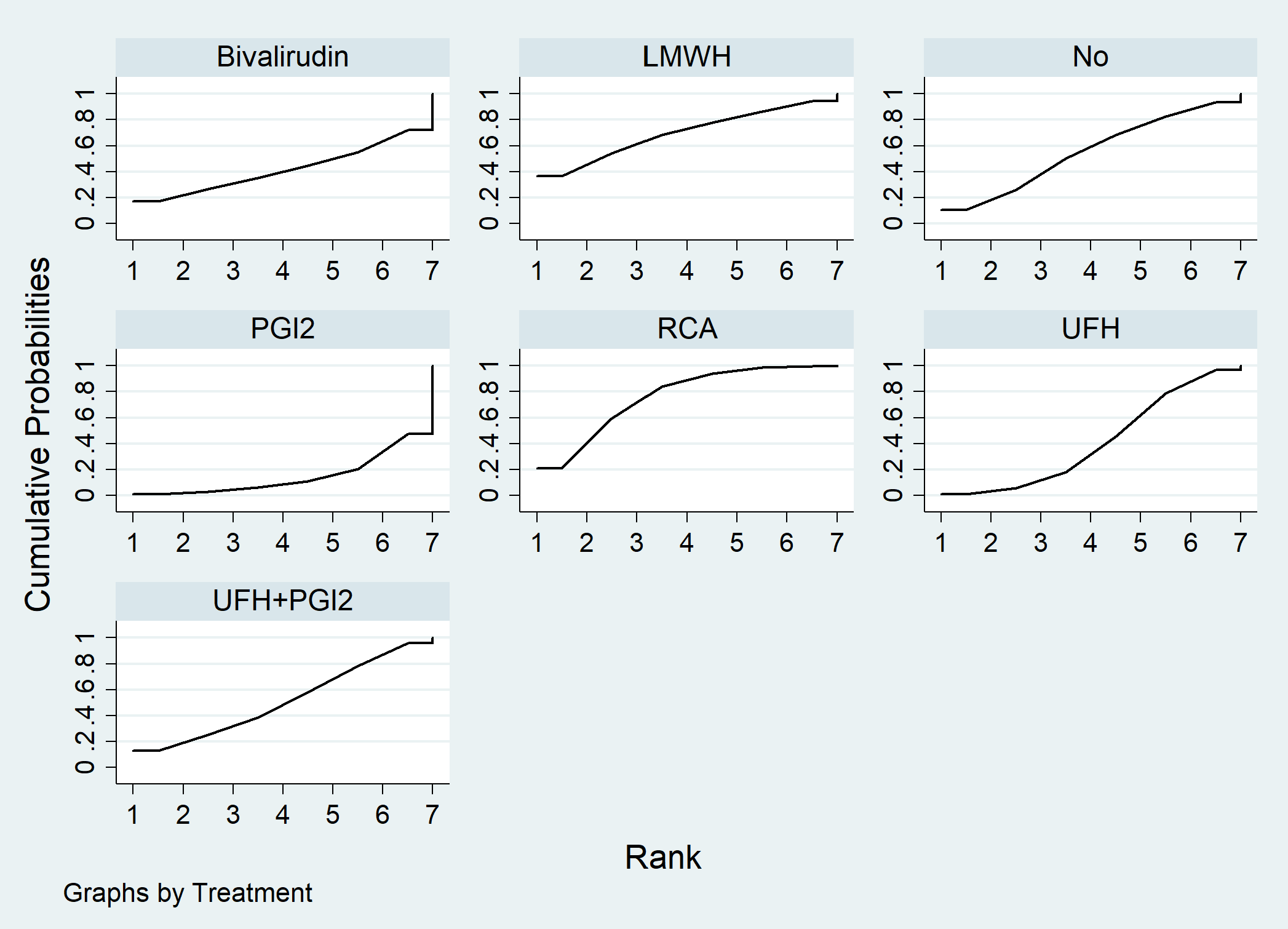


Figure S23. Reduction of Cr ranking among different anticoagulation options.


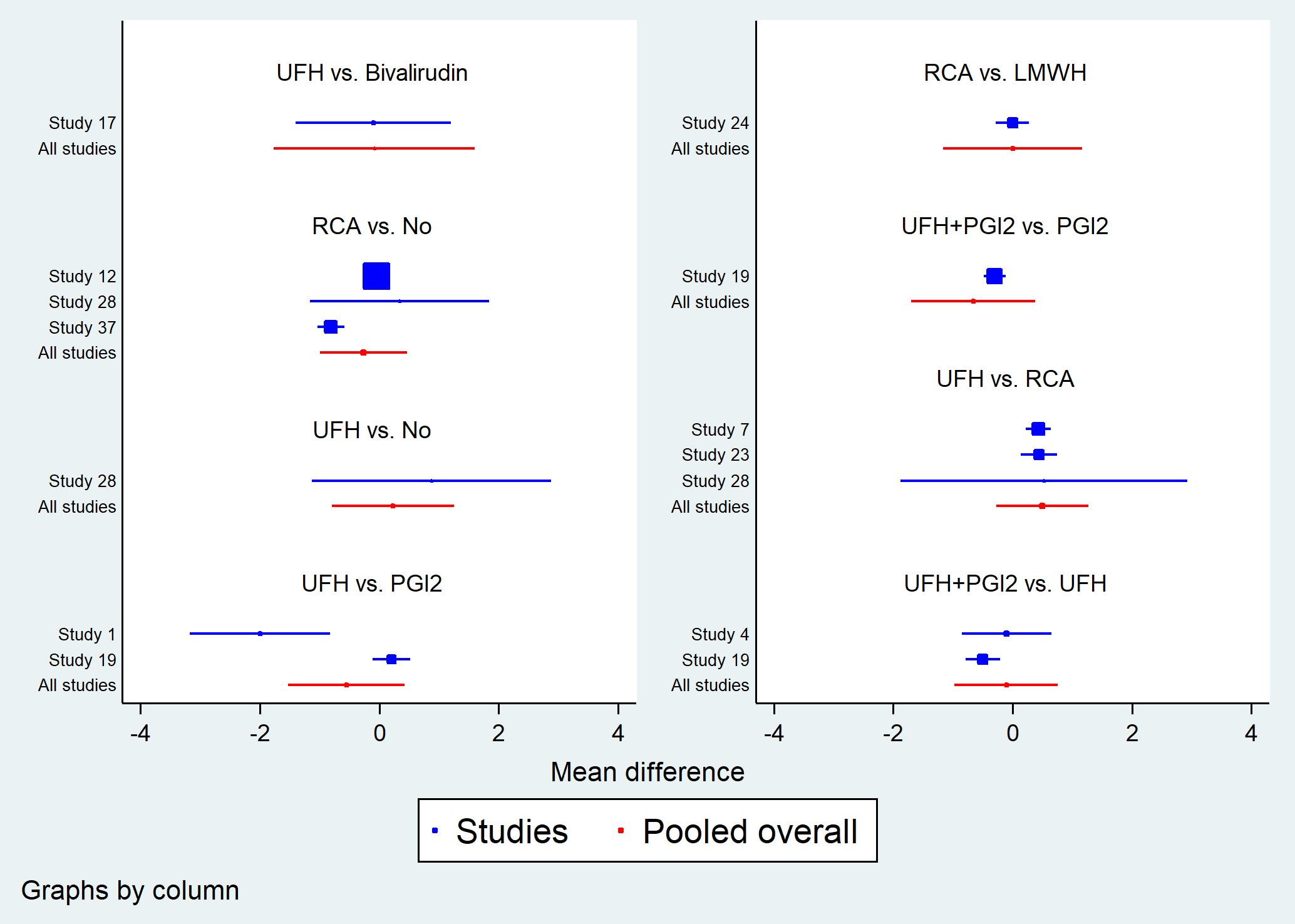


Figure S24. Forest plot in direct comparisons for evaluation of recovery of reduction of Cr.


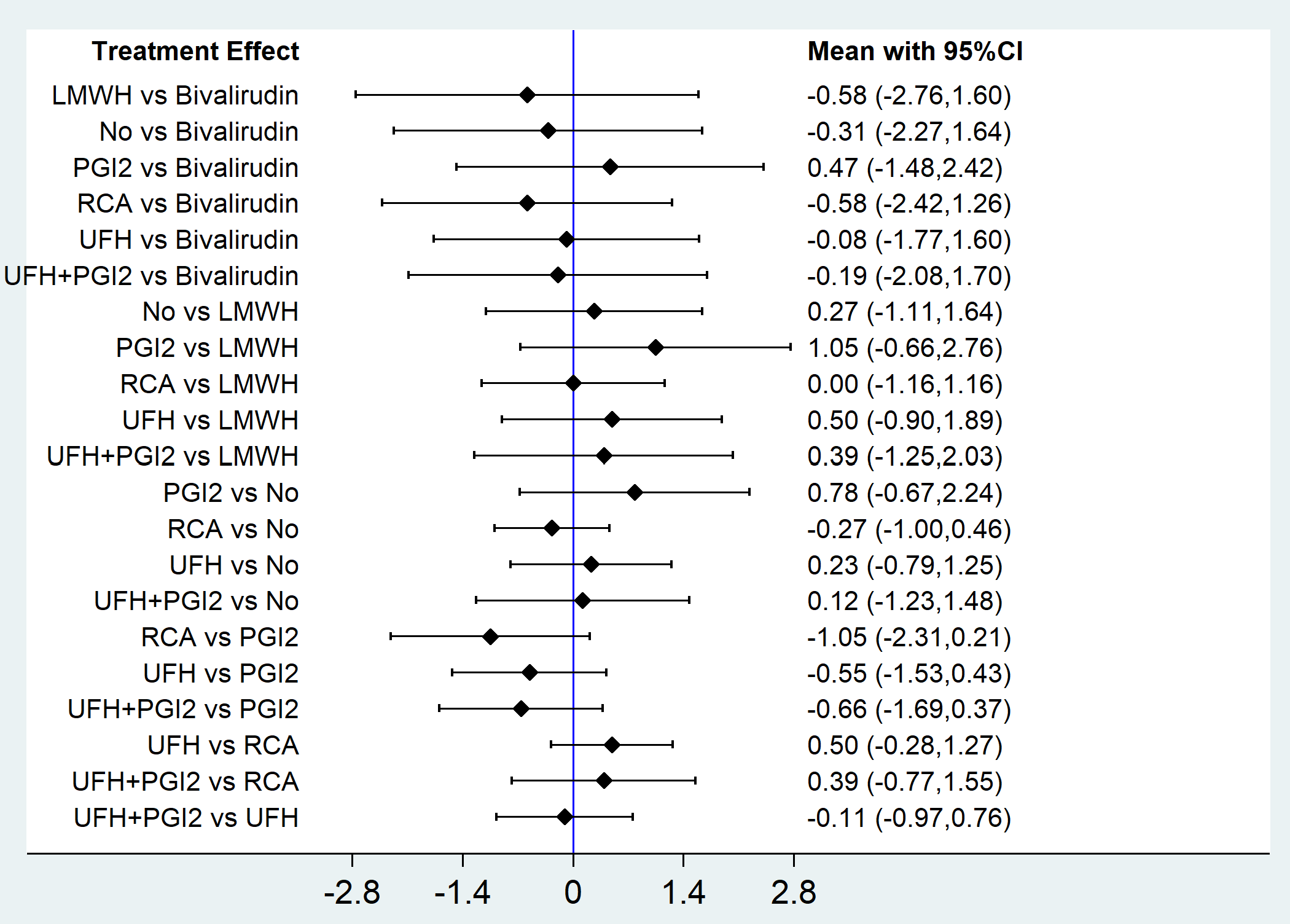


Figure S25. Forest plot of network meta-analysis for reduction of Cr.


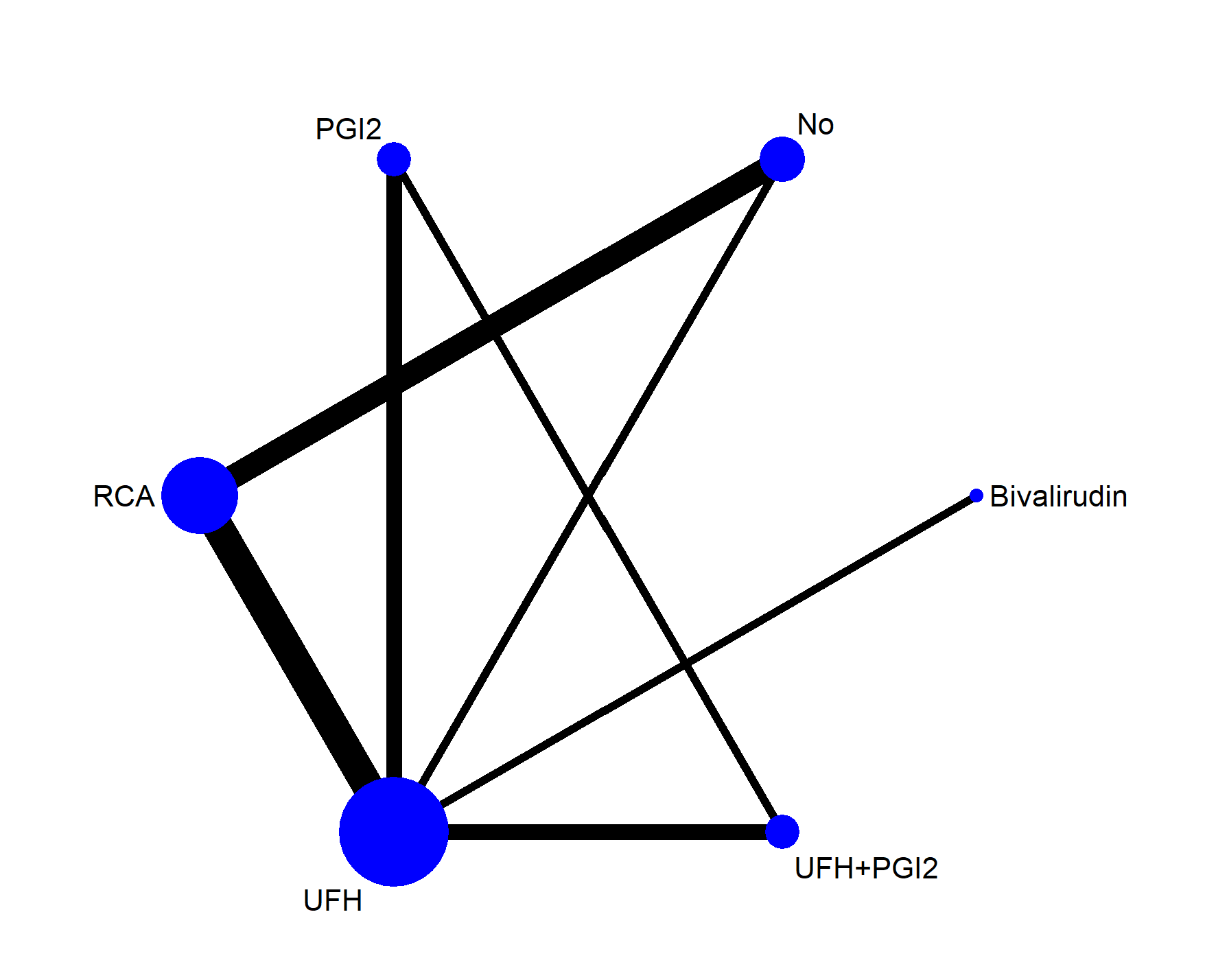


Figure S26. Network geometry of all the included anticoagulation options for evaluating reduction of BUN.


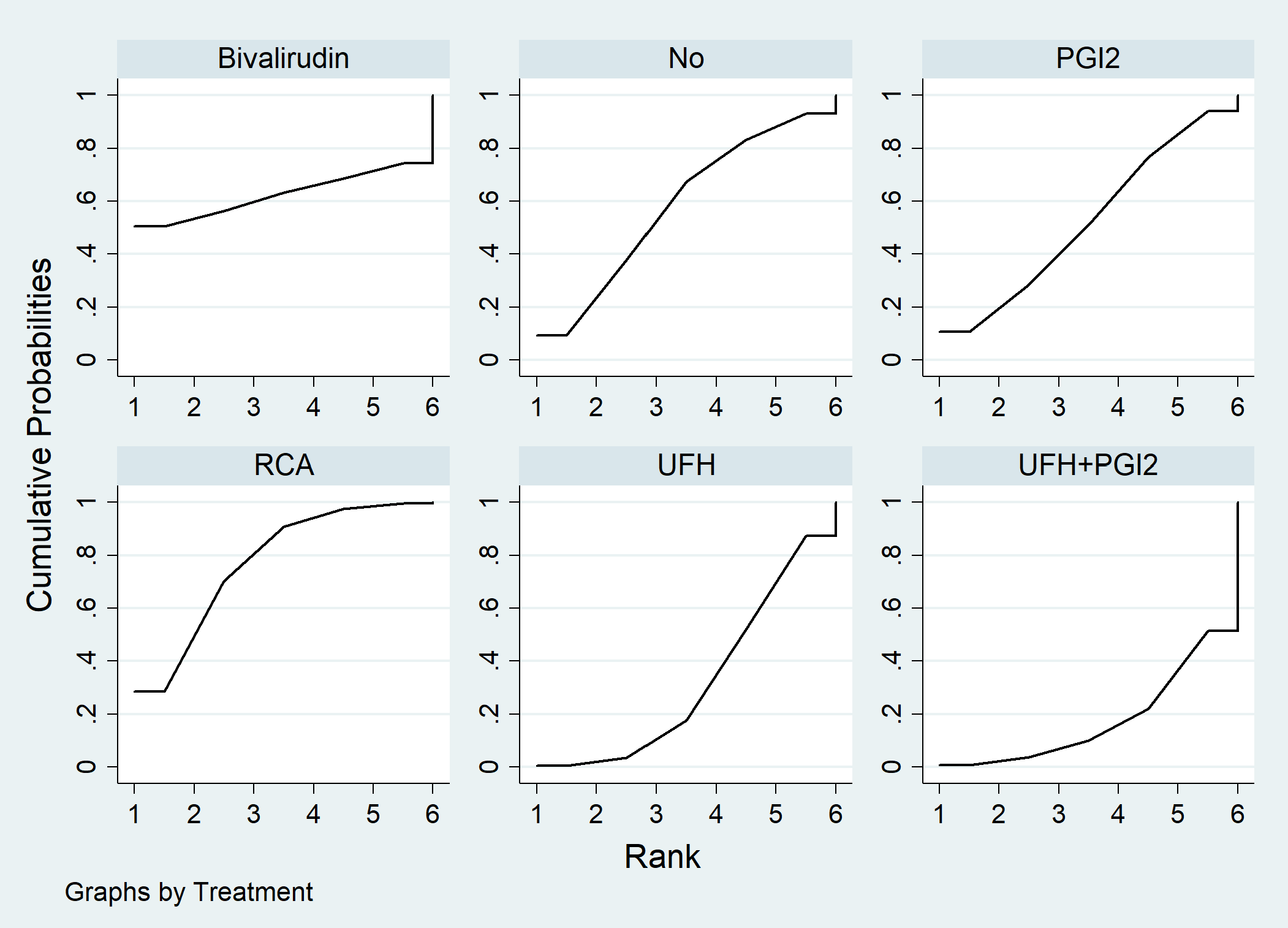


Figure S27. Reduction of BUN ranking among different anticoagulation options.


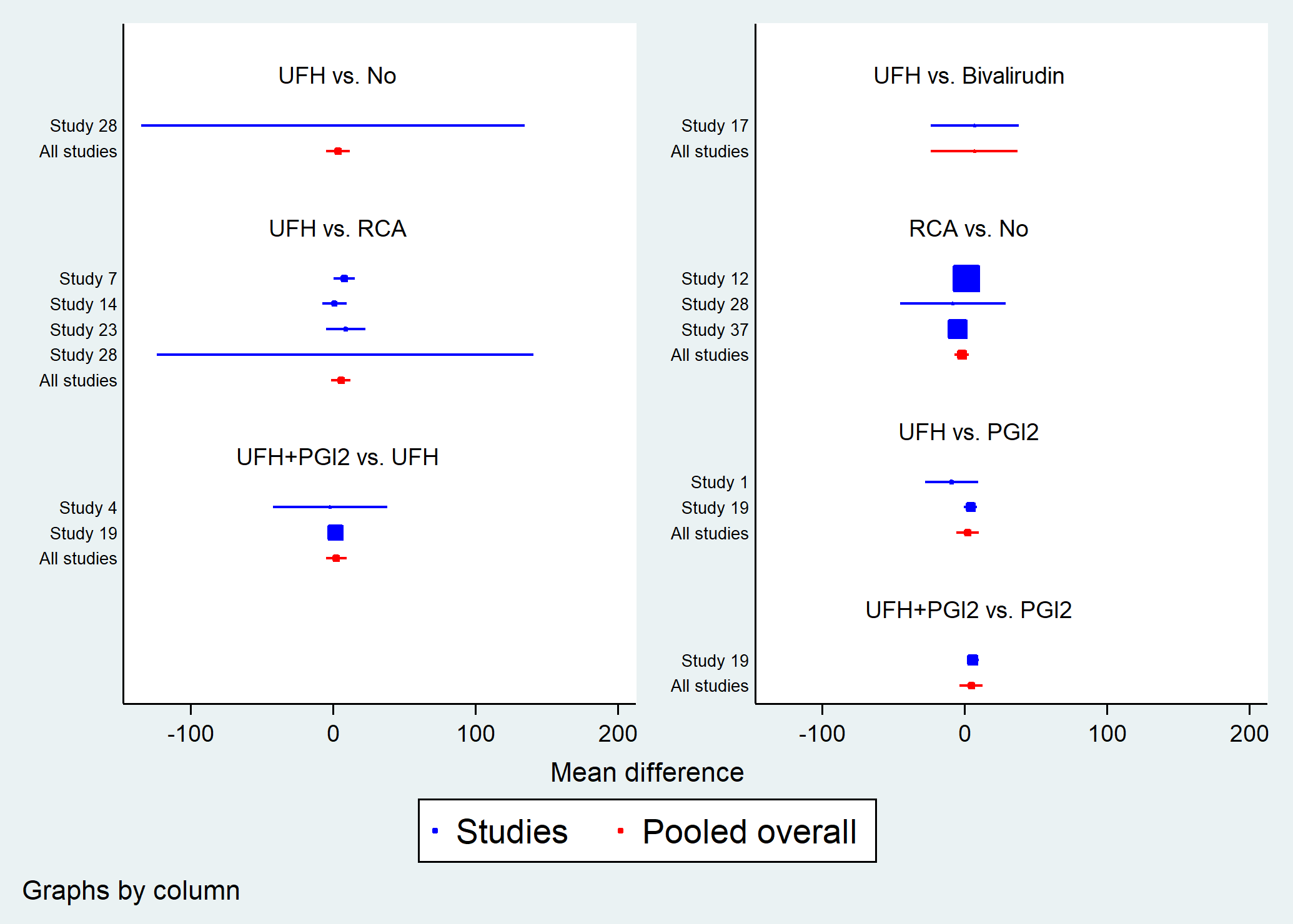


Figure S28. Forest plot in direct comparisons for evaluation of recovery of reduction of BUN.


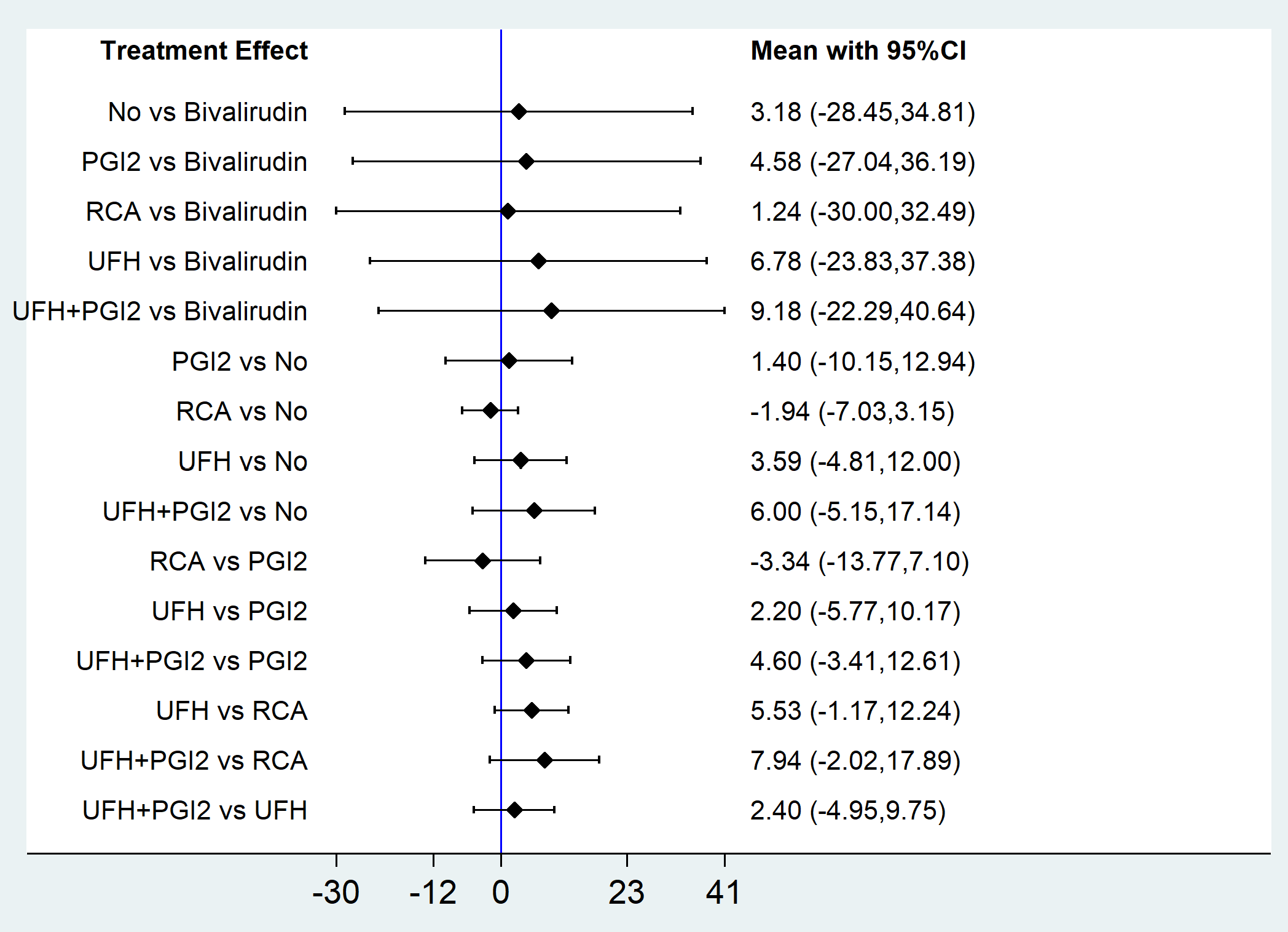


Figure S29. Forest plot of network meta-analysis for reduction of BUN.


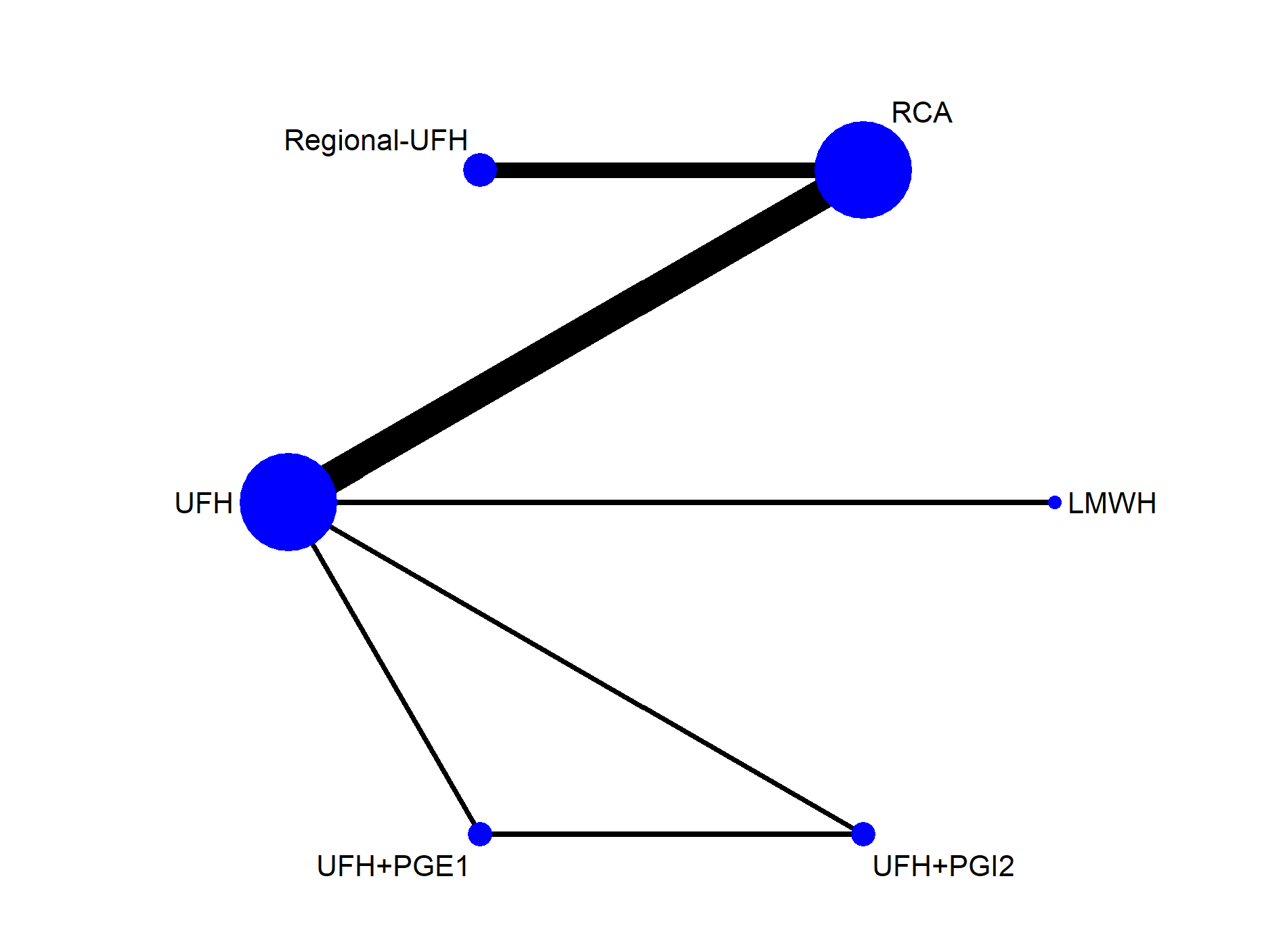


Figure S30. Network geometry of all the included anticoagulation options for evaluating adverse events.


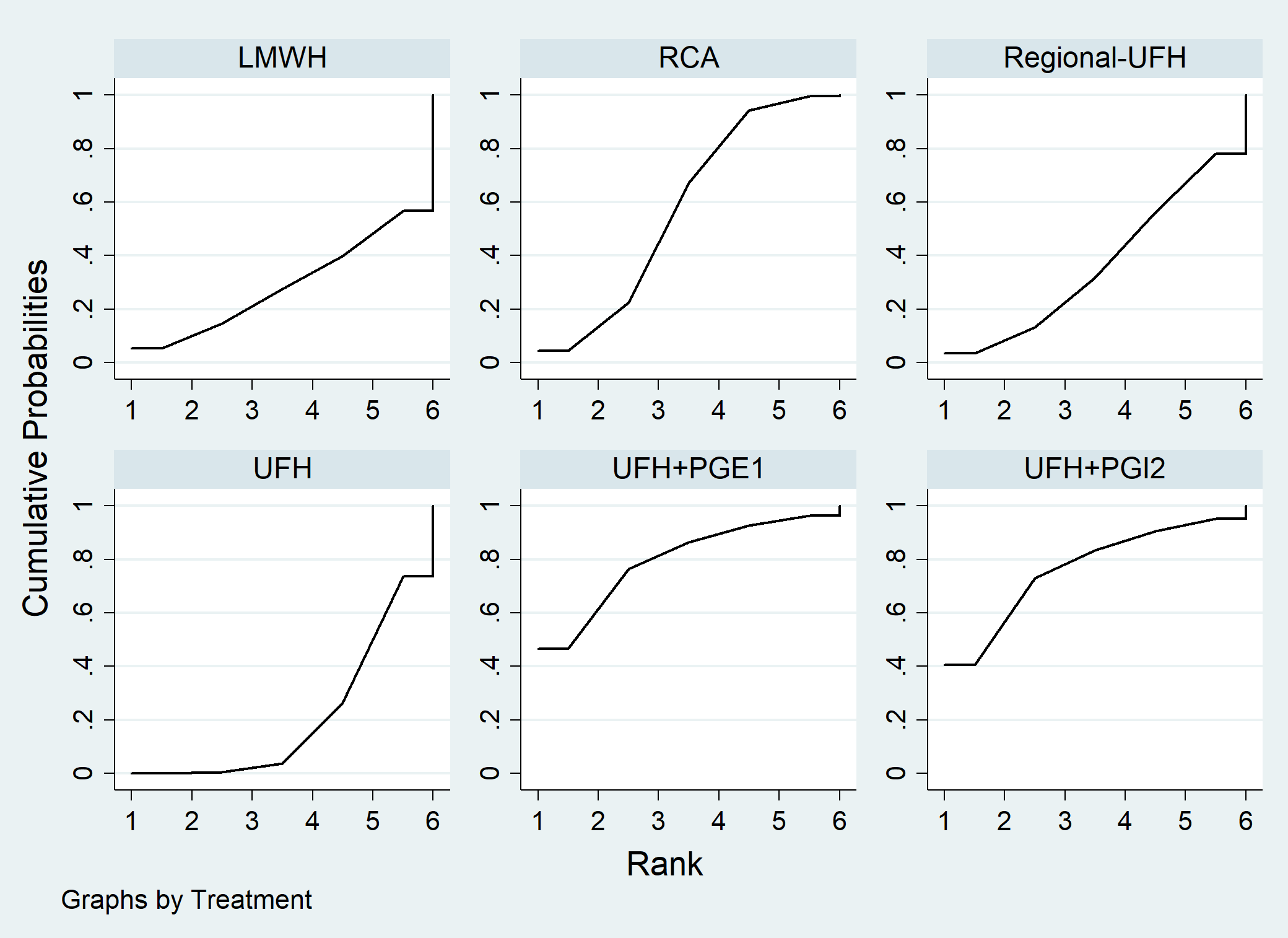


Figure S31. Adverse events ranking among different anticoagulation options.


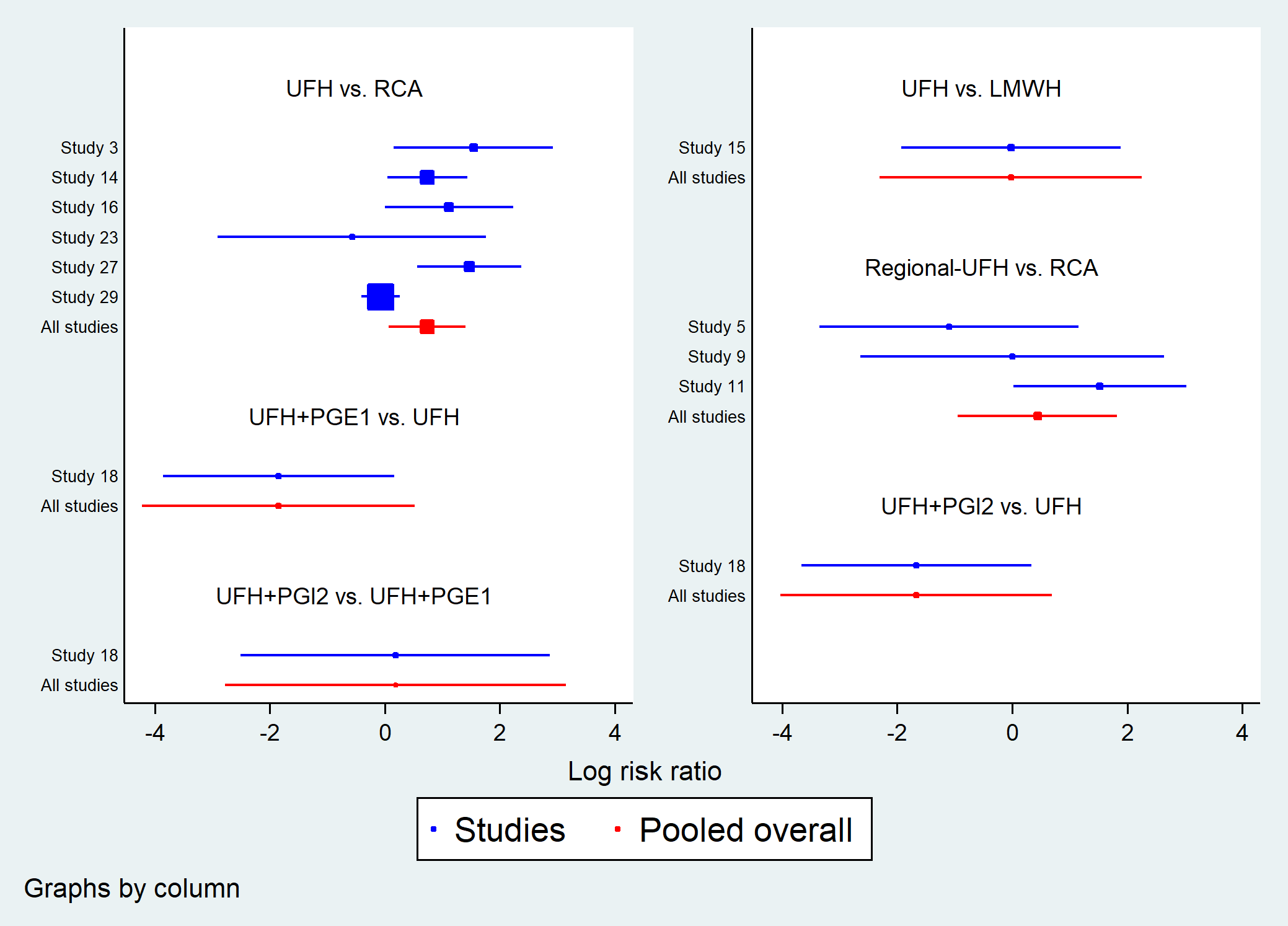


Figure S32. Forest plot in direct comparisons for evaluation of adverse events.


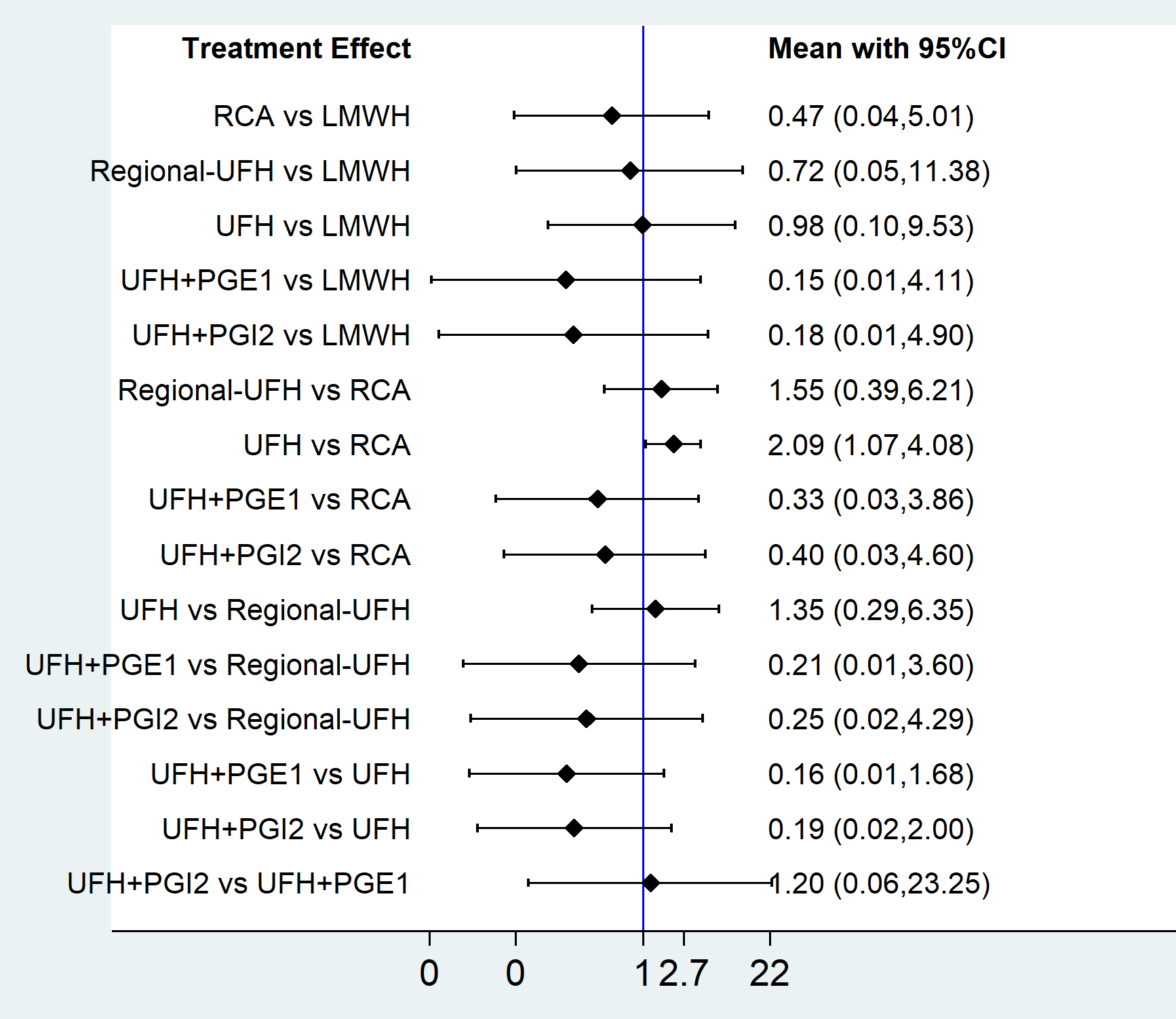


Figure S33. Forest plot of network meta-analysis for adverse events.


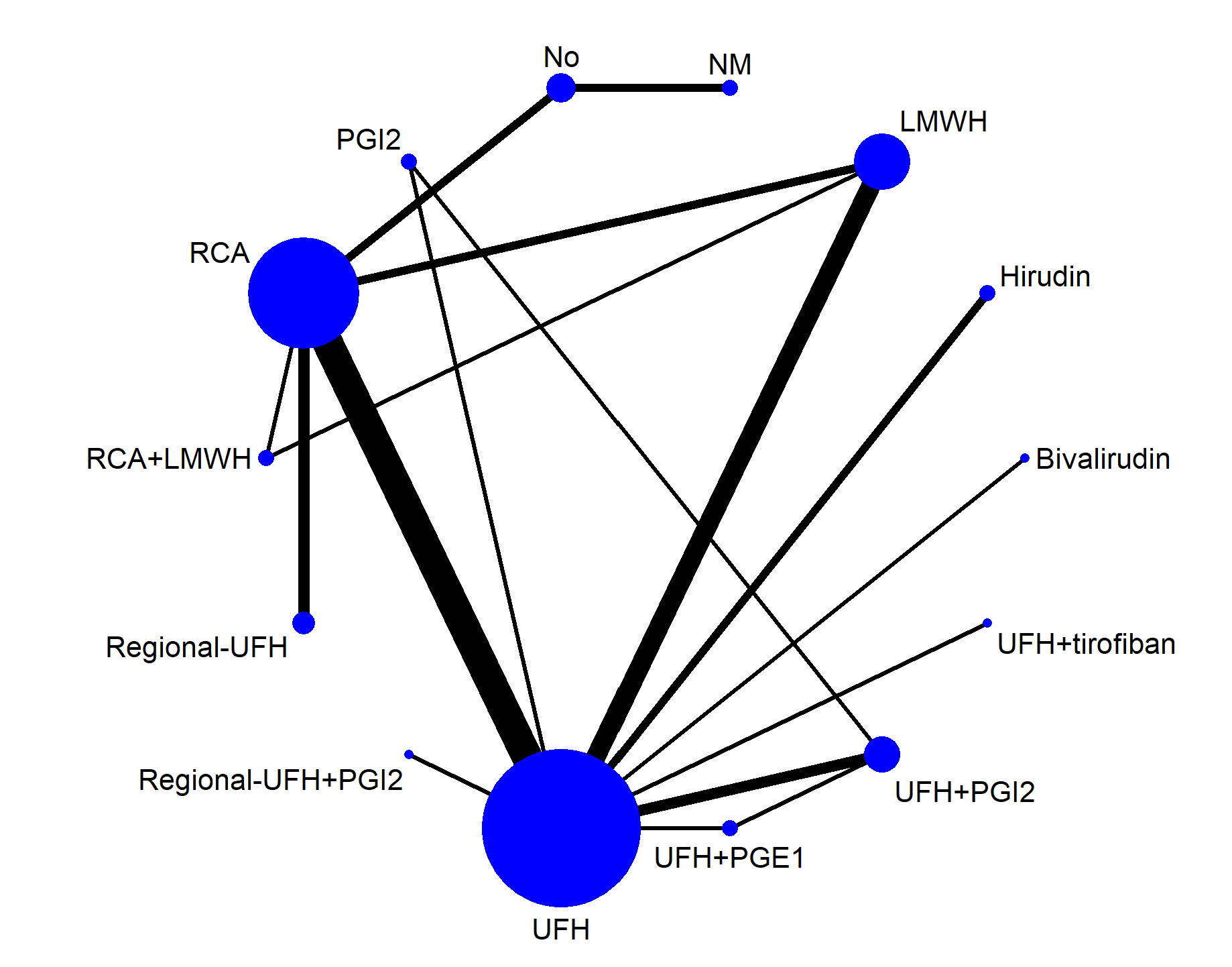


Figure S34. Network geometry of all the included anticoagulation options for evaluating bleeding events.


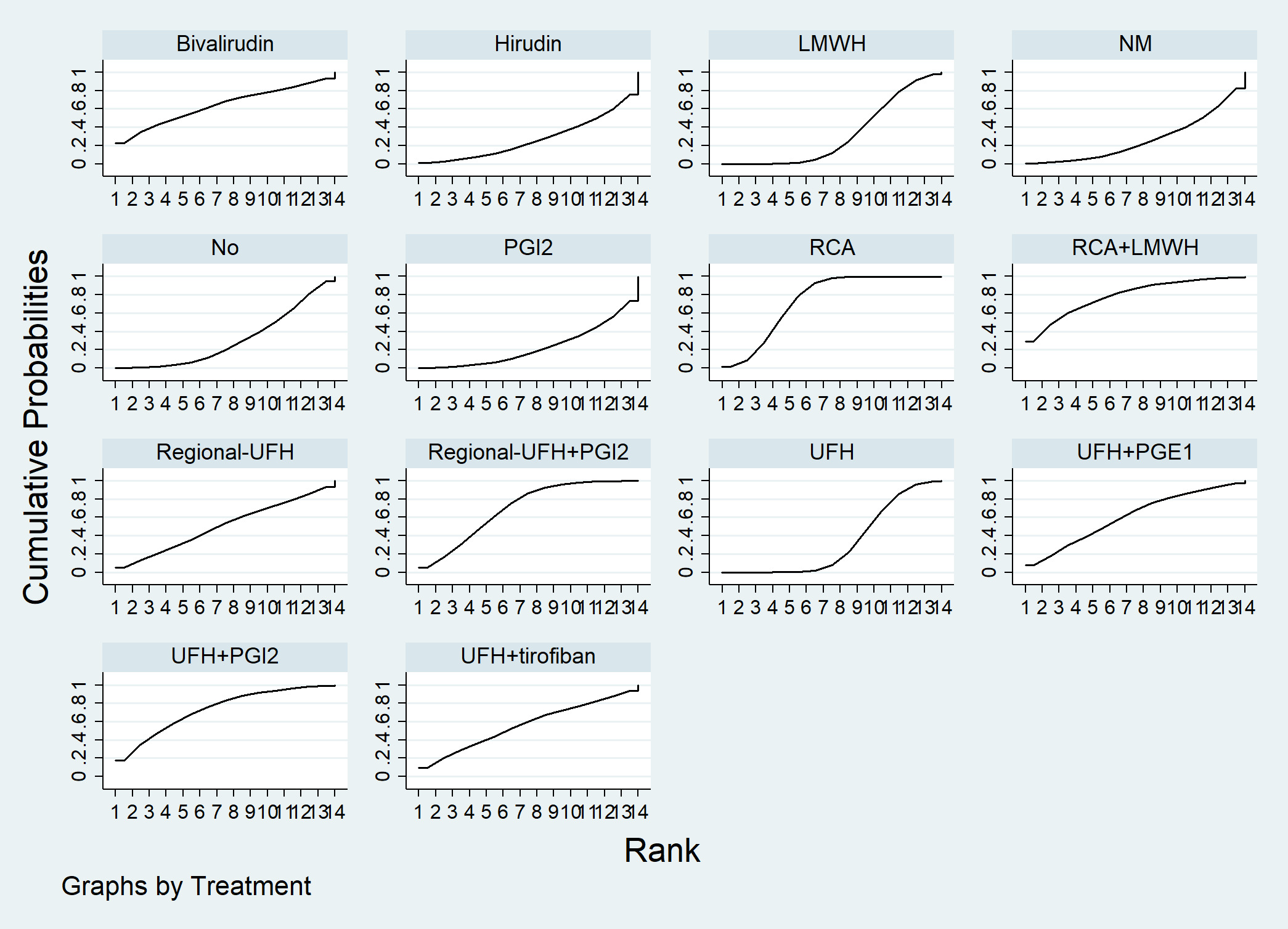


Figure S35. Bleeding events ranking among different anticoagulation options.


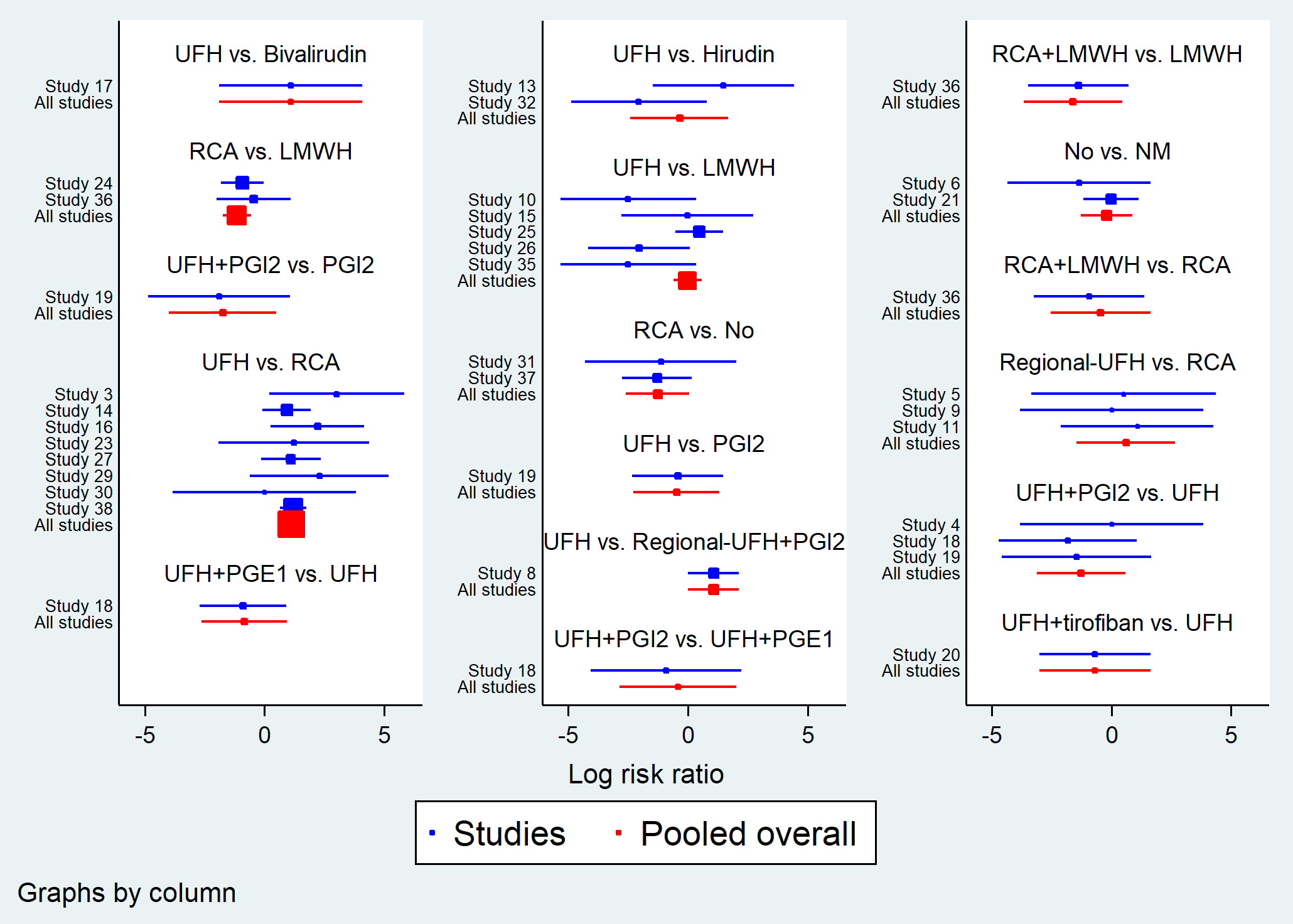


Figure S36. Forest plot in direct comparisons for evaluation of bleeding events.


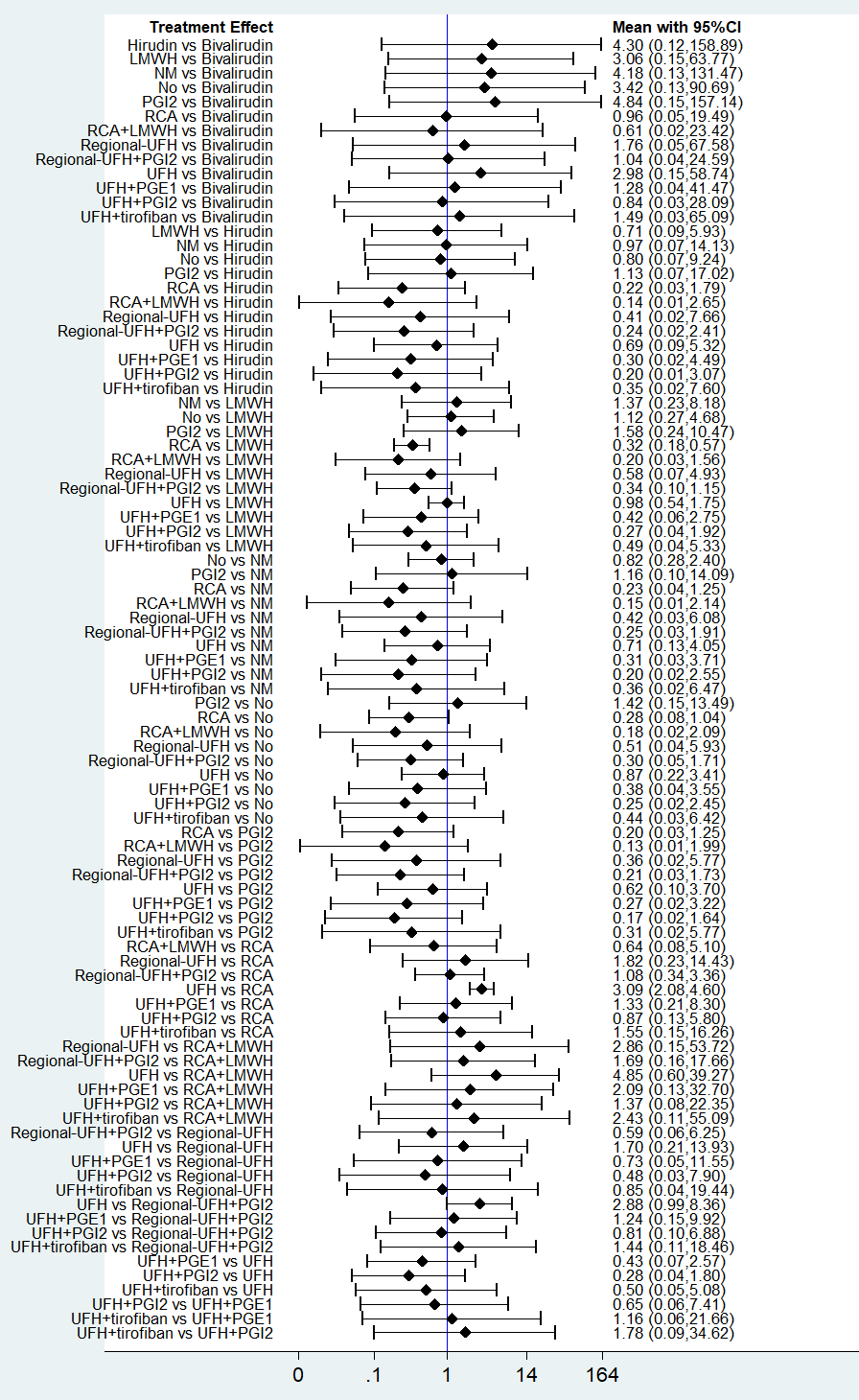


Figure S37. Forest plot of network meta-analysis for bleeding events.


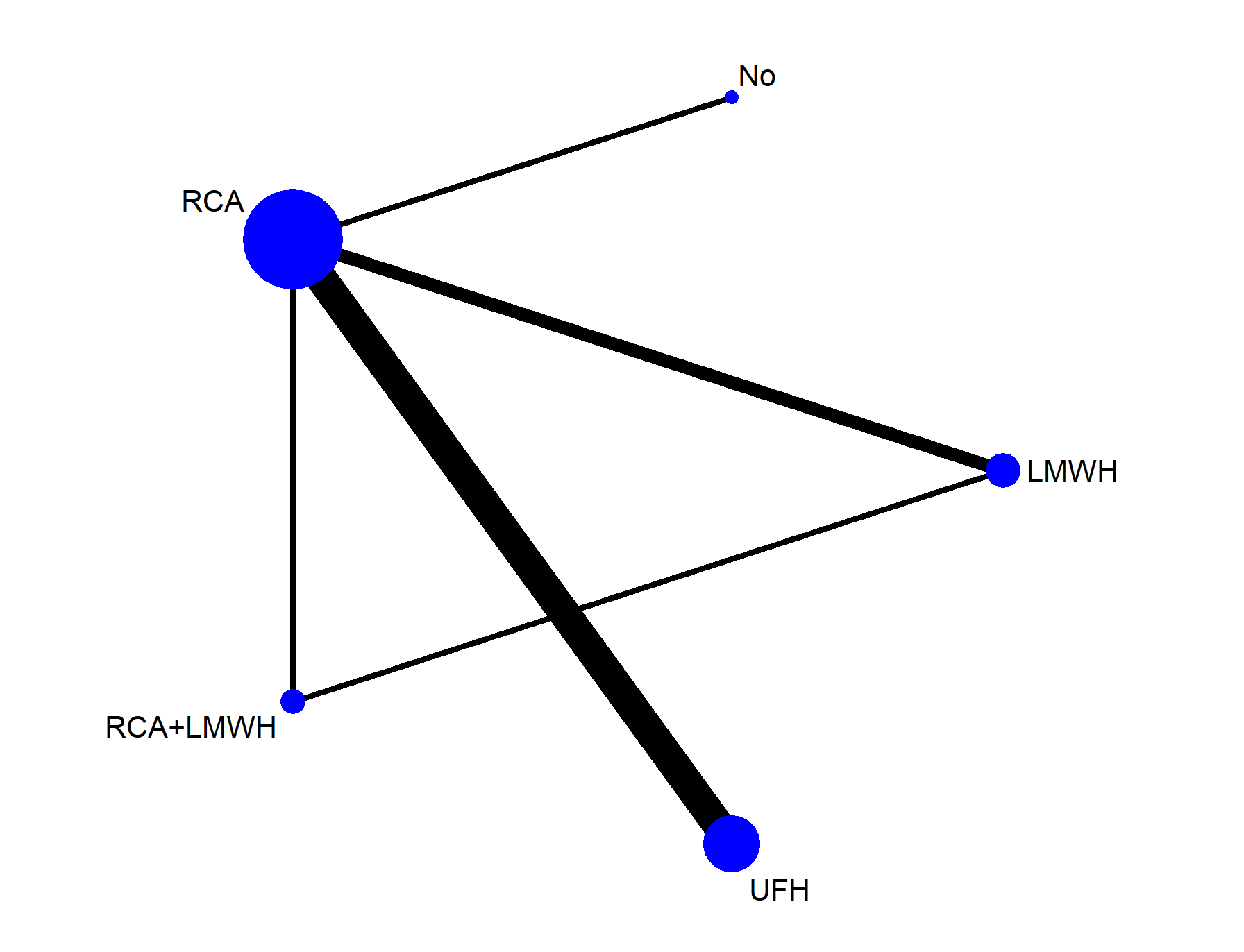


Figure S38. Network geometry of all the included anticoagulation options for evaluating metabolic disturbance.


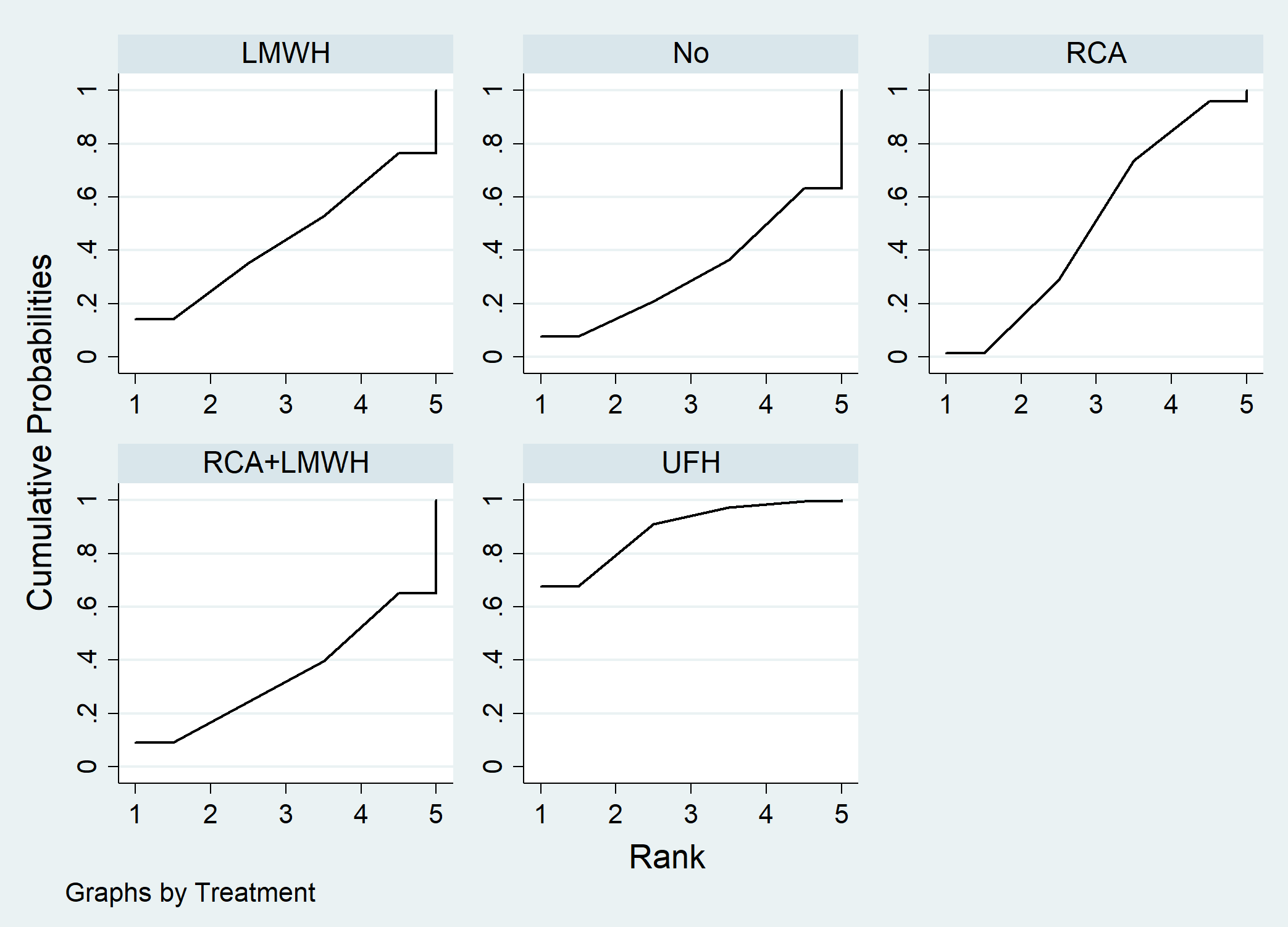


Figure S39. Metabolic disturbance ranking among different anticoagulation options.


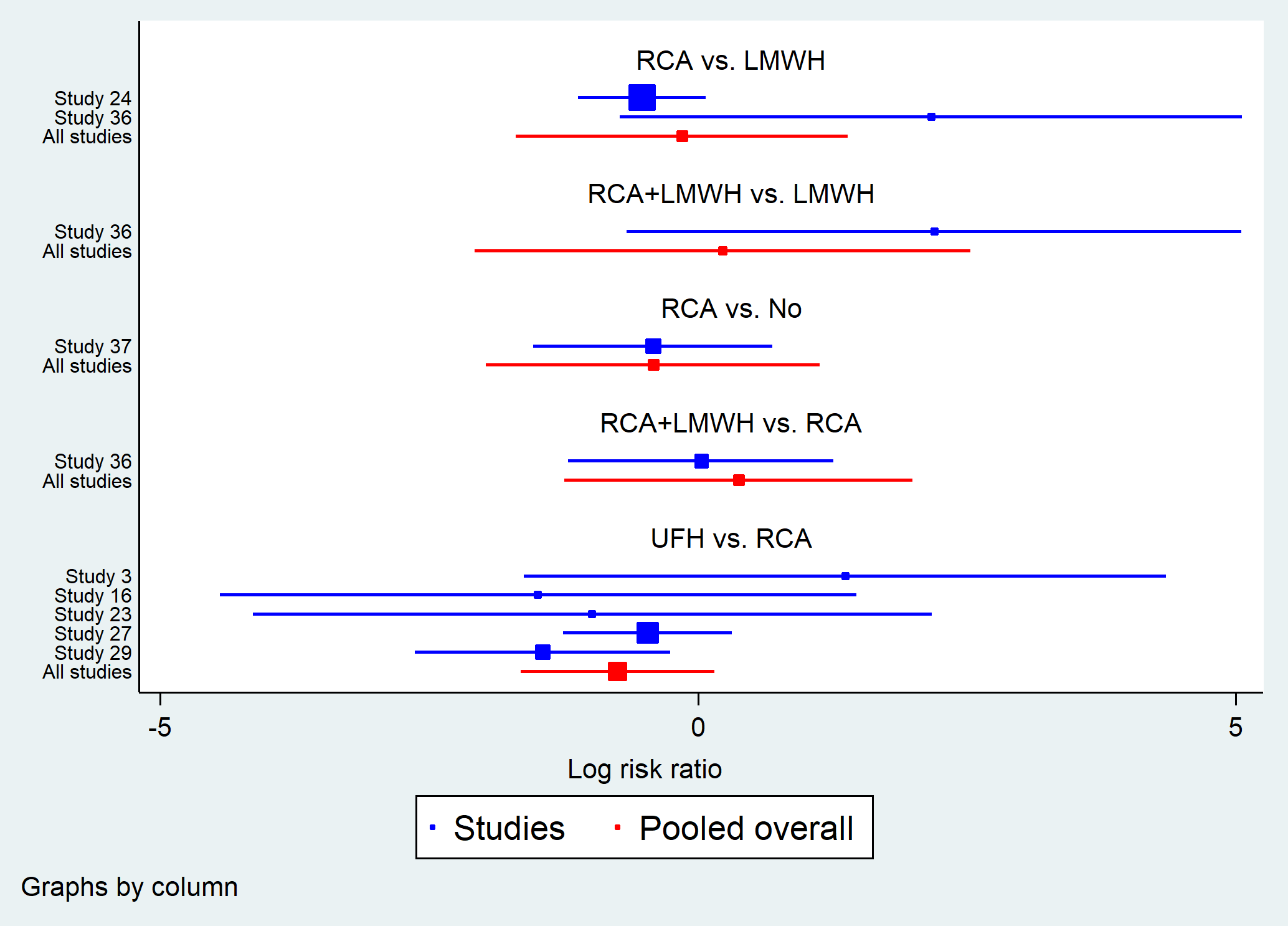


Figure S40. Forest plot in direct comparisons for evaluation of recovery of metabolic disturbance.


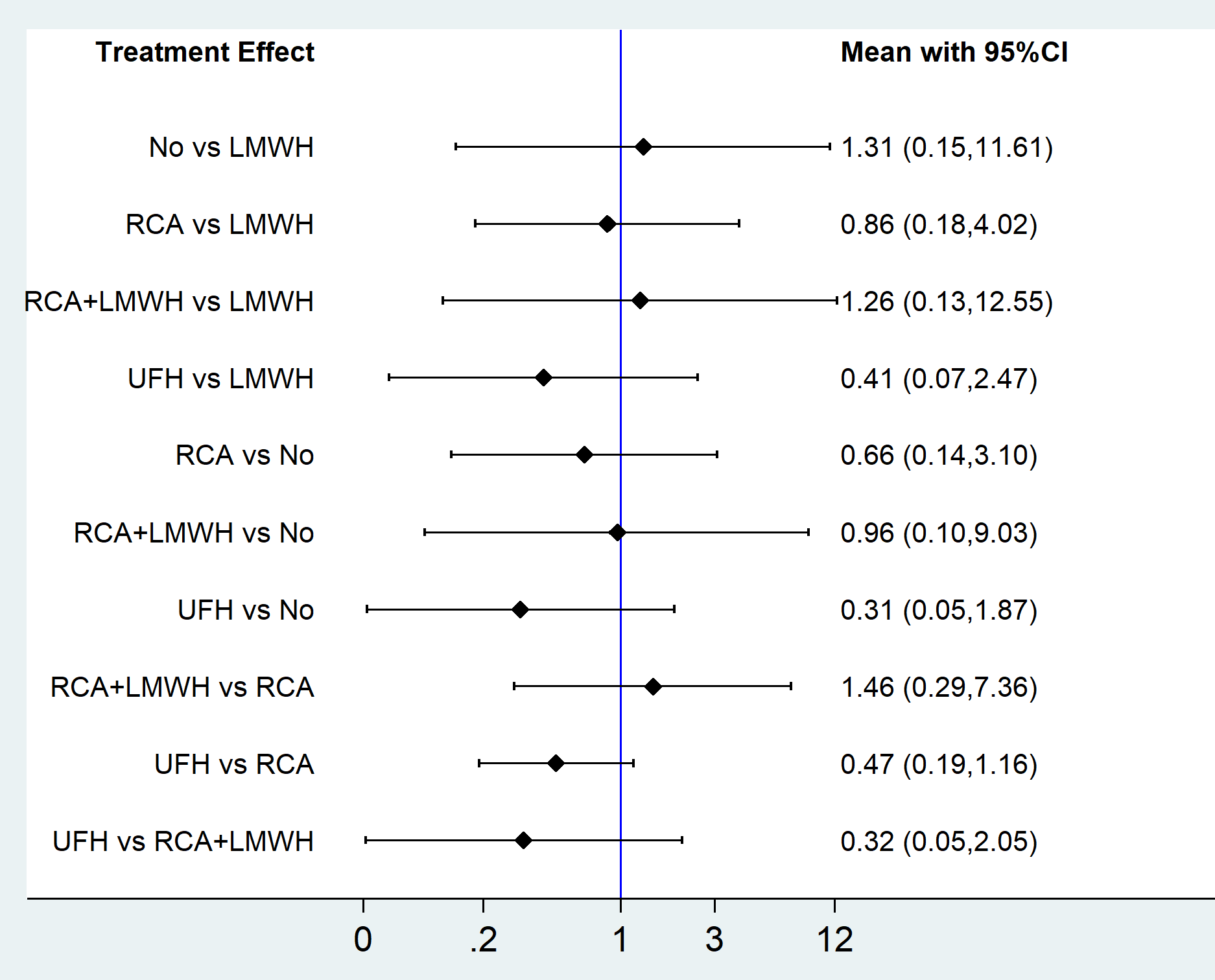


Figure S41. Forest plot of network meta-analysis for reduction of metabolic disturbance.


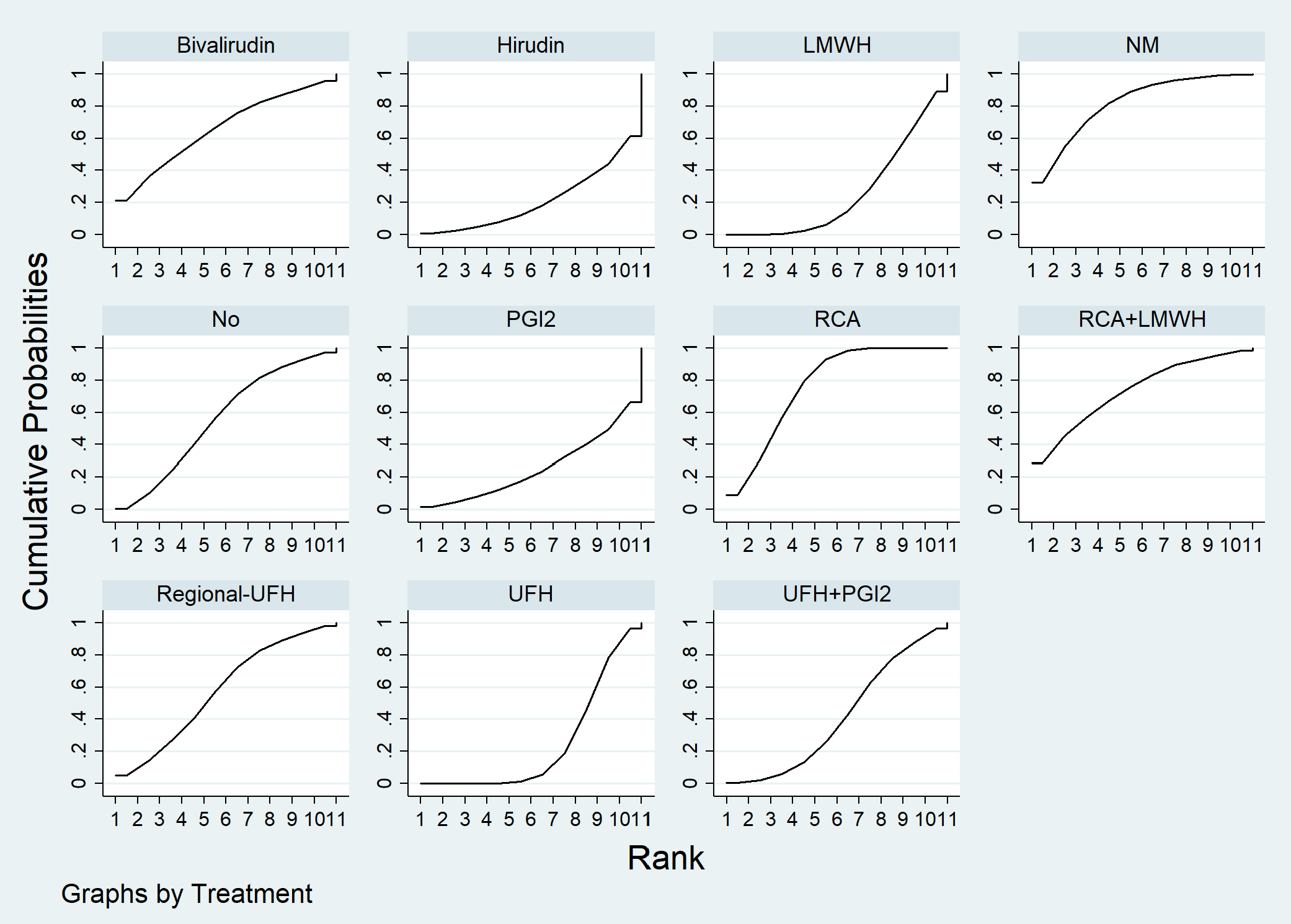


Figure S42. Filter lifespan ranking among different anticoagulation options after excluding 6 trials with a blood flow rate >200 ml/min or <130 mL/min, or without a detail of blood flow rate.


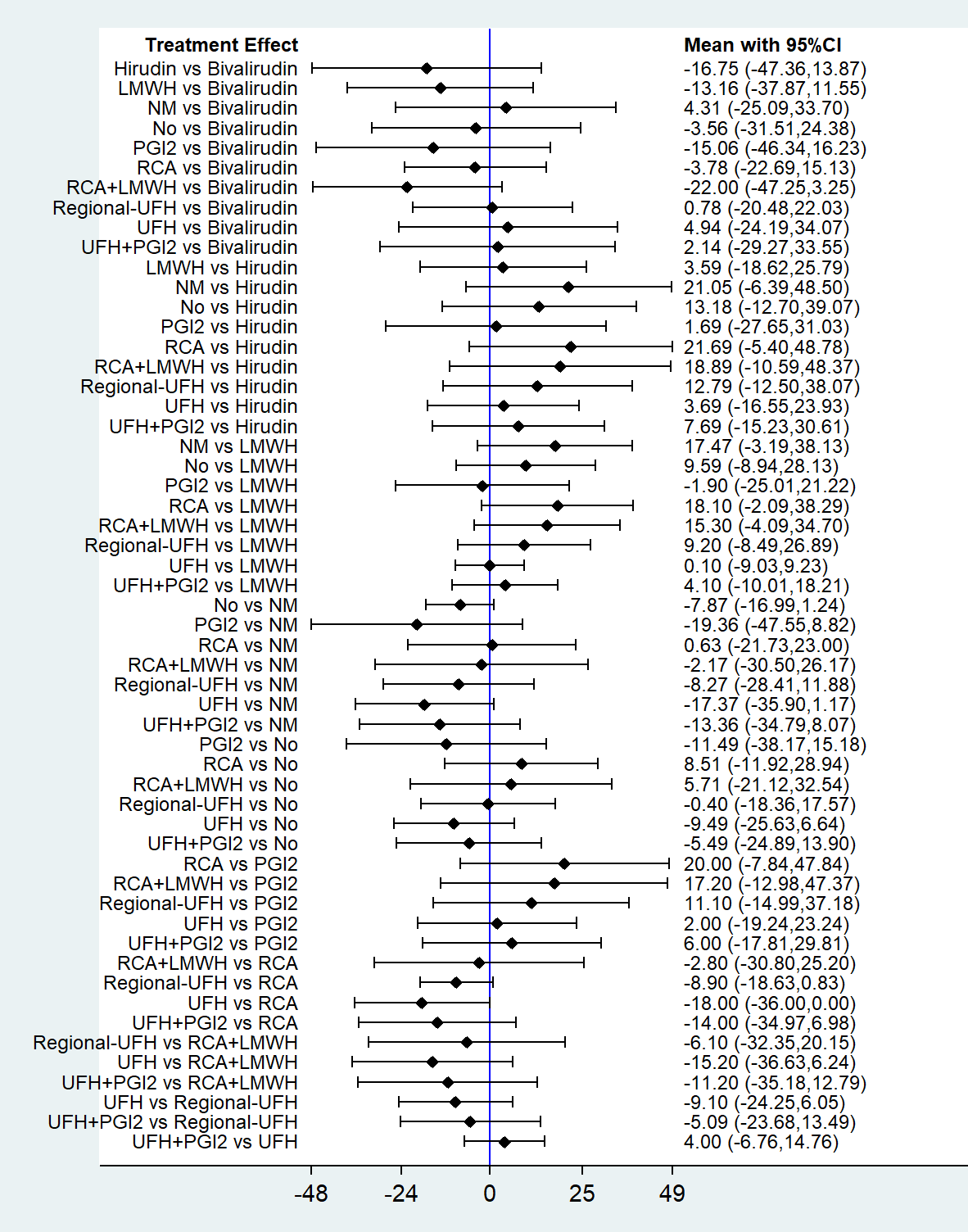


Figure S43. Forest plot of network meta-analysis for filter lifespan after excluding 6 trials with a blood flow rate >200 ml/min or <130 mL/min, or without a detail of blood flow rate.


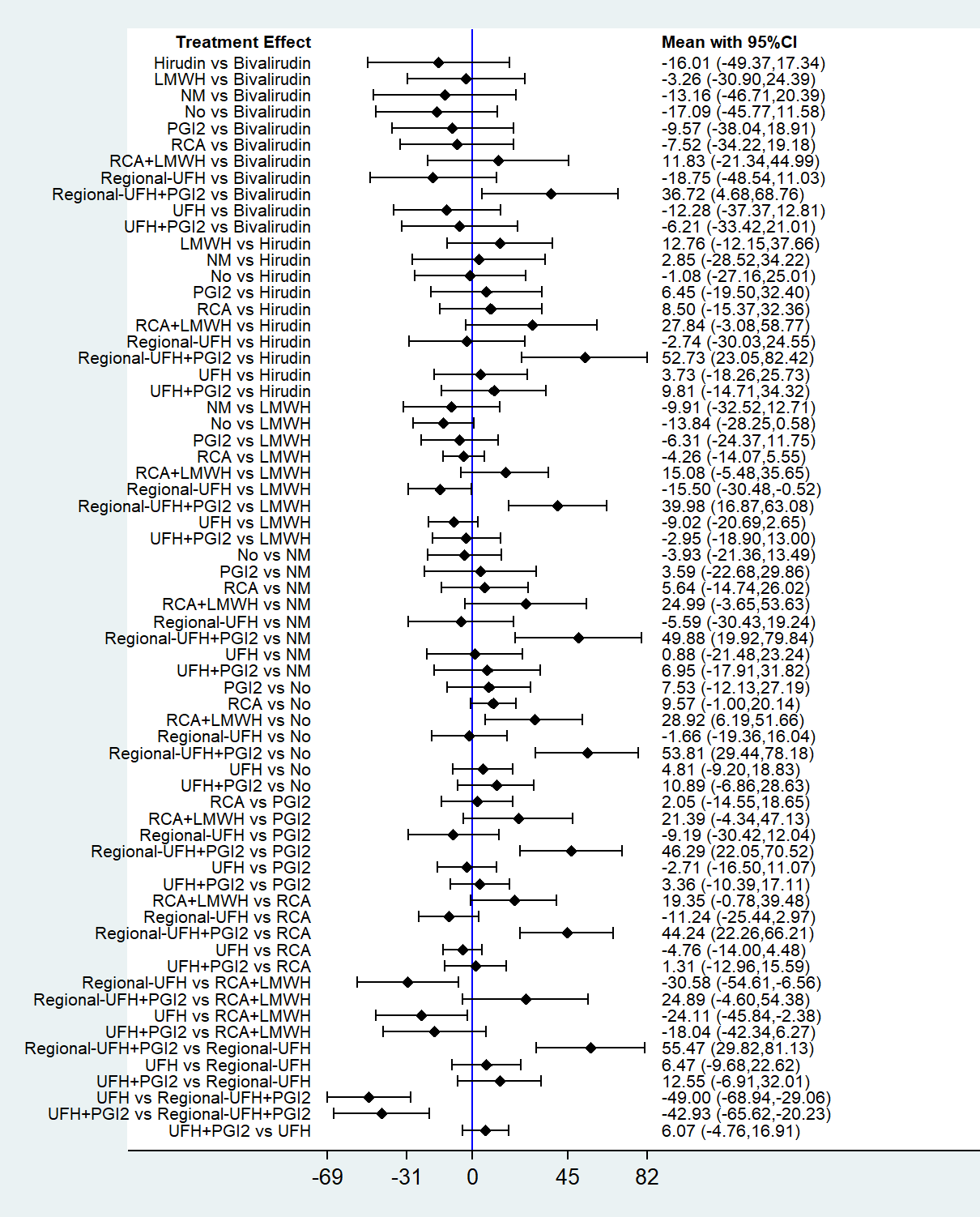


Figure S44. Forest plot of subgroup analysis for populations with CVVH.


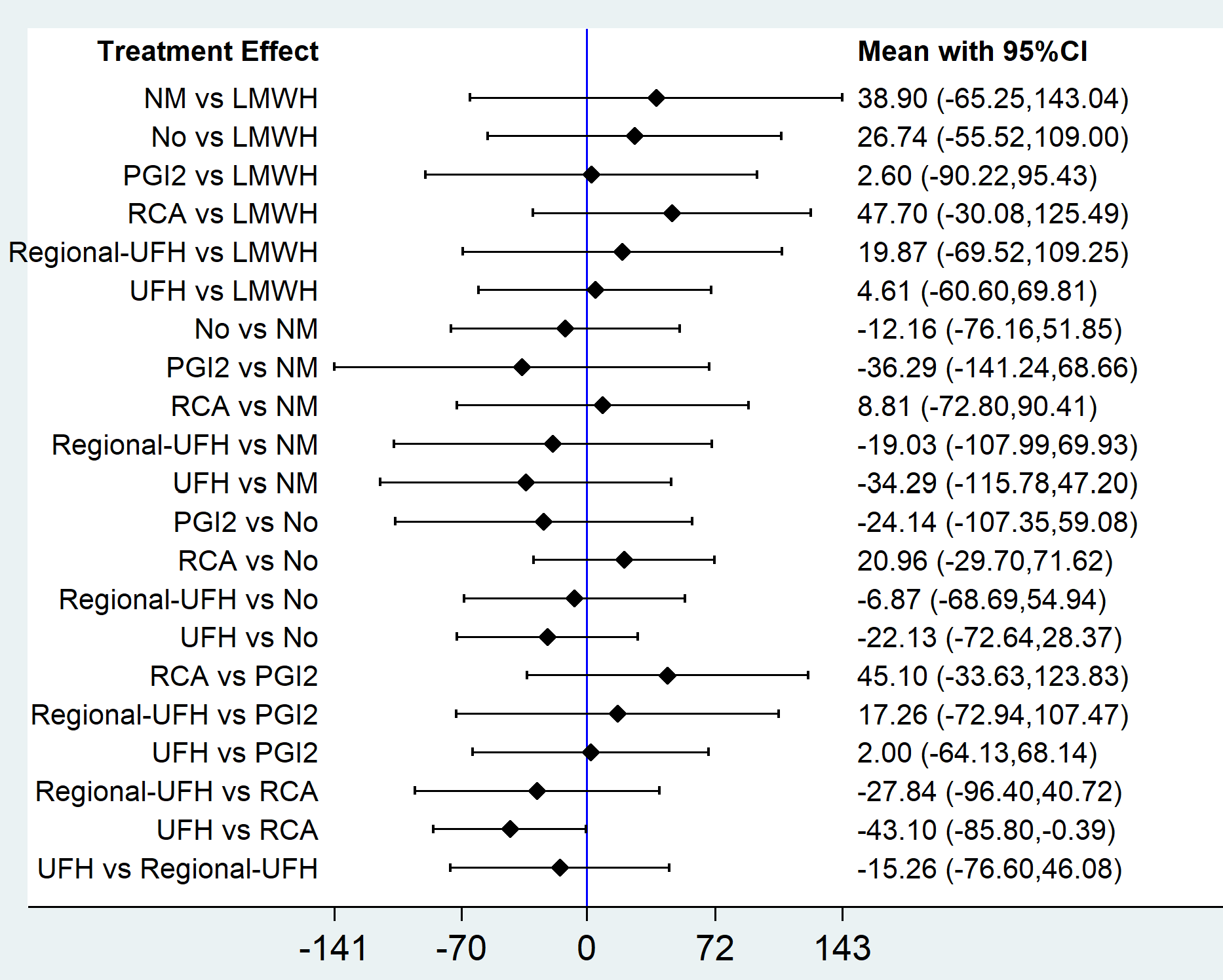


Figure S45. Forest plot of subgroup analysis for populations with CVVHDF.


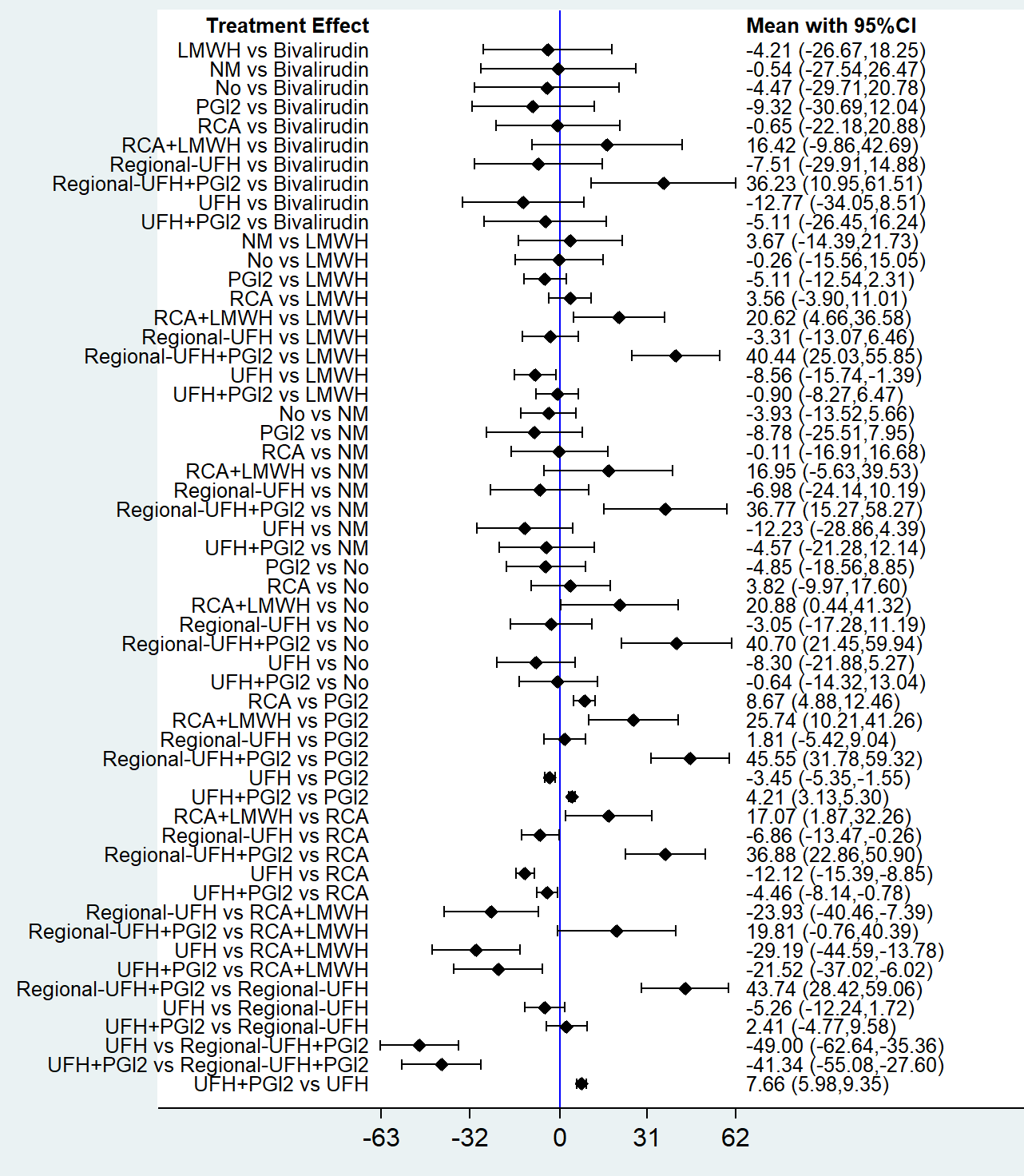


Figure S46. Forest plot of subgroup analysis for populations with pre-dilution.


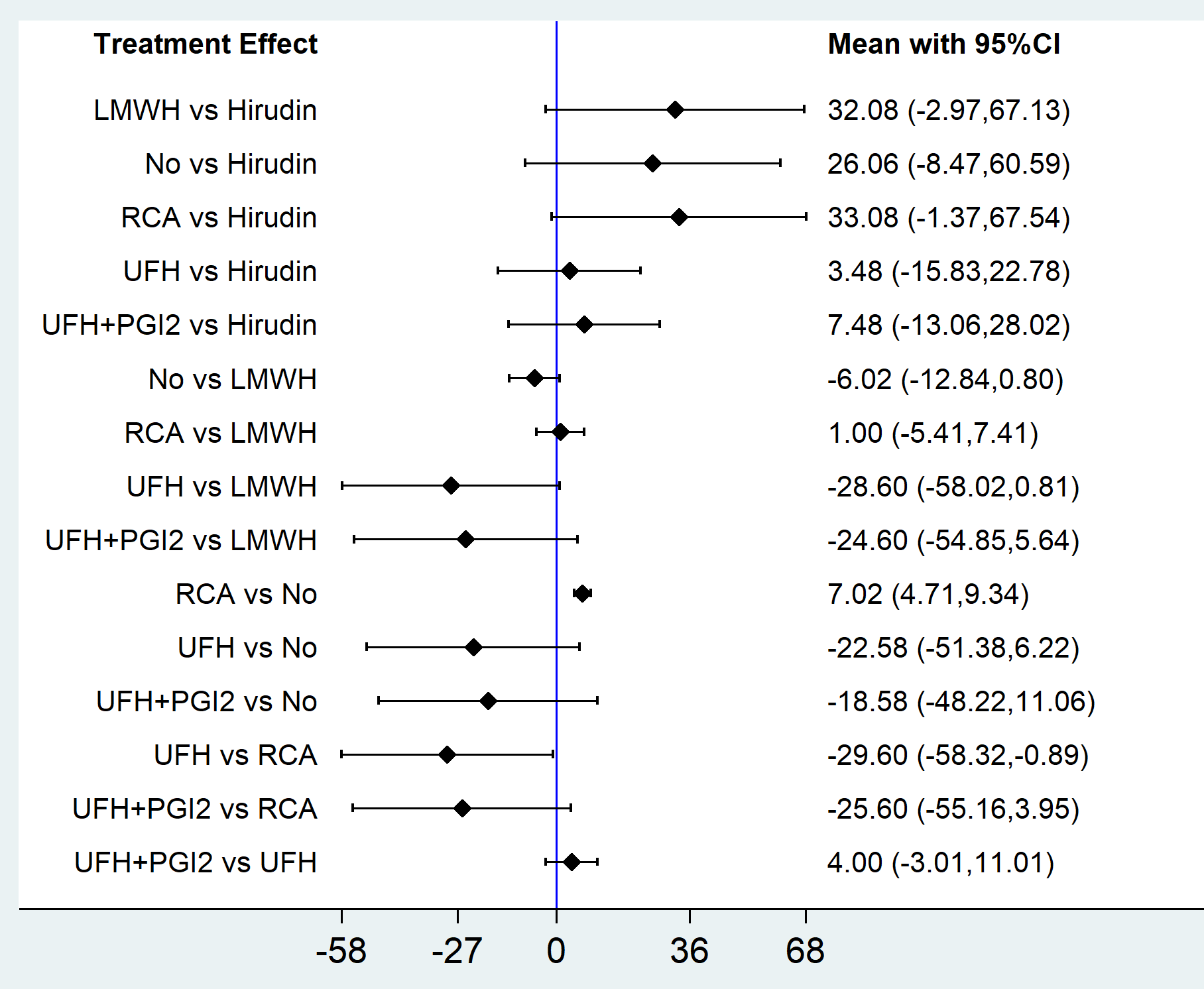


Figure S47. Forest plot of subgroup analysis for populations with post-dilution.
